# Improved Synthesis of Sulfur-Containing Glycosides by Suppressing Thioacetyl Migration

**DOI:** 10.3389/fchem.2020.00319

**Published:** 2020-04-23

**Authors:** Tao Luo, Ying Zhang, Jiafeng Xi, Yuchao Lu, Hai Dong

**Affiliations:** ^1^Key Laboratory for Large-Format Battery Materials and System, Ministry of Education, School of Chemistry & Chemical Engineering, Huazhong University of Science and Technology, Wuhan, China; ^2^Analysis Center of College of Science & Technology, Hebei Agricultural University, Huanghua, China

**Keywords:** sulfur-containing glycoside, acetyl group migration, 4-deoxy glycoside, 2,4-dideoxy-glycoside, desulfurization

## Abstract

Complex mixtures were often observed when we attempted to synthesize 4-thio- and 2,4-dithio-glycoside derivatives by double parallel and double serial inversion, thus leading to no or low yields of target products. The reason was later found to be that many unexpected side products were produced when a nucleophile substituted the leaving group on the substrate containing the thioacetate group. We hypothesized that thioacetyl migration is prone to occur due to the labile thioacetate group even under weak basic conditions caused by the nucleophile, leading to this result. Therefore, we managed to inhibit the generation of thiol groups from thioacetate groups by the addition of an appropriate amount of conjugate acid/anhydride, successfully improving the synthesis of 4-thio- and 2,4-dithio-glycoside derivatives. The target products which were previously difficult to synthesize, were herein obtained in relatively high yields. Finally, 4-deoxy- and 2,4-dideoxy-glycoside derivatives were efficiently synthesized through the removal of thioacetate groups under UV light, starting from 4-thio- and 2,4-dithio-glycoside derivatives.

**Graphical Abstract:**
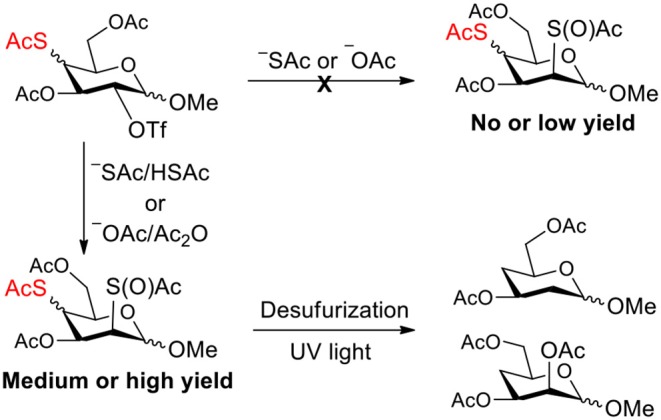
Improved synthesis of sulfur-containing glycosides and the further synthesis of deoxyglycosides using them.

## Introduction

**Scheme 1:**
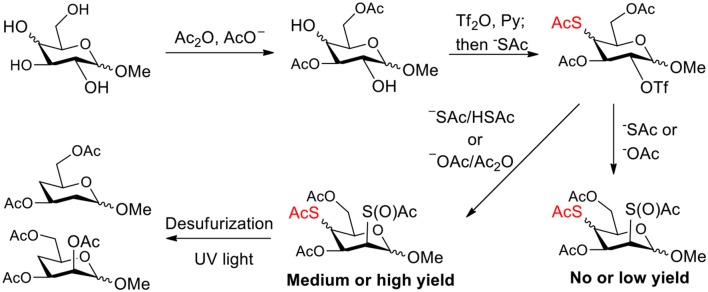
Improving the synthesis of 4-thio- and 2,4-dithio-glycoside derivatives by suppressing thioacetyl migration.

The synthesis of deoxysugars has drawn increasing attention due to their biological importance (Weymouth-Wilson, 1997; Langenhan et al., 2005; Li et al., 2010; Zou et al., 2012; Balmond et al., 2014; Issa and Bennett, 2014; Thoden and Holden, 2014; Zhu et al., 2014; Elshahawi et al., 2015; Sau et al., 2017; Zhang et al., 2019). The synthesis of 4-deoxysugars drew attention because they are expected to express a variety of biological activities including angiogenesis inhibitory activities (Furuta et al., 1979; van Wijk et al., 2010, 2013; Valueva et al., 2011). 4-Deoxysugars are capable of acting as chain terminators for oligosaccharide biosynthesis with 1–4 glycosidic linkages. In order to achieve a siteselective deoxy product starting from a naturally abundant sugar, multistep protection/deprotection sequences and harsh reduction conditions are usually required (Arita et al., 1972; Rasmussen, 1980; Haque et al., 1986; Lin et al., 1989; Raju et al., 2009; Zou et al., 2012). A method has been developed toward direct synthesis of 4-deoxy pyranosides by two steps, site-selective toluoylation of 4-OH of free pyranosides and subsequent reductive deacyloxylation (Yanagi et al., 2019). However, the catalyst for the toluoylation is not readily available, and the yield for deacyloxylation is low (38–61%). Recently, we have developed an efficient method for the synthesis of deoxy glycosides through UV light promoted desulfurization of sulfur-containing glycosides (Ge et al., 2017a, 2019). The efficiency of obtaining siteselective sulfur-containing glycosides is the key to this approach (Ge et al., 2017a,b). We have been developing methods for the synthesis of sulfur-containing carbohydrate (Ren et al., 2015; Wu et al., 2015; Ge et al., 2017a,b; Norberg et al., 2017), since they can be used as tools for model studies or even therapeutic intervention (Crich and Li, 2007; Sakamoto et al., 2009; Caraballo et al., 2010; Baryal et al., 2013; Daly et al., 2013; Jana and Misra, 2013; Zeng et al., 2013). The introduction of sulfur into a carbohydrate molecule usually proceed through substitution of the leaving group with a thioacetate nucleophile. However, unexpected side reactions were often observed during the substitution process due to the existence of thioacetyl group (Knapp et al., 1992; Pei et al., 2006, 2007; Chen and Withers, 2010), which puzzled us until we thought that thioacetyl group migration cause these side reactions (Zhou et al., 2014). In this study, we initially attempted to synthesize 4-thio- and 2,4-dithio-glycoside derivatives by double parallel and double serial inversion (Dong et al., 2007a). However, no or low yields of target products due to complex side reactions. With methyl 3,6-di-OAc-α-mannoside as a starting material, E2 elimination products were obtained due to the axial 2-OTf leaving group and the steric hindrance of 1-OMe groups. With methyl 3,6-di-OAc-β-mannoside as a starting material, the inversion reactivity at the 2-position showed slightly higher than that at the 4-position due to that the axial 2-OTf leaving group can be attacked directly, leading to the failure of the double serial inversion. With methyl 3,6-di-OAc glucosides and galactosides as starting meterials, it was found that many unexpected side products were produced when a nucleophile substituted the leaving group on the substrate containing an thioacetate group. We hypothesized that thioacetyl group migration causes these unexpected side products and are due to the labile thioacetate group even under weak basic conditions caused by the nucleophile. Therefore, we managed to inhibit the generation of thiol groups from thioacetate groups by the addition of an appropriate amount of conjugate acid/anhydride to the reaction system, successfully improving the synthesis of 4-thio- and 2,4-dithio-glycoside derivatives (Scheme 1). Finally, 4-deoxy- and 2,4-dideoxy-glycoside derivatives were efficiently synthesized through the removal of thioacetate groups under UV light, starting from 4-thio- and 2,4-dithio-glycoside derivatives.

## Results and Discussion

**Scheme 2:**
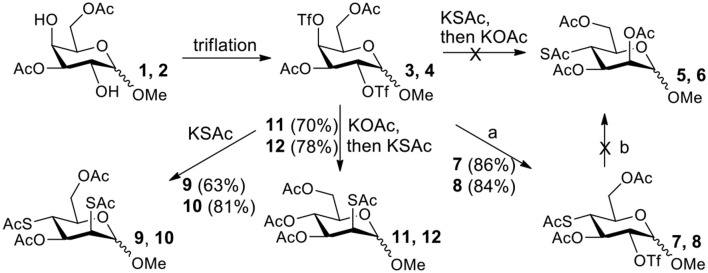
Attempts to synthesize methyl 2-thio-, 4-thio- and 2, 4-dithio-a/β-D-mannopyranosides (Wu et al., 2015): (a) KSAc, MeCN, rt, 0.5 h; (b) KOAc, DMF or MeCN, rt, 48 h, a complex mixture.

The synthesis efficiencies of 2-thio-, 4-thio- and 2,4-dithio-glycosides are key to obtaining 2-deoxy-, 4-deoxy- and 2,4-dideoxy-glycosides by desulfurization (Ren et al., 2015; Wu et al., 2015; Ge et al., 2017a,b; Norberg et al., 2017). We have developed several efficient methods to synthesize glycosides in which both 3- and 6-positions were protected (Ren et al., 2014b; Xu et al., 2016; Zhang et al., 2016; Lv et al., 2018a,b, 2019). One of the methods using acetate (Ren et al., 2014b) or benzoate (Zhang et al., 2016) as a catalyst is particularly convenient and environmentally friendly. Based on this, we conceived to synthesize 4-thio- and 2,4-dithio-glycosides by double parallel or double serial inversion strategies (Dong et al., 2007a) starting from methyl 3,6-OAc glycosides. Methyl 3,6-OAc glycosides can be efficently obtained by selective acetylation of free methyl glycosides catalyzed by the acetate anion (Ren et al., 2014b), and be triflated to afford 2,4-OTf intermediates. Then the intermediates can be allowed to react with thioacetate anion in the double parallel inversion to give 2,4-dithio-glycoside derivatives, or sequentially react with thioacetate/acetate anion or acetate/thioacetate anion in the double serial inversion to give 2-thio- or 4-thio-glycoside derivatives. In our previous attempts to obtain 2-thio-, 4-thio- and 2,4-dithio-mannosides (Scheme 2) (Wu et al., 2015), the 2,4-dithio- and 2-thio-α/β-D-mannoside derivatives **9**/**10** and **11**/**12** were efficiently synthesized while the attempts to synthesize 4-thio-mannoside derivatives **5**/**6** failed. A complex mixture was obtained when the triflated intermediate **3**/**4** was treated with KSAc to substitute its 4-OTf, followed by the substitution of its 2-OTf with KOAc. In order to investigate the cause, we repeated this reaction. The investigation indicated that the substitution of the 4-OTf of **3**/**4** with KSAc in acetonitrile proceeded very well so as to afford the 4-thioacetate intermediate **7/8** in a high yield. However, A complex mixture was observed when the intermediate **7/8** was treated with KOAc whether in aceonitrile or in DMF. Withers encountered a similar dilemma when he attempted to synthesize *p*-nitrophenyl 4-thio-β-D-mannopyranoside by the double serial inversion (Chen and Withers, 2010). He proposed that the thioacetate group is usually labile even under weak basic conditions so as to cause a number of side reactions. Based on our previous studies on thioacetyl migration (Zhou et al., 2014), we guessed that thio group should be readily produced from the deacetylation of thioacetate under basic condition and further lead to acetyl migration, oxidation, and inversion products.

**Scheme 3:**
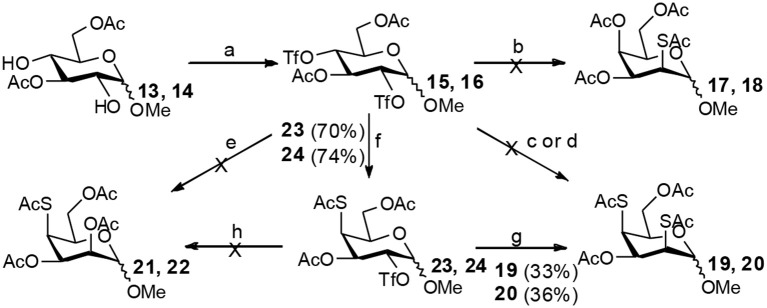
Attempts to synthesize methyl 2-thio-, 4-thio- and 2, 4-thio-a/β-D-talosides: (a) i: TBAOAc, Ac_2_O, MeCN, rt, 24 h; ii: Tf_2_O, pyridine, DCM, −20–10°C, 3 h; (b) i: KOAc, DMF, rt, 1 h; ii: KSAc, DMF, 40°C, overnight (**17**, 11%, **18**, <5%); (c) KSAc, DMF, rt, 48 h, complex mixture; (d) TBASAc, PhMe, rt, 7 d, <10% yield; (e) i: KSAc, DMF, rt, 1 h; ii: KOAc, DMF, 40°C, overnight, complex mixture; (f) TBASAc, PhMe, rt, 1 h, over 3 steps; (g) KSAc, DMF, 40°C, overnight; (h) KOAc, DMF, 40°C, overnight, complex mixture.

It is more difficult to synthesize methyl 2-thio-, 4-thio- and 2, 4-thio-α/β-D-talosides through the double parallel and double serial inversion (Scheme 3). Methyl 3,6-di-OAc-α/β-D-glucoside **13**/**14** can be synthesized in a high yield by regioselective acetylation of free methyl α/β-D-glucoside (Ren et al., 2014b), followed by triflation to give triflated intermediate **15**/**16**. The intermediate **15**/**16** was expected to be sequentially substituted with KOAc and KSAc to give 2-thio-α/β-D-taloside **17**/**18**, to be substituted with an excess amount of KSAc to give 2,4-dithio-α/β-D-taloside **19**/**20**, and to be sequentially substituted with KSAc and KOAc to give 4-thio-α/β-D-taloside **21**/**22**. However, no or very low yields were obtained in all these reactions due to the formation of complex mixtures. The reason was supposed to be due to the neighboring group participation (3-OAc attacking 2 or 4-position) (Dong et al., 2007a, 2008b) and the instability of the thioacetate group under even weak basic conditions. Then the intermediate **15**/**16** was substituted with TBASAc in toluene to supress neighboring group participation, affording 4-SAc intermediate **23**/**24** in a high yield. The isolated **23**/**24** further reacted with KSAc in DMF to give the 2,4-di-SAc taloside derivative **19**/**20** in a yield of 33/36%. The attempts to obtain **21**/**22** by the inversion of **23**/**24** with KOAc in DMF still failed.

**Scheme 4:**
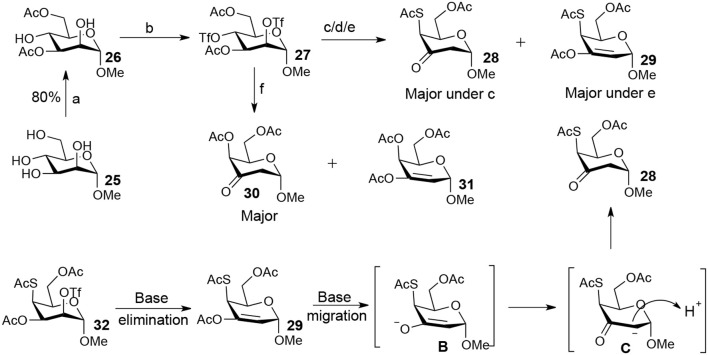
Attempt to synthesize methyl 2-thio-, 4-thio- and 2, 4-dithio-α-D-galactosides starting from methyl α-D-mannoside: (a) i: Bu_2_SnO, MeOH, reflux 2 h; ii: Ac_2_O, MeCN, 0°C to rt, 12 h; (b) Tf_2_O, pyridine, DCM, −20–10°C, 3 h; (c) KSAc (5.0 eq), DMF, rt, 24 h; (d) TBASAc (2.0 eq), PhMe, rt, 48 h; (e) KSAc (1.2 eq), DMF, rt, 48 h; (f) KOAc (1.2 eq), DMF, rt, 48 h.

The attempt to synthesize methyl 2-thio-, 4-thio- and 2, 4-thio-α-D-galactosides through double parallel and double serial inversion failed (Scheme 4). The 3,6-di-OAc-α-D-mannoside **26** was obtained in 80% yield by organotin-mediated regioselective acetylation (Dong et al., 2007b) of free methyl α-D-mannoside **25**. Triflation of **26** afforded triflated intermediate **27**. Treatment of the intermediate **27** with 5.0 equiv of KSAc in DMF for 12 h provided a major product **28** and a minor product **29**. However, it was observed (by TLC plate) that **29** was first formed and then slowly converted to **28** with time. Treatment of **27** with 2.0 equiv of TBASAc in toluene for 48 h gave a mixture of half and half of **28** and **29**. Treatment of **27** with 1.2 equiv of KSAc in DMF for 48 h provided a major product **29**. Similarly, when **27** was treated with 1.2 equiv of KOAc in DMF for 48 h, a major product **30** was isolated. The isolated other product proven to be a mixture containing **31** (Figure S1 in Supplementary Material). Obviously, the substitution of the 4-OTf of **27** by thioacetate could successfully produce the intermediate **32**. However, the substitution of the axial 2-OTf of **32** by thioacetate or acetate was difficult due to the steric hindrance of 1-OMe in this case, thus leading to the E2 elimination and the elimination product **29** under basic conditions. Under this basic conditions, **29** slowly converted to **28** going through migration intermediates **B** and **C** with time.

**Scheme 5:**
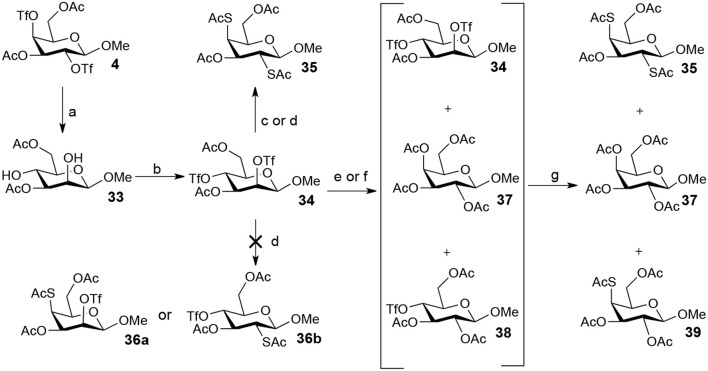
Attempt to synthesize methyl 2-thio-, 4-thio- and 2, 4-thio-β-D-galactoside: (a) TBANO_2_, PhMe, rt, 6 h, 70%; (b) Tf_2_O, pyridine, DCM, −20–10°C, 3 h; (c) TBASAc (5.0 eq), MeCN, rt, 2 h, **35** (84%); (d) TBASAc (1.0 eq), MeCN, 0°C, 4 h, **35** (38%); (e) TBAOAc (2.0 eq), MeCN, rt, 2 h; (f) TBAOAc (2.0 eq), MeCN, 0°C, 4 h; (g) TBASAc (3.0 eq), MeCN, 0°C-rt, 3 h, **39** (8% from b-e-g, 48% from b-f-g).

The attempt to abtain methyl 2-thio-, 4-thio- and 2, 4-thio-β-D-galactosides starting from methyl 3,6-di-OAc-β-manopyranoside **33** showed better results (Scheme 5). Substitution of **34** (the triflated product of **33**) with 5.0 equiv of TBASAc in MeCN at room temperature led to a 84% yield of methyl 2,4-di-thioacetate-galactoside **35**. Unexpectedly, neither intermediaite **36a** (the 4-OTf of **34** substituted by thioacetate) nor intermediate **36b** (the 2-OTf of **34** substituted by thioacetate) could be observed under the conditions that 1.0 equiv of TBASAc was used instead, and **35** was still obtained. Usually, 4-OTf showed higher reactivity than 2-OTf when substituted on a glycoside ring. However, the substitution of 4-OTf of **34** is disfavored due to steric hindrance of 2-OTf in this case. Once 2-OTf of **34** had been substituted by thioacetate, the substitution of 4-OTf would occur immediately due to the disappearance of the steric hindrance from 2-OTf, leading to the formation of **35**. Similarly, the treatment of **34** with 1.0 equiv of TBAOAc in MeCN at room temperature mainly gave product **37**. However, when this reaction was performed at 0°C, intermediate **38** was formed. Consequently, the following addition of 3 equiv of TBASAc led to 4-thioacetate galactoside **39** in 48% yield. However, the treatment of **34** with 1.0 equiv of TBASAc in MeCN at 0°C did not give intermediate **36b**, but gave **35** in 38% yield. While axial triflates can be attacked directly (the antibonding orbital can be approached), the sugar ring has to adopt a different conformation to allow attack on the equatorial triflate (since the antibonding orbital is shielded by the axial substituents on the ring). Lowering the temperature thus may slow down the interconversion of the ring. Thus, the 2-OTf of **34** showed high reactivity on substitution by acetate due to the axial ttiflate leaving group. When the reaction proceeded at room temperature, the product **37** was formed immediately from the intermediate **38**. However, when the reaction proceeded at 0°C, the interconversion of the sugar ring turned very slow, which thus restrained the further substitution of 4-OTf by the acetate. However, thioacetate showed much higher nucleophilicity than acetate since sulfur is a big atom and it will therefore readily have a productive overlap with the antiboding orbital. This high nucleophilicity of thioacetate flattened the difference in reactivity between 2-OTf and 4-OTf. We also proposed that a supramolecular control effect perhaps plays a key role in this process, in which an acetate ion can be accommodated at the center of the β-pyranoside face to produce an anion-carbohydrate complex (Dong et al., 2008a; Ren et al., 2014a), resulting in a higher reactivity of 2-OTf than that of 4-OTf. However, the poor or no supramolecular effect in polar solvent acetonitrile can not fully support this result.

**Figure 1:**
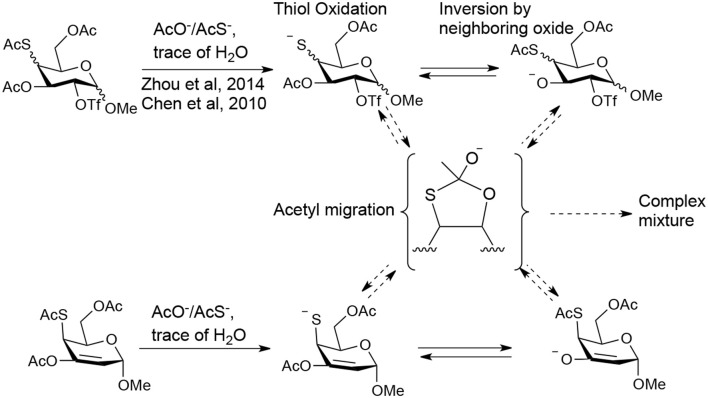
The possible approaches to form the complex mixture due to the existence of thiol.

From these experiments, we noticed that the substitution on a substrate which have already contained a thioacetate group usually led to unexpected side-products. The previous studies (Chen and Withers, 2010; Zhou et al., 2014) have suggested that thioacetate groups are usually labile even under weak basic conditions, and then produce thiol groups, leading to acetyl migration, oxidation, and inversion products (Figure 1). The initial thiol might be generated by intermolecular acetyl migration to a nucleophile (trace of water, dimethylamine in DMF, acetate, or thioacetate) in the reaction mixture. Once a thiol group was formed, the intramolecular acetyl migration from an adjacent acetyl group to the thiol group would occur under even weak basic conditions. If the complex mixtures were indeed caused by thiol group and acetyl migration in these reactions, suppressing such formation of thiol and such migration by adjusting acidic/basic condition may improve these reactions.

Therefore, the substitution on 2-triflated intermediate **7** by acetate/thioacetate was used as a model reaction to test under various acidic/basic conditions (Table 1). Substitution of **7** with acetate in acetonitrile or DMF led to a complex mixture (entry 1). To our delight, with the addition of more and more acetic acid to this reaction system, the target **5** was isolated for better and better yield (entries 2–5). Especially, the yield of **5** when using Ac_2_O as the solvent (entries 6 and 7) was 76–78% as compared with the 50% yield when using acetic acid as the solvent (entry 5). This must be because Ac_2_O greatly inhibited the generation of thiol groups by reacting with thiol groups to form thioacetates. Substitution of **7** with 5 equiv of thioacetate in acetonitrile yielded the target **9** in 56% yield (entry 8). The yield of **9** was increased to 75% when 1.5 equiv of thioacetic acid was added to this reaction system (entry 9). However, the addition of more thioacetic acid decreased the yield of **9** (entries 10 and 11). Substitution of **7** with 5 equiv of thioacetate in DMF in the absence/presence of 1.5 equiv of thioacetic acid yielded **9** in 70/90% yield, respectively (entries 12 and 13). These results indicated that our hypothesis was reasonable. The substitution on a substrate containing a thioacetate group by thioacetate/acetate could be improved by suppressing the formation of thiol and by adjusting the acidity/basicity of the reaction system.

**Table 1:** Optimization of the reaction conditions^a^.

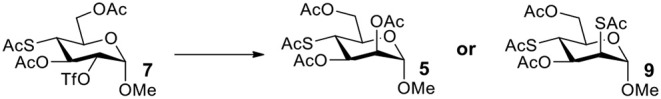				
**Entry**	**Reagent**	**Solvent**	**Condition**	**Yield %**
1	KOAc/TBAOAc	MeCN/DMF	35–60°C, 24 h	Complex mixture
2	KOAc:HOAc = 1:1	MeCN	75°C, 24 h	21 (**5**)
3	KOAc:HOAc = 1:2	MeCN	75°C, 24 h	35 (**5**)
4	KOAc:HOAc = 1:5	MeCN	75°C, 24 h	46 (**5**)
5	KOAc	HOAc	75°C, 24 h	50 (**5**)
6	TBAOAc	Ac_2_O	75°C, 54 h	78 (**5**)
7	KOAc	Ac_2_O	75°C, 54 h	76 (**5**)
8	KSAc	MeCN	50°C, 54 h	56 (**9**)
9	KSAc:HSAc = 1:0.3	MeCN	50°C, 54 h	75 (**9**)
10	KSAc:HSAc = 1:0.5	MeCN	50°C, 54 h	69 (**9**)
11	KSAc:HSAc = 1:1	MeCN	50°C, 54 h	68 (**9**)
12	KSAc	DMF	50°C, 54 h	70 (**9**)
13	KSAc:HSAc = 1:0.3	DMF	50°C, 24 h	90 (**9**)

a *Substrate **7** (50 mg), KOAc, TBAOAc or KSAc (5 equiv), Solvent (1 mL)*.

The synthesis of 2-OAc-4-SAc methyl glycosides **5**, **6**, **21**, and **22** failed in Schemes 2, 3. However, with Ac_2_O used as the reaction solvent instead, **5**, **6**, **21**, and **22** were successfully synthesized in medium to high yields (47–78%) starting from their 2-OTf intermediates **7**, **8**, **23**, and **24** (entries 1–4 in Table 2). The main side-products seemed to be caused by 1-OMe group participation, such as **6a** (Figure S2 in Supplementary Material). The yields of 2,4-di-SAc glycosides **9**, **10**, **19**, and **20** were increased by 9–13% compared to the reaction without the addition of 1.5 equiv of thioacetic acid (entries 5–8). The main side-products seemed to be caused by the oxidation of intra-molecular thiol groups, such as **19a**. Substitution of 6-OTs glycosides **40**, **42**, and **44** with potassium thioacetate in DMF, the yields of 6-SAc glycosides **41**, **43**, and **45** were increased by 10–16% compared to the reaction without the addition of 0.5 equiv of toluene sulfonic acid (entries 9–11).

**Table 2:** Synthesis of 4-thio-glycosides under optimized condition.

**Entry**	**Substrate**	**Product**	**Yields in schemes 2, 3**	**Improved method**
1	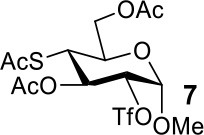	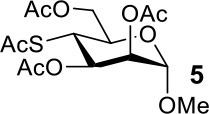	Complex mixture	76%^a^
2	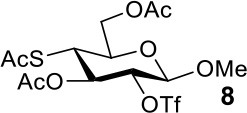	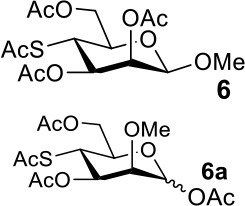	Complex mixture	**6**: 53%^a^ **6a**: 39%^a^
3	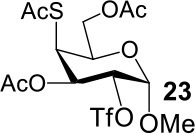	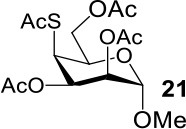	Complex mixture	78%^a^
4	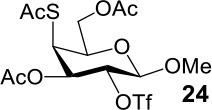	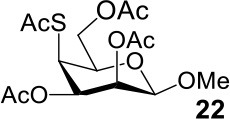	Complex mixture	54%^a^
5	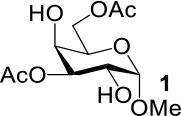	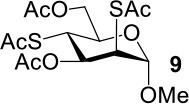	63%	76%^b^
6	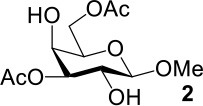	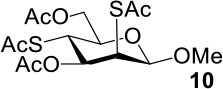	81%	91%^b^
7	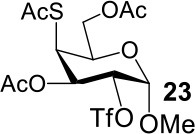	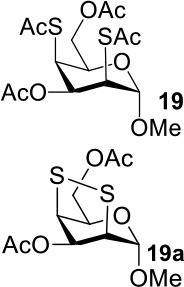	33%	**19**: 54%^c^ **19a**: 40%^c^
8	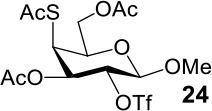	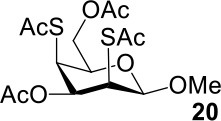	36%	57%^c^
9	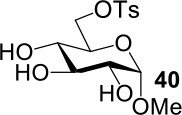	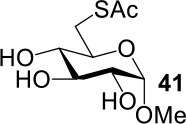	75%^d^	85%^e^
10	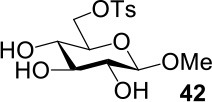	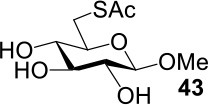	60%^d^	76%^e^
11	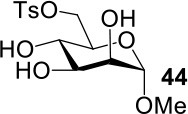	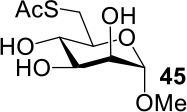	70%^d^	82%^e^

*Reaction condition*:

a *Ac_2_O, KOAc (5.0 equiv), 75°C, 24 h*;

b *i: Tf_2_O, pyridine, DCM, −20–10°C, 3 h; ii: CH_3_CN, KSAc (5.0 equiv), HSAc (1.5 equiv), 50°C, 12–24 h*.

c *CH_3_CN, KSAc (5.0 equiv), HSAc (1.5 equiv), 50°C, 24–48 h*;

d *KSAc (1.5 equiv), DMF, 60°C, 6 h*.

e *KSAc (1.5 equiv), TsOH (0.5 equiv), DMF, 60°C, 6 h*.

**Figure 2:**
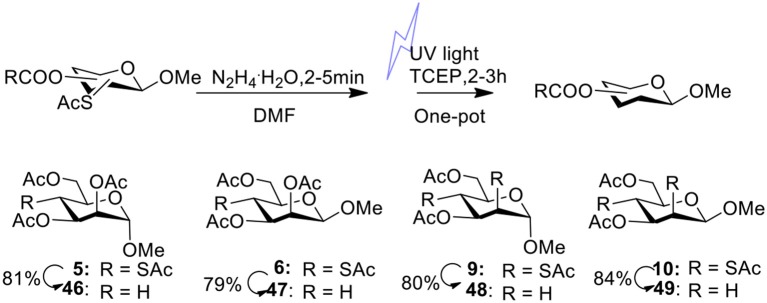
One-pot reaction for removal of thioacetate group by desulfurization under UV light.

In our previous studies on the synthesis of deoxyglycosides by desulfurization under UV light (Ge et al., 2017b, 2019), we didn't obtain 4-deoxymannosidic derivatives because we were unable to obtain 4-SAc mannosides efficiently. With 4-SAc mannosides **5** and **6** in the hands, 4-deoxymannosidic derivatives **46** and **47** were obtained in 81 and 79% yields by our one-pot method removing thioacetate group (Figure 2), respectively. With 2,4-di-SAc mannosides **9** and **10** in the hands, we started to test if 2,4-di-deoxy glycosides could be obtained by simultaneously removing two thioacetate groups in a one-pot method. After optimizing the reaction conditions, substrates **9** and **10** were treated with 2.5 equiv of N_2_H_4_·H_2_O in DMF at room temperature for 4 min, followed by the addition of 3.0 equiv of TCEP^.^HCl, and desulfurization under UV light led to 2,4-di-deoxy glycosides **48** and **49** in 80 and 84% yields, respectively.

## Conclusion

It was attempted to synthesize methyl 2-thio-, 4-thio- and 2,4-dithio-glycosides starting from methyl 3,6-di-OAc glycosides by a double parallel and double serial inversion strategy in this study. Complex mixtures were often observed, thus leading to no or low yields of target products. With methyl 3,6-di-OAc-α-mannoside as a starting material in this strategy, elimination products were obtained due to the steric hindrance of 1-OMe group. With methyl 3,6-di-OAc-β-mannoside as a starting material in this strategy, the slightly higher reactivity of 2-OTf than that of 4-OTf due to the axial 2-OTf leaving group, leads to the failure of the double serial inversion. With methyl 3,6-di-OAc glucosides and galactosides as starting materials, it was found that many unexpected side products were produced when a nucleophile substituted the leaving group on the substrate containing an thioacetate group. The reason is hypothesized that thioacetyl migration is prone to occur due to the labile thioacetate group even under weak basic conditions caused by the nucleophile. Therefore, when substitution of the substrate with an acetate anion, Ac_2_O was used as a solvent to inhibited the generation of thiol groups by reacting with thiol groups to form thioacetates; when substitution of the substrate with a thioacetate anion, an appropriate amount of thioacetic acid was added to the reaction system to adjust the basicity. Consequently, the synthesis of target 4-thio- and 2,4-dithio-glycoside products was successfully improved due to suppressing thioacetyl migration. Finally, 4-deoxy- and 2,4-dideoxy-glycoside derivatives were efficiently synthesized through the removal of thioacetate groups under UV light, starting from 4-thio- and 2,4-dithio-glycoside derivatives.

## Data Availability Statement

All datasets generated for this study are included in the article/Supplementary Material.

## Author Contributions

TL performed the experiments and analyzed the data. YZ and JX prepared some related substrates and analyzed the data. YL and HD came up with the original idea, conceptualized and directed the project, and drafted the paper with the assistance from all co-authors.

## Conflict of Interest

The authors declare that the research was conducted in the absence of any commercial or financial relationships that could be construed as a potential conflict of interest.
