# Supplementary material for: Improved Synthesis of Sulfur-Containing Glycosides by Suppressing Thioacetyl Migration
Source: Front Chem. 2020 Apr 23;8:319. doi: 10.3389/fchem.2020.00319 (PMC7191076; doi:10.3389/fchem.2020.00319)
Supplement: Supplementary file 1 [file Data_Sheet_1.PDF]

Supplementary Material

Improved Synthesis of Sulfur-Containing Glycosides by Suppressing Thioacetyl Migration

Tao Luo,[1] Ying Zhang,[1] Jiafeng Xi,[2] Yuchao Lu,[2,*] and Hai Dong[1,*]

[1] Key Laboratory for Large-Format Battery Materials and System, Ministry of Education, School of Chemistry & Chemical Engineering, Huazhong University of Science & Technology, Luoyu Road 1037, Wuhan, 430074, P. R. China

[2] Analysis Center of College of Science & Technology, Hebei Agricultural University, Huanghua, 061100, P. R. China

Correspondence:

hdong@mail.hust.edu.cn

TABLE OF CONTENTS

General Methods……………………………………………………………………S2

Figure S1……………………………………………………………………………S2

Figure S2……………………………………………………………………………S3

Preparation and characterization for the products……………………………..S3-S11

NMR spectra …. ………………………………………………………… ….S12-S46

1. General Methods.

All commercially available starting materials and solvents were of reagent grade and were dried prior to use. Chemical reactions were monitored with thin-layer chromatography using precoated silica gel 60 (0.25 mm thickness) plates. High-resolution mass spectra (HRMS) were obtained by electrospray ionization (ESI) and Q-TOF detection. Flash column chromatography was performed on silica gel 60 (0.040-0.063 mm). 1H and 13C spectra were recorded with a 400 MHz instrument at 298K in $CDCl_3$, using the residual signals from *d*-chloroform (^{1}H: δ = 7.25 ppm; ^{13}C: δ = 77.2 ppm), as internal standard. Assignments were made by first-order analysis of the spectra, supported by standard ^{1}H–^{1}H correlation spectroscopy (COSY).

2. Figure S1.

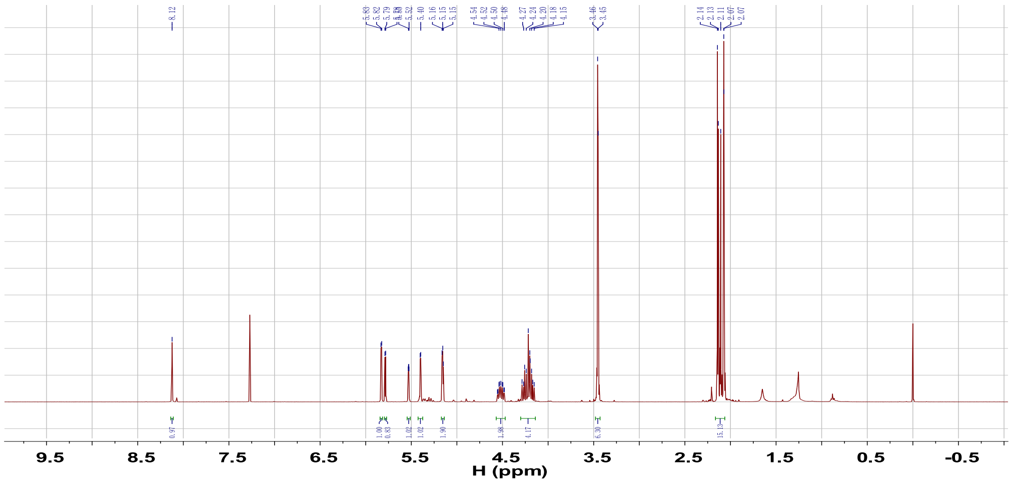

Mass Spectrum List Report

Analysis Info

Analysis Name	D:\Data\Donghai\dong-zhangying20181123-3.d	Acquisition Date	11/23/2018 10:56:35 AM
Method	tune_low_20170906_50-1200.m	Operator	BDAL@DE
Sample Name	dong-zhangying20181123-3	Instrument / Ser#	micrOTOF 10401
Comment			

Acquisition Parameter

Source Type	ESI	Ion Polarity	Positive	Set Nebulizer	0.3 Bar
Focus	Active			Set Dry Heater	200 °C
Scan Begin	50 m/z	Set Capillary	4000 V	Set Dry Gas	4.0 l/min
Scan End	1200 m/z	Set End Plate Offset	-500 V	Set Divert Valve	Waste

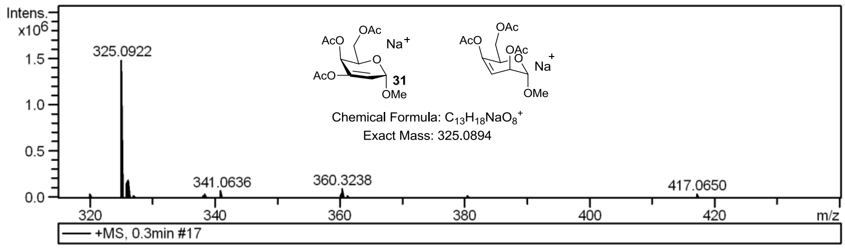

#	m/z	Res.	S/N	I	FWHM
1	325.0922	11737	3774.2	1486126	0.0277
2	326.0931	8395	499.4	195936	0.0388
3	327.0811	3949	88.4	34537	0.0828
4	341.0636	8156	204.9	76106	0.0418
5	360.3238	7867	288.2	99302	0.0458
6	417.0650	7835	150.9	43097	0.0532
7	418.0677	7736	31.9	9105	0.0540
8	419.0640	8288	21.0	5959	0.0506

The ^{1}H NMR indicates a mixture of two compounds. The HRMS may indicate the existence of **31**.

3. Figure S2.

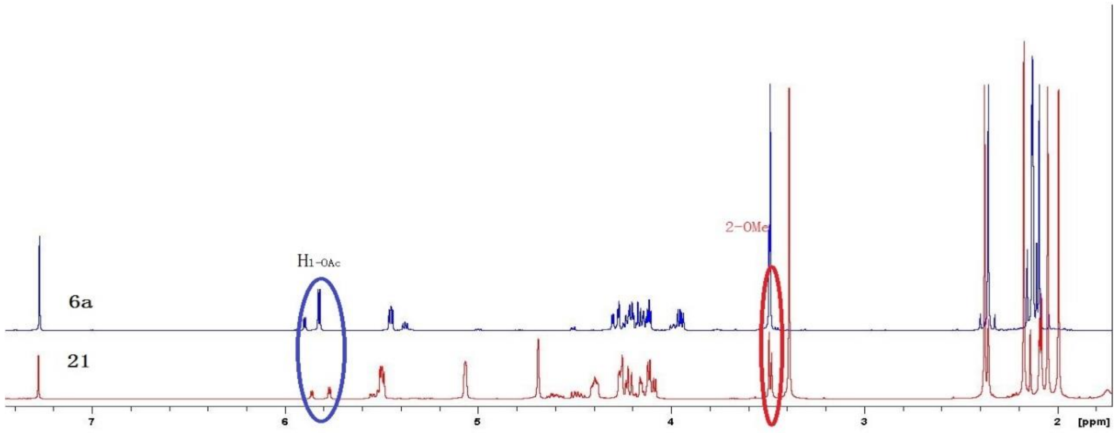

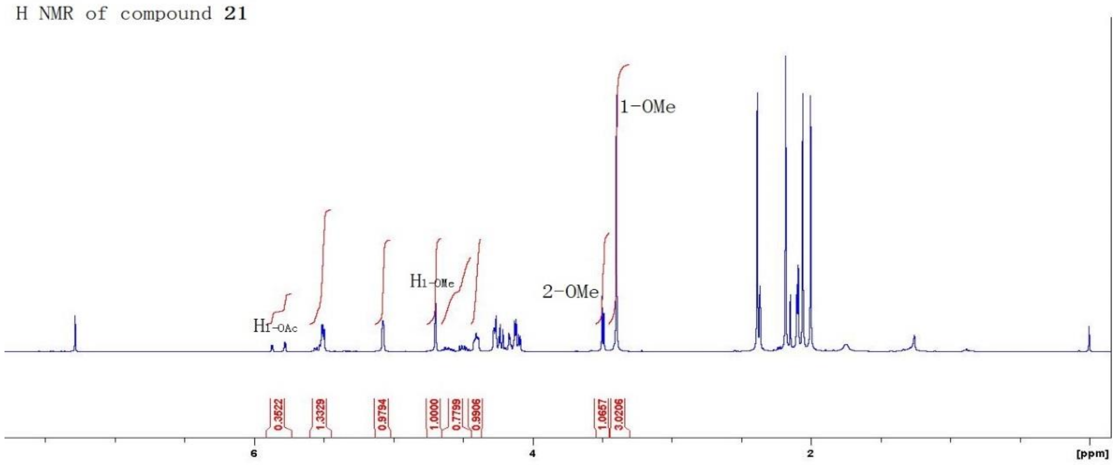

The side-products of compound **21** could not be isolated and should be also caused by 1-OMe participation. The possible structure should be:

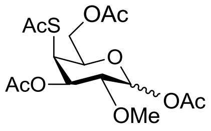

4. Preparation and characterization for the products

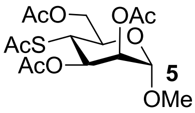

5

Methyl 4-*S*-acetyl-2, 3, 6-tri-*O*-acetyl-α-D-mannopyranoside 5[1]: to a solution of methyl α-D-glucopyranoside **7** (100 mg, 0.2 mmol) in acetic anhydride (1 mL) was added KOAc (98 mg, 1.0 mmol), and then allowed to react at 75 °C for 24 h. The mixture was then concentrated and directly purified by flash column chromatography (4:1 hexane-ethyl acetate), yielding **5** as a colorless syrup (61 mg, 76%). ^{1}H NMR (400 MHz, $CDCl_3$): δ 5.24 (dd, *J* =

3.0 Hz, 11.2 Hz, 1H, H-3), 5.13 (dd, J = 1.7 Hz, 3.2 Hz, 1H, H-2), 4.72 (d, J = 1.7 Hz, 1H, H-1), 4.33 (dd, J = 5.3 Hz, 12.1 Hz, 1H, H-6_a), 4.17 (dd, J = 1.4 Hz, 12.1 Hz, 1H, H-6_b), 4.01–3.88 (m, 2H, H-5, H-4), 3.36 (s, 3H, OMe), 2.30 (s, 3H, SAc), 2.13 (s, 3H, OAc), 2.06 (s, 3H, OAc), 1.94 (s, 3H, OAc) ppm.

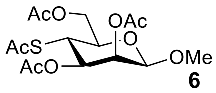

6

Methyl 4-*S*-acetyl-2, 3, 6-tri-O-acetyl -β-D-mannopyranoside 6: to a solution of methyl β-D-glucopyranoside **8** (100 mg, 0.2 mmol) in acetic anhydride (1 mL) was added KOAc (98 mg, 1.0 mmol), and then allowed to react at 75 °C for 24 h. The mixture was then concentrated and directly purified by flash column chromatography (4:1 hexane-ethyl acetate), yielding **6** as a colorless syrup (42.8 mg, 53%) and **6a** (31.5 mg, 39%). ^{1}H NMR (400 MHz, $CDCl_3$): δ 5.43 (d, J = 3.0 Hz, 1H, H-2), 5.10 (dd, J = 11.3 Hz, 3.0, Hz 1H, H-3), 4.51 (s, 1H, H-1), 4.45 – 4.35 (m, 1H, H-4), 4.29 (d, J = 11.5 Hz, 1H, H-5), 3.87 – 3.80 (m, 2H, H-6a, H-6_b), 3.54 (s, 3H, OMe), 2.33 (s, 3H, SAc), 2.19 (s, 3H, OAc), 2.06 (s, 3H, OAc), 1.99 (s, 3H, OAc) ppm. ^{13}C NMR (100 MHz, $CDCl_3$): δ 193.1, 170.9, 170.3, 169.9, 98.8, 69.9, 68.74 67.6, 63.9, 55.4, 40.7, 30.9, 21.1, 20.9, 20.8. HRMS (ESI-TOF) m/z: $[M+Na]^+$ Calcd for $C_{15}H_{22}O_9SNa$ 401.0883, found 401.0857.

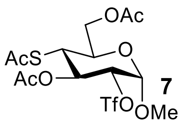

7

Methyl 4-*S*-acetyl-3,6-di-O-acetyl-2-O-triflyl-α-D-glucopyranoside 7: to a solution of methyl α-D-galactopyranoside **1** (280 mg, 1.0 mmol) in CH_2Cl_2 (10 mL) was added pyridine (1.06 mL) at -20 °C. Trifluoromethanesulfonic anhydride (1.02 mL, 5 mmol) in CH_2Cl_2 (4 mL) was added dropwise, and the mixture was stirred while allowing to warm from -20 °C to 10 °C over 2 h. The resulting mixture was subsequently diluted with CH_2Cl_2 and washed with 1 M HCl, aqueous $NaHCO_3$, water, and brine. The organic phase was dried over $MgSO_4$ and concentrated in vacuo at low temperature. The residue was used directly in the next step without further purification. KSAc (125 mg ,1.1 mmol) was added to a solution of the protected triflate residue in dry acetonitrile（4 mL）and then allowed to react at room temperature for 0.5 h. The mixture was the concentrated and directly purified by flash column chromatography (4:1 hexane-ethyl acetate), yielding **7** as a colorless syrup (409 mg, 88%). ^{1}H NMR (400 MHz, $CDCl_3$) δ 5.54 (dd, J = 9.7 Hz, 11.1 Hz, 1H, H-3), 5.02 (d, J = 3.6 Hz, 1H, H-1), 4.79 (dd, J = 3.6 Hz, 9.7 Hz, 1H, H-2), 4.36 (dd, J = 4.8 Hz, 12.3 Hz, 1H, H-6), 4.11 (dd, J = 2.0 Hz, 12.3 Hz, 1H, H-6’), 4.03 (ddd, J = 2.0 Hz, 4.8 Hz, 11.3 Hz, 1H, H-5), 3.76 (t, J = 11.3 Hz, 1H, H-4), 3.45 (s, 3H, OMe), 2.32 (s, 3H, SAc), 2.07 (s, 3H, OAc), 2.03 (s, 3H, OAc) ppm. ^{13}C NMR (100 MHz, $CDCl_3$) δ 192.4, 170.8, 169.6, 123.3, 120.1, 117.0, 113.8, 97.2, 82.7, 68.8, 67.2, 62.8, 56.2, 44.2, 30.8, 20.9, 20.5. HRMS (ESI-TOF) m/z: $[M + Na]^+$ Calcd for $C_{14}H_{19}F_3O_{10}S_2Na$ 491.0269; found, 491.0261.

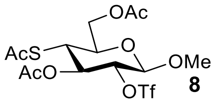

8

Methyl 4-*S*-acetyl-3,6-di-O-acetyl-2-O-triflyl-β-D-glucopyranoside 8: to a solution of methyl β-D-galactopyranoside **2** (280 mg, 1.0 mmol) in CH_2Cl_2 (5 mL) was added pyridine (1.06 mL) at -20 °C. Trifluoromethanesulfonic anhydride (1.02 mL, 5 mmol) in CH_2Cl_2 (4 mL) was added dropwise, and the mixture was stirred while allowing to warm from -20 °C to 10 °C over 2 h.The resulting mixture was subsequently diluted with CH_2Cl_2 and washed with 1 M HCl, aqueous $NaHCO_3$, water, and brine. The organic phase was dried over $MgSO_4$ and concentrated in vacuo at low temperature. The residue was used directly in the next step without

further purification. KSAc (125.4 mg, 1.1 mmol) was added to a solution of the protected triflate residue in dry acetonitrile（4 mL）and then allowed to react at room temperature for 0.5 h. The mixture was the concentrated and directly purified by flash column chromatography (4:1 hexane-ethyl acetate), yielding **8** as a colorless syrup (413.4 mg, 89%). ^{1}H NMR (400 MHz, $CDCl_3$) δ 5.42 (dd, *J* = 9.8Hz, 10.8 Hz, 1H, H-3), 4.62 (dd, *J* = 8.0 Hz, 9.8 Hz, 1H, H-2), 4.49 (d, *J* = 8.0 Hz, 1H, H-1), 4.34 (dd, *J* = 4.7 Hz, 12.3 Hz, 1H, H-6), 4.23 (dd, *J* = 1.9 Hz, 12.3 Hz, 1H, H-6'), 3.90 (m, 1H, H-5), 3.61 (t, *J* = 10.8 Hz, 1H, H-4), 3.56 (s, 3H, OMe), 2.31 (s, 3H, SAc), 2.07 (s, 3H, OAc), 2.06 (s, 3H, OAc) ppm. ^{13}C NMR (100 MHz, $CDCl_3$) δ 192.7, 170.8, 169.8, 123.2, 120.1, 116.9, 113.8, 100.7, 83.7, 72.9, 69.6, 62.9, 57.8, 44.8, 30.9, 20.9, 20.6. HRMS (ESI-TOF) m/z: $[M+Na]^+$ Calcd for $C_{14}H_{19}O_{10}S_2F_3Na$ 491.0269, found 491.0260.

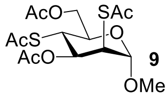

9

Methyl 2,4-di-*S*-acetyl-3,6-di-O-acetyl-a-D-mannopyranoside 9[1]: to a solution of methyl α-D-galactopyranoside **1** (140 mg, 0.5 mmol) in CH_2Cl_2 (5 mL) was added pyridine (0.53 mL) at -20 °C. Trifluoromethanesulfonic anhydride (0.51 mL, 5 mmol) in CH_2Cl_2 (2 mL) was added dropwise, and the mixture was stirred while allowing to warm from -20 °C to 10 °C over 2 h.The resulting mixture was subsequently diluted with CH_2Cl_2 and washed with 1 M HCl, aqueous $NaHCO_3$, and brine. The organic phase was dried over $MgSO_4$ and concentrated in vacuo at low temperature. The residue was used directly in the next step without further purification. KSAc (285 mg, 2.5 mmol) and HSAc (56μL, 0.75 mmol) was added to a solution of the protected triflate residue in dry acetonitrile (4 mL) and then allowed to react at 50 °C for 24 h. The resulting mixture was directly purified by flash column chromatography (4:1 hexane-ethyl acetate), yielding **9** as a colorless syrup (148 mg, 76%). ^{1}H NMR (400 MHz, $CDCl_3$): δ 5.52 (dd, *J* = 4.4 Hz, 11.5 Hz, 1H, H-3), 4.83 (d, *J* = 1.4 Hz, 1H, H-1), 4.34 (dd, *J* = 5.2 Hz, 12.1Hz,1H, H-6_a), 4.25 (dd, *J* = 1.4 Hz, 4.4 Hz, 1H, H-2), 4.18 (dd, *J* = 2.1 Hz,12.1, 1H, H-6_b), 3.99 (ddd, *J* = 2.1 Hz, 5.2 Hz, 11.2 Hz,1H, H-5), 3.81 (t, *J* =11.2 Hz, 1H, H-4), 3.40 (S, 3H,OMe), 2.39 (s, 3H, SAc), 2.35 (s, 3H, SAc), 2.13 (s, 3H, OAc),1.97 (s, 3H, OAc) ppm.

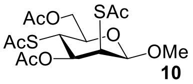

10

Methyl 2,4-di-*S*-acetyl-3,6-di-O-acetyl-β-D-mannopyranoside 10[1]: to a solution of methyl β-D-galactopyranoside **2** (140 mg, 0.5 mmol) in CH_2Cl_2 (5 mL) was added pyridine (0.53 mL) at -20 °C. Trifluoromethanesulfonic anhydride (0.51 mL, 5 mmol) in CH_2Cl_2 (2 mL) was added dropwise, and the mixture was stirred while allowing to warm from -20 °C to 10 °C over 2 h. The resulting mixture was subsequently diluted with CH_2Cl_2 and washed with 1 M HCl, aqueous $NaHCO_3$, water, and brine. The organic phase was dried over $MgSO_4$ and concentrated in vacuo at low temperature. The residue was used directly in the next step without further purification. KSAc (285 mg, 2.5 mmol) and HSAc (56μL, 0.75 mmol) was added to a solution of the protected triflate residue in dry acetonitrile（4 mL）and then allowed to react at 50 °C for 12 h. The mixture was directly purified by flash column chromatography (4:1 hexane-ethyl acetate), yielding **10** as a colorless syrup (179 mg, 91%). ^{1}H NMR (400 MHz, $CDCl_3$): δ 5.22 (dd, *J* = 4.1 Hz, 10.8 Hz,1H, H-3), 4.62 (d, *J* = 1.6 Hz, 1H, H-1), 4.36 (dd, *J* = 1.6 Hz, 4.1 Hz,1H, H-2), 4.30 (dd, *J* = 12.1 Hz, 4.9 Hz, 1H, H-6_a), 4.21 (dd, *J* = 2.1 Hz,12.1 Hz, 1H, H-6_b), 3.76 (ddd, *J* = 2.1 Hz, 4.9 Hz, 10.8 Hz,1H, H-5), 3.68 (t, *J* = 10.8 Hz, 1H, H-4), 3.49 (S, 3H, OMe), 2.36 (s, 3H, SAc), 2.31 (s, 3H, SAc), 2.08 (s, 3H, OAc),1.94 (s, 3H, OAc) ppm.

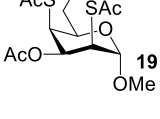

19

Methyl 2,4-di-*S*-acetyl-3,6-di-*O*-acetyl-α-D-talopyranoside 19: to a solution of methyl α-D-galactopyranoside **23** (100 mg, 0.2 mmol) in DMF (1 mL) was added KSAc (73 mg, 3.0 equiv), and the mixture was stirred at 50 °C for 24 h. The resulting mixture was subsequently diluted with ethyl acetate and washed with 1 M HCl, aqueous $NaHCO_3$, water, and brine. The organic phase was concentrated and directly purified by flash column chromatography (4:1 hexane-ethyl acetate), yielding **19** as a colorless syrup (46 mg, 54%) and **19a** as a colorless syrup (28 mg, 40%). ^{1}H NMR (400 MHz, $CDCl_3$) δ 5.77 (t, *J* = 5.0 Hz, 1H, H-3), 4.64 (s, 1H, H-1), 4.46–4.34 (m, 1H, H-5), 4.25–4.13 (m, 2H, H-4, H-6), 4.09–3.96 (m, 2H, H-6', H-2), 3.34 (s, 3H, OMe), 2.37 (s, 3H, SAc), 2.34 (s, 3H, SAc), 2.00 (s, 3H, OAc), 1.95 (s, 3H, OAc) ppm. ^{13}C NMR (100 MHz, $CDCl_3$) δ 194.2, 193.7, 170.7, 169.5, 102.5, 67.1, 65.8, 64.2, 55.4, 45.4, 44.5, 31.0, 30.9, 20.9, 20.8. HRMS (ESI-TOF) m/z: $[M+Na]^+$ Calcd for $C_{15}H_{22}O_8S_2Na$ 417.0653, found 417.0672.

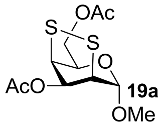

19a: ^{1}H NMR (400 MHz, Chloroform-*d*) δ 5.94 (s, 1H, H-3), 4.70 (s, 1H, H-1), 4.25 (dd, *J* = 10.3 Hz, 5.4 Hz, 1H, H-6), 4.20–4.09 (m, 2H, H-6', H-5), 3.95 (s, 1H, H-4), 3.85 (s, 1H, H-2), 3.40 (s, 3H, OMe), 2.13 (s, 3H, SAc), 2.07 (s, 3H, SAc) ppm. ^{13}C NMR (100 MHz, $CDCl_3$) δ 170.4, 170.1, 103.1, 75.8, 69.9, 63.6, 55.2, 55.0, 54.3, 21.1, 20.8. HRMS (ESI-TOF) m/z: $[M+Na]^+$ Calcd for $C_{11}H_{16}O_6S_2Na$ 331.0286, found 331.0287.

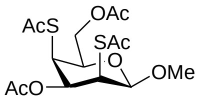

20

Methyl 2,4-di-*S*-acetyl-3,6-di-*O*-acetyl-β-D-talopyranoside 20: to a solution of methyl α-D-galactopyranoside **24** (100 mg, 0.2 mmol) in DMF (1 mL) was added KSAc (73 mg, 0.6 mmol), and the mixture was stirred at 50 °C for 48 h. The resulting mixture was subsequently diluted with ethyl acetate and washed with 1 M HCl, aqueous $NaHCO_3$, water, and brine. The organic phase was concentrated and directly purified by flash column chromatography (4:1 hexane-ethyl acetate), yielding **20** as a colorless syrup (48 mg, 57%). ^{1}H NMR (400 MHz, $CDCl_3$) δ 5.49 (t, *J* = 4.5 Hz, 1H, H-3), 4.61 (d, *J* = 2.6 Hz, 1H, H-1), 4.37–4.25 (m, 2H, H-6a, H-2), 4.22–4.18 (m, 1H, H-4), 4.15 (dd, *J* = 4.9 Hz, 11.6 Hz, 1H, H-6b), 4.05 (ddd, *J* = 3.2 Hz, 4.9 Hz, 7.8 Hz, 1H, H-5), 3.47 (s, 3H, OMe), 2.36 (s, 3H, SAc), 2.35 (s, 3H, SAc), 2.02 (s, 3H, OAc), 1.99 (s, 3H, OAc) ppm. ^{13}C NMR (100 MHz, $CDCl_3$) δ 194.1, 193.7, 170.8, 169.8, 100.9, 77.6, 77.2, 76.9, 72.7, 68.3, 64.1, 57.2, 46.8, 43.8, 30.9, 30.8, 21.0, 20.9. HRMS (ESI-TOF) m/z: $[M+Na]^+$ Calcd for $C_{15}H_{22}O_8S_2Na$ 417.0653, found 417.0673.

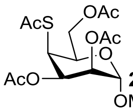

Methyl 4-*S*-acetyl-2, 3, 6-tri-*O*-acetyl-α-D-talapyranoside 21: to a solution of methyl α-D-galactopyranoside **23** (100 mg, 0.2 mmol) in acetic anhydride (1 mL) was added KOAc (98 mg, 1.0 mmol), and then allowed to react at 75 °C for 24 h. The mixture was then concentrated and directly purified by flash column chromatography (4:1 hexane-ethyl acetate), yielding a mixture of **21** with side-products as a colorless syrup (63 mg, 78%, **21**, 58%). ^{1}H NMR (400 MHz, $CDCl_3$) δ 5.51 (td, *J* = 5.5 Hz, 2.8 Hz, 1H, H-3), 5.07 (dd, *J* = 2.8 Hz, 1.7 Hz, 1H, H-2), 4.70 (d, *J* = 1.7 Hz, 1H, H-1), 4.40 (ddd, *J* = 7.3 Hz, 4.5 Hz, 2.2 Hz, 1H, H-5), 4.30 – 4.23 (m, 1H, H-4), 4.17 – 4.05 (m, 2H, H-6a, H-6b), 3.40 (s, 3H, OMe), 2.38 (s, 3H, SAc), 2.18 (s, 3H, OAc), 2.06 (s, 3H, OAc), 2.00 (s, 3H, OAc) ppm.

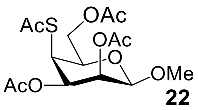

22

Methyl 4-*S*-acetyl-2, 3, 6-tri-*O*-acetyl-β-D-talapyranoside 22: to a solution of methyl β-D-galactopyranoside **24** (100 mg, 0.2 mmol) in acetic anhydride (1 mL) was added KOAc (98 mg, 1.0 mmol), and then allowed to react at 75 °C for 24 h. The mixture was then concentrated and directly purified by flash column chromatography (4:1 hexane-ethyl acetate), yielding **22** as a colorless syrup (43.6 mg, 54%). ^{1}H NMR (400 MHz, $CDCl_3$): δ 5.37 (dt, *J* = 3.0 Hz, 1.4 Hz, 1H, H-2), 5.28 (dd, *J* = 5.4 Hz, 3.0 Hz, 1H, H-3), 4.51 (d, *J* = 1.4 Hz, 1H, H-1), 4.30 (dd, *J* = 11.5 Hz, 7.1 Hz, 1H, H-6a), 4.26 (dd, *J* = 5.4 Hz, 2.1 Hz, 1H, H-4), 4.22 (dd, *J* = 11.5 Hz, 5.4 Hz, 1H, H-6b), 4.06 (ddd, *J* = 7.1 Hz, 5.4 Hz, 2.1 Hz, 1H, H-5), 3.55 (s, 3H, OMe), 2.40 (s, 3H, SAc), 2.22 (s, 3H, OAc), 2.06 (s, 3H, OAc), 2.01 (s, 3H, OAc) ppm.^{13}C NMR (100 MHz, $CDCl_3$): δ 193.2, 170.8, 170.4, 169.8, 99.6 73.2, 69.5, 68.1, 63.6, 63.5, 57.3, 40.6, 30.8, 20.8, 20.5. HRMS (ESI-TOF) m/z: $[M + Na]^+$ Calcd for $C_{15}H_{22}O_9SNa$ 401.0882, found 401.0861.

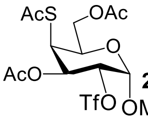

23

Methyl 4-*S*-acetyl-3,6-di-O-acetyl-2-O-triflyl-α-D-galactopyranoside 23: to a solution of methyl α-D-glucopyranoside **13** (140 mg, 0.5 mmol) in CH_2Cl_2 (5 mL) was added pyridine (0.53 mL) at -20 °C. Trifluoromethanesulfonic anhydride (0.51 mL,5 mmol) in CH_2Cl_2 (2 mL) was added dropwise, and the mixture was stirred while allowing to warm from -20 °C to 10 °C over 2 h. The resulting mixture was subsequently diluted with CH_2Cl_2 and washed with 1 M HCl, aqueous $NaHCO_3$, and brine. The organic phase was dried over $MgSO_4$ and concentrated in vacuo at low temperature. The residue was used directly in the next step without further purification. TBASAc (160 mg, 1.1 mmol) was added to a solution of the protected triflate residue in dry toluene （2 mL） and then allowed to react at room temperature for 1 h. The mixture was the concentrated and directly purified by flash column chromatography (4:1 hexane-ethyl acetate), yielding **23** as a colorless syrup (182 mg, 77%). ^{1}H NMR (400 MHz, $CDCl_3$) δ 5.55 (dd, *J* = 4.5 Hz, 10.5 Hz, 1H, H-3), 4.95 (d, *J* = 3.8 Hz, 1H, H-1), 4.79 (dd, *J* = 3.8 Hz, 10.5 Hz, 1H, H-2), 4.45 (ddd, *J* = 1.7 Hz, 5.1 Hz, 7.1 Hz, 1H, H-5), 4.36 (dd, *J* = 1.7 Hz, 4.5 Hz, 1H, H-4), 4.19 (dd, *J* = 7.1 Hz, 11.6 Hz, 1H, H-6a), 4.04 (dd, *J* = 5.1 Hz, 11.6 Hz, 1H, H-6b), 3.44 (s, 3H, OMe), 2.38 (s, 3H, SAc), 2.03 (s, 3H, OAc), 1.97 (s, 3H, OAc) ppm. ^{13}C NMR (100 MHz, $CDCl_3$) δ 192.9, 170.3, 169.3, 123.1, 119.9, 116.7, 113.6, 97.1, 80.7, 66.9, 66.8, 63.3, 55.8, 47.2, 30.8, 20.6, 20.3. HRMS (ESI-TOF) m/z: $[M+Na]^+$ Calcd for $C_{14}H_{19}O_{10}S_2F_3Na$ 491.0269, found 491.0255.

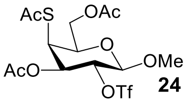

24

Methyl 4-*S*-acetyl-3,6-di-*O*-acetyl-2-*O*-triflyl-β-D-galactopyranoside 24: to a solution of methyl α-D-glucopyranoside **14** (140 mg, 0.5 mmol) in CH_2Cl_2 (5 mL) was added pyridine (0.53 mL) at -20°C. Trifluoromethanesulfonic anhydride (0.51 mL, 5 mmol) in CH_2Cl_2 (2 mL) was added dropwise, and the mixture was stirred while allowing to warm from -20 °C to 10 °C over 2 h. The resulting mixture was then diluted with CH_2Cl_2 and washed with 1 M HCl, aqueous $NaHCO_3$, and brine. The organic phase was dried and concentrated in vacuo at low temperature. The residue was used directly in the next step without further purification. TBASAc (160 mg ,1.1 mmol) was added to a solution of the protected triflate residue in dry toluene（2 mL）and then allowed to react at room temperature for 1 h. The mixture was then concentrated and directly purified by flash column chromatography (4:1 hexane-ethyl acetate), yielding **24** as a colorless syrup (191 mg, 81%). ^{1}H NMR (400 MHz, $CDCl_3$) δ 5.28 (dd, *J* = 4.6 Hz, 10.2 Hz, 1H, H-3), 4.60 (dd, *J* = 7.7 Hz, 10.2 Hz, 1H, H-2), 4.46 (d, *J* = 7.7 Hz, 1H,

H-1), 4.33 (dd, *J* = 1.2 Hz, 4.6 Hz, 1H, H-4), 4.25 (dd, *J* = 6.3 Hz, 11.0 Hz, 1H, H-6a), 4.16–4.02 (m, 2H, H-6b, H-5), 3.54 (s, 3H, OMe), 2.38 (s, 3H, SAc), 2.03 (s, 3H, OAc), 1.99 (s, 3H, OAc) ppm. ^{13}C NMR (100 MHz, $CDCl_3$) δ 193.2, 170.6, 169.7, 123.4, 120.2, 117.0, 113.8, 101.5, 82.4, 77.6, 77.2, 76.9, 71.7, 70.2, 63.0, 57.8, 46.7, 30.9, 20.8, 20.5. HRMS (ESI-TOF) m/z: $[M+Na]^+$ Calcd for $C_{14}H_{19}O_{10}S_2F_3Na$ 491.0269, found 491.0253.

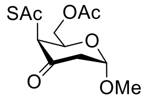

28

28: ^{1}H NMR (400 MHz, $CDCl_3$) δ 5.12 (d, *J* = 4 Hz, 1H, H-1), 4.35 (m, 2H, H-6), 4.17 (d, J = 12 Hz, 1H, H-4), 4.08 (dd, J = 12Hz, 4Hz, 1H, H-5), 3.31 (s, OMe, 3H), 2.83 (dd, J = 16 Hz, 4Hz, 1H, H-2a), 2.68 (d, J = 16Hz, 1H, H-2b), 2.33 (s, 3H, SAc), 2.06 (s, 3H, OAc) ppm. ^{13}C NMR (100 MHz, $CDCl_3$) δ = 198.0, 192.8, 170.7, 99.2, 70.3, 63.9, 55.1, 51.8, 46.5, 30.4, 20.8 ppm. HRMS (ESI-TOF) m/z: $[M+Na]^+$ Calcd for $C_{11}H_{16}O_6SNa$ 299.0565; found 299.0574.

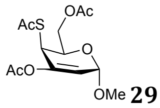

29

29: ^{1}H NMR (400 MHz, $CDCl_3$) δ 5.59 (d, *J* = 3.1 Hz, 1H, H-2), 5.03 (d, *J* = 3.1 Hz, 1H, H-1), 4.61 (ddd, *J* = 2.6 Hz, 4.8 Hz, 7.5 Hz, 1H, H-5), 4.28 (d, *J* = 2.6 Hz, 1H, H-4), 4.21 (dd, *J* = 7.7 Hz, 11.5 Hz, 1H, H-6a), 4.09 (dd, *J* = 4.8 Hz, 11.5 Hz, 1H, H-6b), 3.39 (s, 3H, OMe), 2.34 (s, 3H, SAc), 2.09 (s, 3H, OAc), 2.02 (s, 3H, OAc) ppm. ^{13}C NMR (100 MHz, $CDCl_3$) δ =194.2, 170.7, 168.6, 148.5, 114.9, 95.9, 67.6, 63.8, 55.6, 41.6, 30.6, 21.0, 20.9. HRMS (ESI-TOF) m/z: $[M+Na]^+$ Calcd for $C_{13}H_{18}O_7SNa$ 341.0671; found 341.0689.

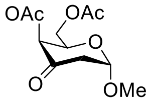

30

30: ^{1}H NMR (400 MHz, $CDCl_3$) δ 5.22 (1H, d, J = 12Hz, H-4), 5.16 (1H, d, J = 4Hz, H-1), 4.18-4.40 (3H, m), 3.38 (3H, m, OMe), 2.85 (1H, dd, 16Hz, 4Hz, H-2a), 2.67 (1H, d, 16 Hz, H-2b), 2.18 (3H, s, OAc), 2.13 (3H, s, OAc) ppm; ^{13}C NMR (100 MHz, $CDCl_3$) δ 197.6, 170.5, 169.3, 99.6, 72.9, 69.3, 62.6, 55.1, 46.0, 20.7, 20.4 ppm. HRMS (ESI-TOF) m/z: $[M+Na]^+$ Calcd for $C_{11}H_{16}O_7Na$ 283.0794; found 283.0808.

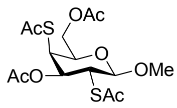

35

Methyl 2,4-di-*S*-acetyl-3,6-di-*O*-acetyl-β-D-galactopyranoside 35: to a solution of methyl β-D-mannopyranoside **33**, (140 mg, 0.5 mmol) in CH_2Cl_2 (5 mL) was added pyridine (0.53 mL) at -20 °C. Trifluoromethanesulfonic anhydride (0.51 mL,5 mmol) in CH_2Cl_2 (2 mL) was added dropwise, and the mixture was stirred while allowing to warm from -20 °C to 10 °C over 2 h. The resulting mixture was subsequently diluted with CH_2Cl_2 and washed with 1 M HCl, aqueous $NaHCO_3$, and brine. The organic phase was dried over $MgSO_4$ and concentrated in vacuo at low temperature. The residue was used directly in the next step without further purification. TBASAc (993 mg, 2.5 mmol) was added to a solution of the protected triflate residue in dry acetonitrile（2 mL）and then allowed to react at room temperature for 2 h. The mixture was then concentrated and directly purified by flash column chromatography (4:1 hexane-ethyl acetate), yielding **35** as a colorless syrup (166 mg, 84%). ^{1}H NMR (400 MHz, $CDCl_3$) δ 5.23 (dd, *J* = 4.2 Hz, 11.7 Hz, 1H, H-3), 4.44 (d, *J* = 8.9 Hz, 1H, H-1), 4.28-4.19 (m, 2H, H-4, H-6a), 4.11 (dd, *J* = 5.8 Hz, 11.4 Hz, 1H, H-6b), 4.05-3.97 (m, 1H, H-5), 3.53 (dd, *J* = 8.9 Hz, 11.7 Hz, 1H, H-2), 3.44 (s, 3H, OMe), 2.35 (s, 3H, SAc), 2.31 (s, 3H, SAc), 2.01 (s, 3H, OAc), 1.91 (s, 3H, OAc). ^{13}C NMR (100 MHz, $CDCl_3$) δ 193.9, 193.2, 170.6, 170.0, 102.7, 71.4, 69.9, 63.6, 57.3, 47.0, 46.9, 30.9, 30.9, 20.9, 20.8, HRMS (ESI-TOF) m/z: $[M + Na]^+$ Calcd for $C_{15}H_{22}O_8S_2Na$ 417.0654, found 417.0635.

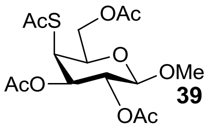

39

Methyl 4-*S*-acetyl-2,3,6-tri-*O*-acetyl-β-D-galactopyranoside 39[2]: to a solution of methyl β-D-mannopyranoside **33** (140 mg, 0.5 mmol) in CH_2Cl_2 (5 mL) was added pyridine (0.53 mL) at -20 ºC. Trifluoromethanesulfonic anhydride (0.51 mL, 5 mmol) in CH_2Cl_2 (2 mL) was added dropwise, and the mixture was stirred while allowing to warm from -20 ºC to 10 ºC over 2 h. The resulting mixture was subsequently diluted with CH_2Cl_2 and washed with 1 M HCl, aqueous $NaHCO_3$, water, and brine. The organic phase was dried over $MgSO_4$ and concentrated in vacuo at low temperature. The residue was used directly in the next step without further purification. TBAOAc (301 mg, 1.0 mmol) was added to a solution of the protected triflate residue in dry acetonitrile (2 mL) and then allowed to react at 0 ºC for 4 h. After TBASAc (476 mg, 1.5 mmol) was added, the mixture was stirred while allowing to warm from 0 ºC to 25 ºC over 3 h. The mixture was then concentrated and directly purified by flash column chromatography (4:1 hexane-ethyl acetate), yielding **39** as a colorless syrup (91 mg, 48%). ^{1}H NMR (400 MHz, $CDCl_3$) δ 5.16 (dd, *J* = 4.5 Hz, 10.3 Hz, 1H, H-3), 4.97 (dd, *J* = 7.8 Hz, 10.3 Hz, 1H, H-2), 4.34 (d, *J* = 7.8 Hz, 1H, H-1), 4.29-4.21 (m, 2H, H-4, H-6a), 4.12 (dd, *J* = 5.8 Hz, 11.4 Hz, 1H, H-6b), 4.06-4.00 (td, *J* = 1.6 Hz, 6.1 Hz, 1H, H-5), 3.45 (s, 3H, OMe), 2.35 (s, 3H, SAc), 2.03 (s, 3H, OAc), 2.02(s, 3H, OAc), 1.93 (s, 3H, OAc) ppm.

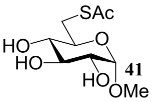

41

Methyl 6-*S*-acetyl-α-D-glucopyranoside 41[1]**:** to a solution of methyl 6-tosyl-α-D-glucopyranoside **40** (100 mg, 0.28 mmol) in DMF (2 mL) was added KSAc (48 mg, 0.42 mmol) and TsOH (24 mg, 0.14 mmol), and then allowed to react at 60 ºC for 6 h. The mixture was then concentrated and directly purified by flash column chromatography (ethyl acetate), yielding **41** as a colorless syrup (62 mg, 85%). ^{1}H NMR (400 MHz, $CDCl_3$): δ 4.69 (d, *J* = 3.4 Hz, 1H, H-1), 4.27 (s, 3H, OH), 3.75-3.57 (m, 2H, H-3, H-5), 3.52 (dd, *J* = 9.5 Hz, 3.4 Hz, 1H, H-2), 3.48-3.28 (m, 4H, H6a, OMe), 3.23 (dd, *J* = 16.9 Hz, 8.6 Hz, 1H, H-4), 3.08 (dd, *J* = 14.0 Hz, 7.5 Hz, 1H, H-6b), 2.38(s, 3H, SAc) ppm.

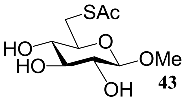

43

Methyl 6-*S*-acetyl-β-D-glucopyranoside 43: to a solution of methyl 6-tosyl-β-D-glucopyranoside **42** (100 mg, 0.28 mmol) in DMF (2 mL) was added KSAc (48 mg, 0.42 mmol) and TsOH (24 mg, 0.14 mmol), and then allowed to react at 60 ºC for 6 h. The mixture was then concentrated and directly purified by flash column chromatography (ethyl acetate), yielding **43** as a colorless syrup (55 mg, 76%). ^{1}H NMR (400 MHz, $CDCl_3$): δ 4.21 (d, *J* = 7.7 Hz, 1H, H-1), 4.00 (s, 3H, OH), 3.57 – 3.48 (m, 4H, H-4, OMe), 3.48 – 3.25 (m, 4H, H-6_a, H-6_b, H-3, H-2), 3.13 (dd, *J* = 14.3 Hz, 6.7 Hz, 1H, H-5), 2.38(s, 3H, SAc) ppm. ^{13}C NMR (100 MHz, $CDCl_3$): δ 197.6, 103.2, 75.8, 74.3, 73.5, 72.4, 57.0, 31.0, 30.5. HRMS (ESI-TOF) m/z: $[M + Na]^+$ Calcd for $C_9H_{16}O_6SNa$ 275.0565, found 275.0538.

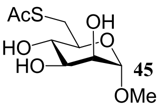

45

Methyl 6-*S*-acetyl-a-D-mannopyranside 45[1]: to a solution of methyl 6-tosyl-β-D-glucopyranoside **44** (100 mg, 0.28 mmol) in DMF (2 mL) was added KSAc (48 mg, 0.42 mmol) and TsOH (24 mg, 0.14 mmol), and then allowed to react at 60 ºC for 6 h. The mixture was then concentrated and directly purified by flash column

chromatography (ethyl acetate), yielding **45** as a colorless syrup (59 mg, 82%). ^{1}H NMR (400 MHz, $CDCl_3$): δ 4.68 (s, 1H, H-1), 3.94 (s, 1H, H-2), 3.80 (d, *J* = 7.3 Hz, 1H, H-3), 3.66 (dd, *J* = 7.3 Hz, 3.2 Hz, 1H, H-5), 3.57 (t, *J* = 7.3 Hz, 1H, H-4), 3.36 (s, 3H, OMe), 3.33 – 3.23 (m, 2H, H-6_a, H-6_b), 2.42 (s, 3H, SAc) ppm.

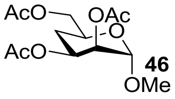

46

Methyl 2,3,6-tri-*O*-acetyl-4-deoxy-α-D-lyxo-hexopyranoside 46[3]: to a solution of methyl 4-*S*-acetyl-2,3, 6-tri-*O*-acetyl-α-D-mannopyranoside **5** (17.0 mg, 0.045 mmol) in DMF (0.5 mL) was added hydrated hydrazine (4.0 μL, 0.08 mmol) and stirred for 4 minutes. TCEP·HCl (32.0 mg, 0.11 mmol) and molecular sieve were added to the reaction and stirred for a few minutes. Then the reaction was exposed to UV light (a photochemistry reactor with a 500 W mercury lamp and a cooling circuit) at room temperature for 2 hours. The reaction mixture was concentrated in vacuo and directly purified by flash column chromatography (EtOAc/Petroleum ether = 1:3) to afford **46** as colorless oil (11.0 mg, 81% yield). ^{1}H NMR (400 MHz, $CDCl_3$) δ 5.26 (ddd, *J* = 11.5 Hz, 5.6 Hz, 3.2 Hz, 1H, H-3), 5.08 (dd, *J* = 3.2 Hz, 1.8 Hz, 1H, H-2), 4.73 (d, *J* = 1.8 Hz, 1H, H-1), 4.23 – 3.94 (m, 3H, H-5, H-6a, H-6b), 3.38 (s, 3H, OMe), 2.13 (s, 3H, OAc), 2.10 (s, 3H, OAc), 2.01 (s, 3H, OAc), 1.89 – 1.73 (m, 2H, H-4) ppm.

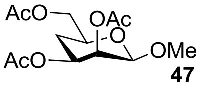

47

Methyl 2,3,6-tri-*O*-acetyl-4-deoxy-β-D-lyxo-hexopyranoside 47: to a solution of methyl 4-*S*-acetyl-2,3, 6-tri-*O*-acetyl-β-D-mannopyranoside **6** (22.0 mg, 0.06 mmol) in DMF (0.5 mL) was added hydrated hydrazine (5.0 μL, 0.1 mmol) and stirred for 4 minutes. Then TCEP·HCl (42.0 mg, 0.14 mmol) and molecular sieve were added to the reaction and stirred for a few minutes. Then the reaction was exposed to UV light (a photochemistry reactor with a 500 W mercury lamp and a cooling circuit) at room temperature for 2 hours. The reaction mixture was concentrated in vacuo and directly purified by flash column chromatography (EtOAc/Petroleum ether = 1:3) to afford **47** as colorless oil (22.0 mg, 79% yield). ^{1}H NMR (400 MHz, $CDCl_3$) δ 5.38 (dd, *J* = 3.2 Hz, 1.1 Hz, 1H, H-2), 5.06 – 4.92 (m, 1H, H-3), 4.43 (d, *J* = 1.1 Hz, 1H, H-1), 4.26 (dd, *J* = 11.6 Hz, 6.2 Hz, 1H, H-6a), 4.16 (dd, *J* = 11.6 Hz, 4.4 Hz, 1H, H-6b), 3.75 (m, 1H, H-5), 3.52 (s, 3H, OMe), 2.16 (s, 3H, OAc), 2.09 (s, 3H, OAc), 2.01 (s, 3H, OAc), 1.91 – 1.67 (m, 2H, H-4) ppm. ^{13}C NMR (100 MHz, $CDCl_3$): δ 170.73, 170.4, 170.0, 100.1, 69.9, 68.7, 67.4, 65.7, 57.2, 27.9, 20.9, 20.8, 20.8. HRMS (ESI-TOF) m/z: $[M + Na]^+$ Calcd for $C_{13}H_{20}O_8Na$ 327.1056, found 327.1059.

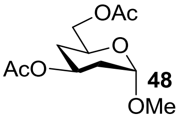

48

Methyl 3,6-Di-*O*-acetyl-2,4-di-deoxy-α-D-threo-hexopyranoside 48[4]: to a solution of methyl 2,4-di-*S*-acetyl-3,6-di-*O*-acetyl-2,4-thio-α-D-mannopyranoside **9** (61 mg, 0.15 mmol) in DMF (0.8 mL) was added hydrated hydrazine (19 μL, 0.4 mmol) and stirred for 4 minutes. Then TCEP·HCl (133.0 mg, 0.45 mmol) and molecular sieve were added to the reaction and stirred for a few minutes. Then the reaction was exposed to UV light (a photochemistry reactor with a 500 W mercury lamp and a cooling circuit) at room temperature for 2 hours. The reaction mixture was concentrated in vacuo and directly purified by flash column chromatography (EtOAc/Petroleum ether = 1:3) to afford **48** as colorless oil (31 mg, 80% yield). ^{1}H NMR (400 MHz, $CDCl_3$) δ 5.18 (tt, *J* = 11.5 Hz, 4.8 Hz, 1H, H-3), 4.89 (d, *J* = 3.4 Hz, 1H, H-1), 4.16 – 4.09 (m, 2H, H-6a, H-6b), 4.08 – 3.96 (m, 1H, H-5), 3.32 (s, 3H, OMe), 2.09 (m, 4H, H-4a, OAc), 2.03 (m, 4H, H-4b, OAc), 1.68 – 1.58 (m, 1H, H-2a), 1.40 (q, *J* = 11.5 Hz, 1H, H-2b) ppm.

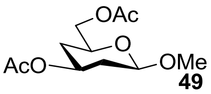

49

Methyl 3,6-Di-*O*-acetyl-2,4-di-deoxy-β-D-threo-hexopyranoside 49[4]**:** to a solution of methyl 2,4-di-*S*-acetyl-3,6-di-*O*-acetyl-2,4-thio-β-D-mannopyranoside **10** (20.0 mg, 0.05 mmol) in DMF (0.5 mL) was added hydrated hydrazine (6 μL, 0.13 mmol) and stirred for 4 minutes. Then TCEP·HCl (44.0 mg, 0.15 mmol) and molecular sieve were added to the reaction and stirred for a few minutes. Then the reaction was exposed to UV light (a photochemistry reactor with a 500 W mercury lamp and a cooling circuit) at room temperature for 2 hours. The reaction mixture was concentrated in vacuo and directly purified by flash column chromatography (EtOAc/Petroleum ether = 1:3) to afford **49** as colorless oil (13.0 mg, 84% yield). ^{1}H NMR (400 MHz, $CDCl_3$) δ 4.92 (tt, *J* = 11.5 Hz, 4.8 Hz, 1H, H-3), 4.37 (dd, *J* = 9.7 Hz, 2.1 Hz, 1H, H-1), 4.21 (dd, *J* = 11.5 Hz, 6.0 Hz, 1H, H-6a), 4.11 (dd, *J* = 11.5, 4.2 Hz, 1H, H-6b), 3.65 (dddd, *J* = 11.5 Hz, 6.0 Hz, 4.2 Hz, 2.0 Hz, 1H, H-5), 3.50 (s, 3H, OMe), 2.19 (ddt, *J* = 11.5 Hz, 4.2 Hz, 2.0 Hz, 1H, H-4a), 2.08 (s, 3H, OAc), 2.05 (s, 3H, OAc), 1.98 (ddd, *J* = 11.5 Hz, 4.8 Hz, 2.0 Hz, 1H, H-4b), 1.48 (td, *J* = 11.5 Hz, 9.7 Hz, 1H, H-2a), 1.36 (q, *J* = 11.5 Hz, 1H, H-2b) ppm.

References

1. Wu, B.; Ge, J.; Ren, B.; Pei, Z.; Dong, H. *Tetrahedron*, **2015**, 71, 4023−4030.
2. Yoshisuke, T.; Shinsuke, N.; Kimihiro, K.;Yoshiyuki, S.; Kyoko, K.; Yumiko, I.; et al. *Chem. Pharm. Bull.* **1997**, 45, 971-980.
3. Kaur, K. J.; Hindsgaul, O. *Carbohydr. Res*. **1992**, 226, 219-231.
4. Johnson, R. C.; Golebiowski, A.; Steensma, H. D.; Scialdone, A. M. *J. Org. Chem.***1993**, 58, 7185-7194.

5. NMR Spectra

Methyl 4-*S*-acetyl-2, 3, 6-tri-*O*-acetyl-4-thio-*α*-D-mannopyranoside 5

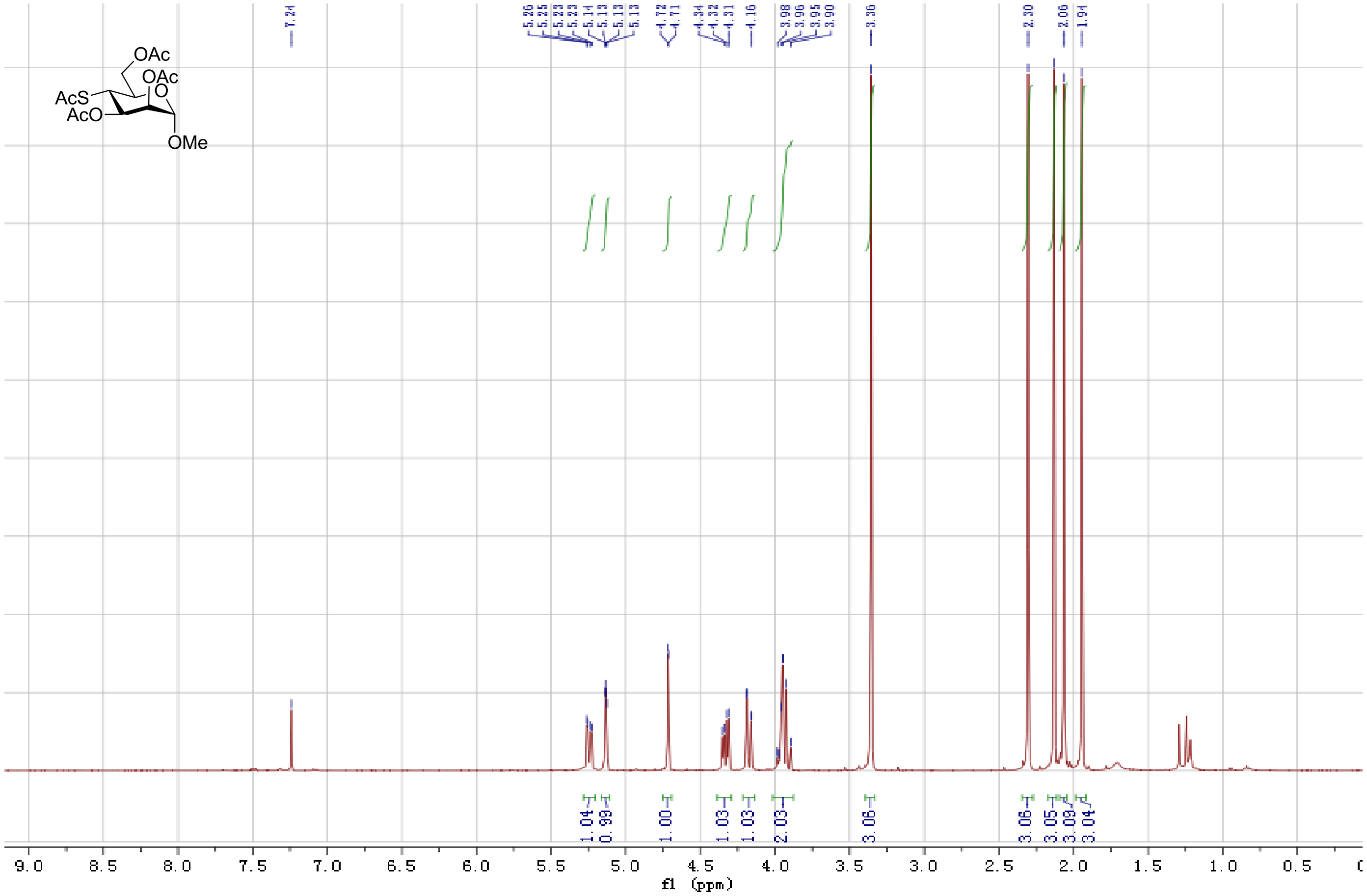

Figure S12. ^{1}H NMR spectrum (400 MHz) of **5** in $CDCl_3$

Methyl 4-*S*-acetyl-2, 3, 6-tri-*O*-acetyl-*β*-D-mannopyranoside 6

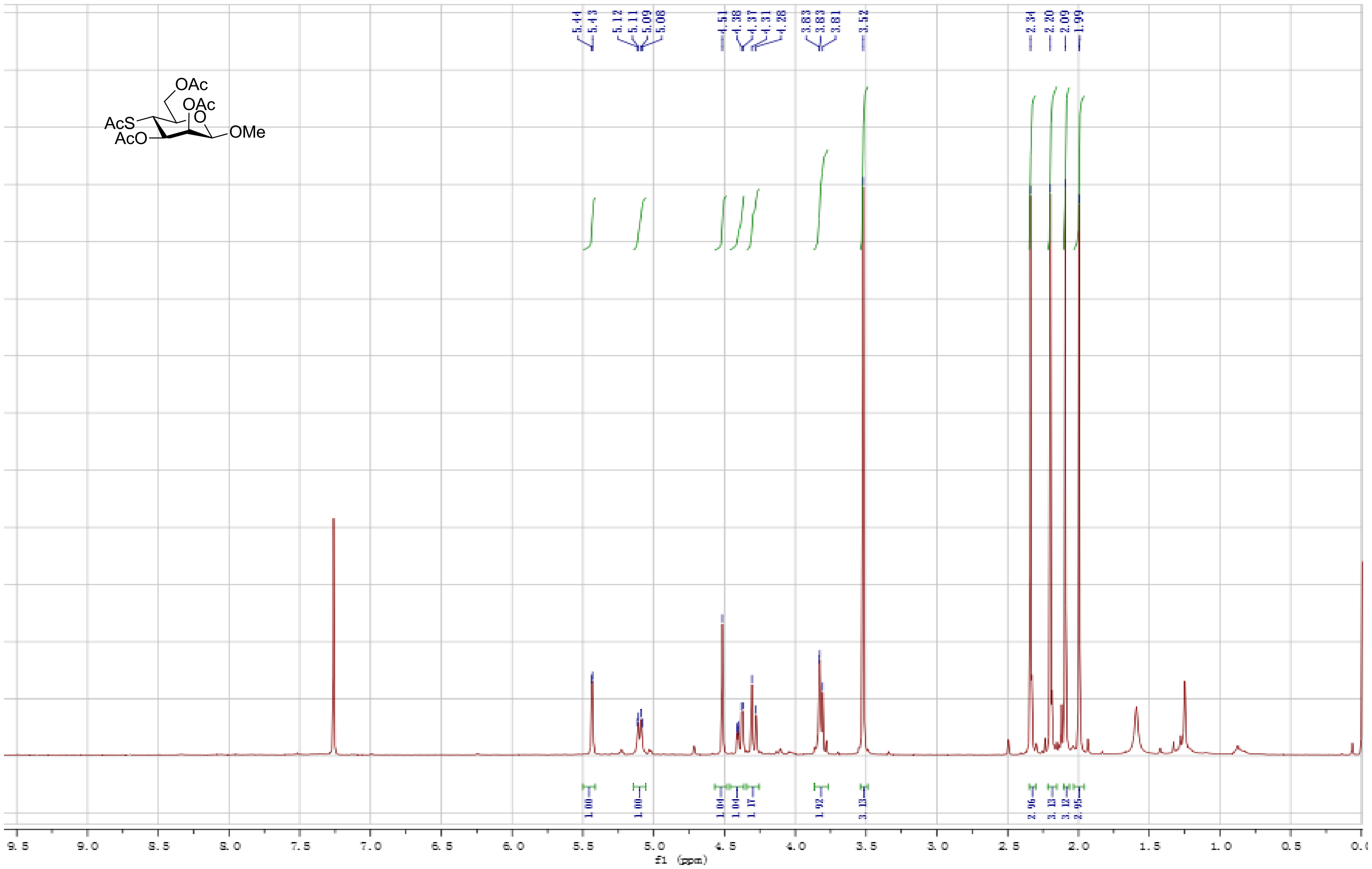

Figure S13. ^{1}H NMR spectrum (400 MHz) of **6** in $CDCl_3$

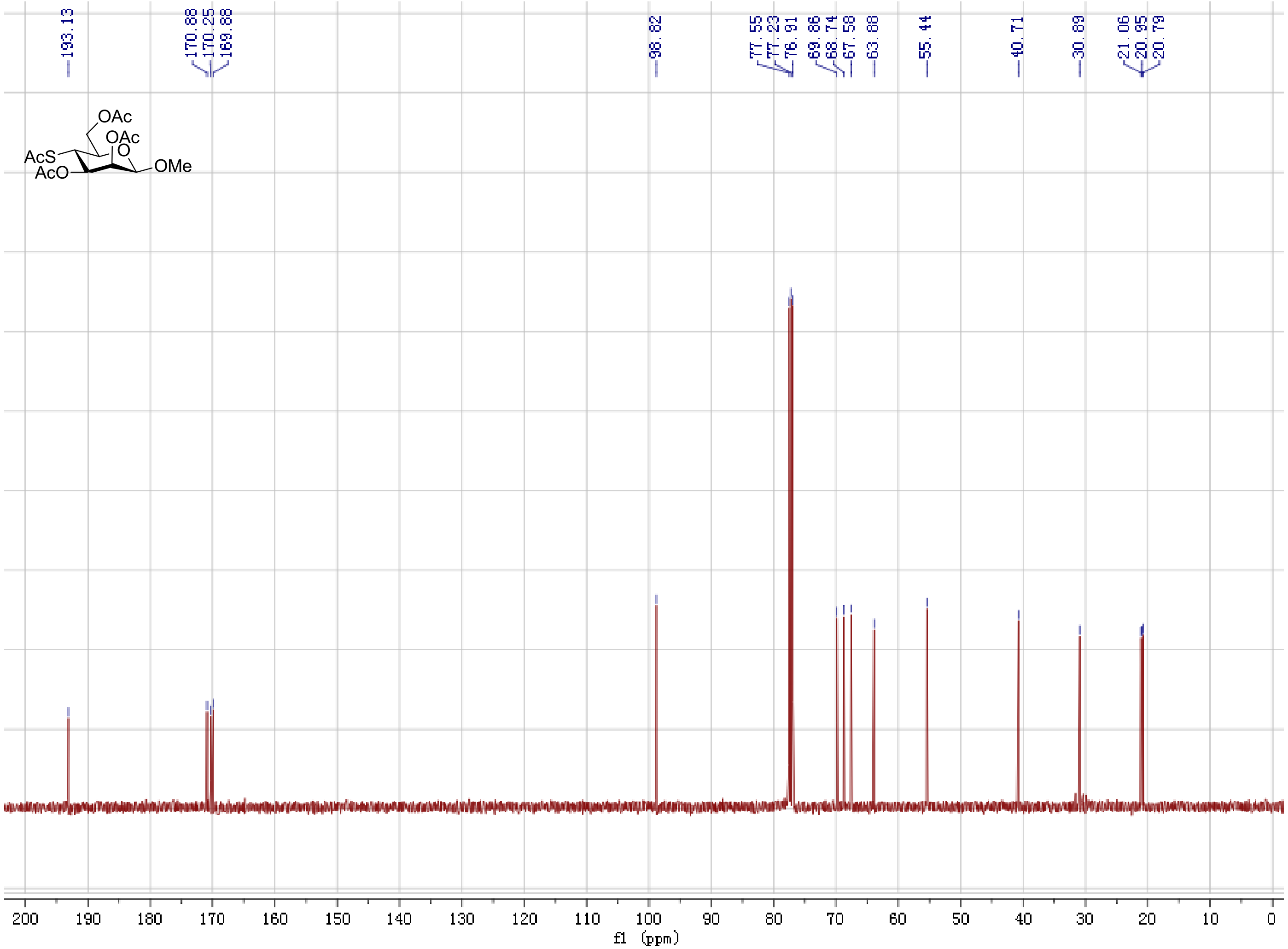

Figure S14. ^{13}C NMR spectrum (100 MHz) of **6** in $CDCl_3$

4-*S*-acetyl-1,3, 6-tri-*O*-acetyl-2-*O*-methyl-D-mannopyranoside 6a

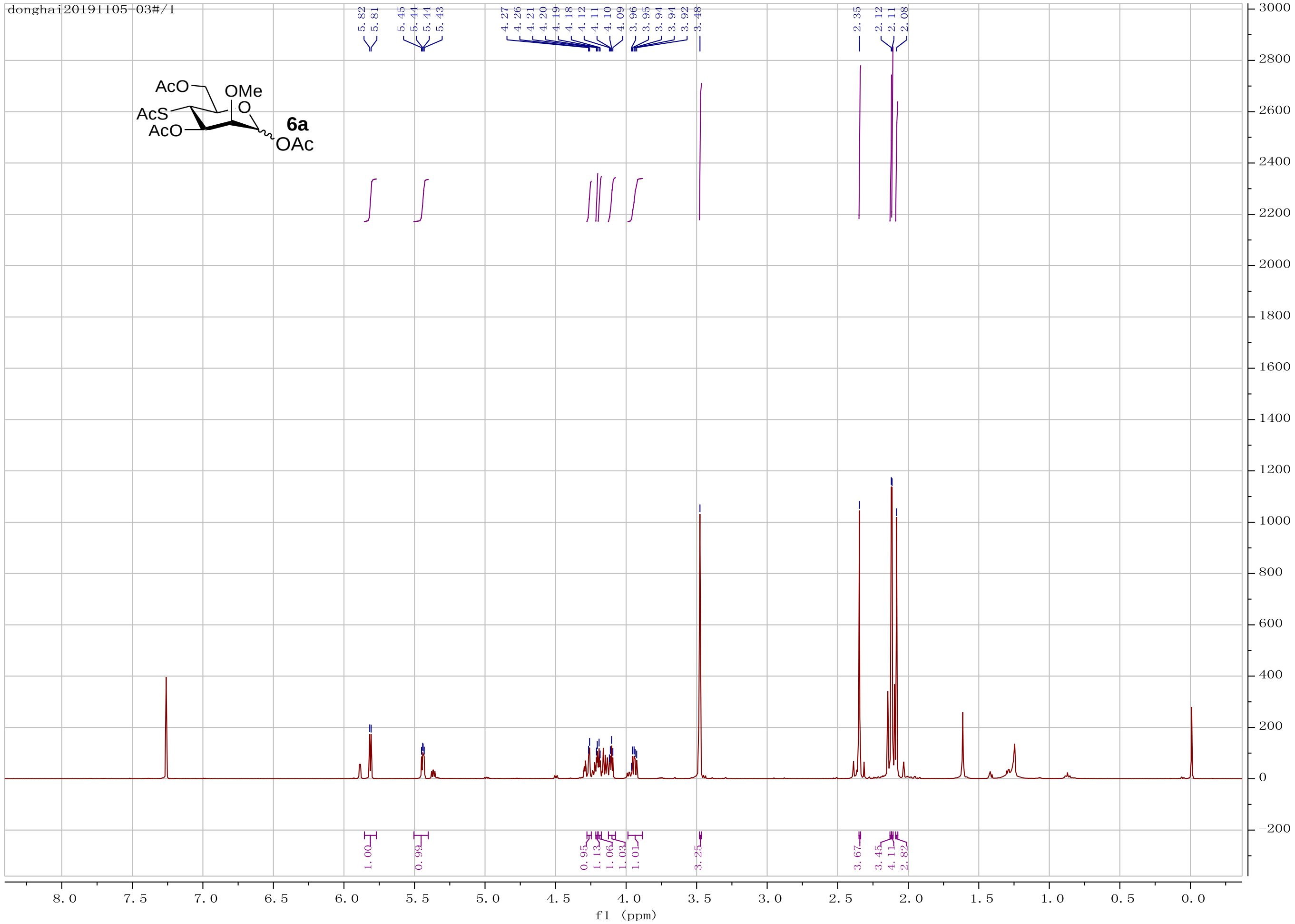

Figure S15. ^{1}H NMR spectrum (400 MHz) of **6a** in $CDCl_3$

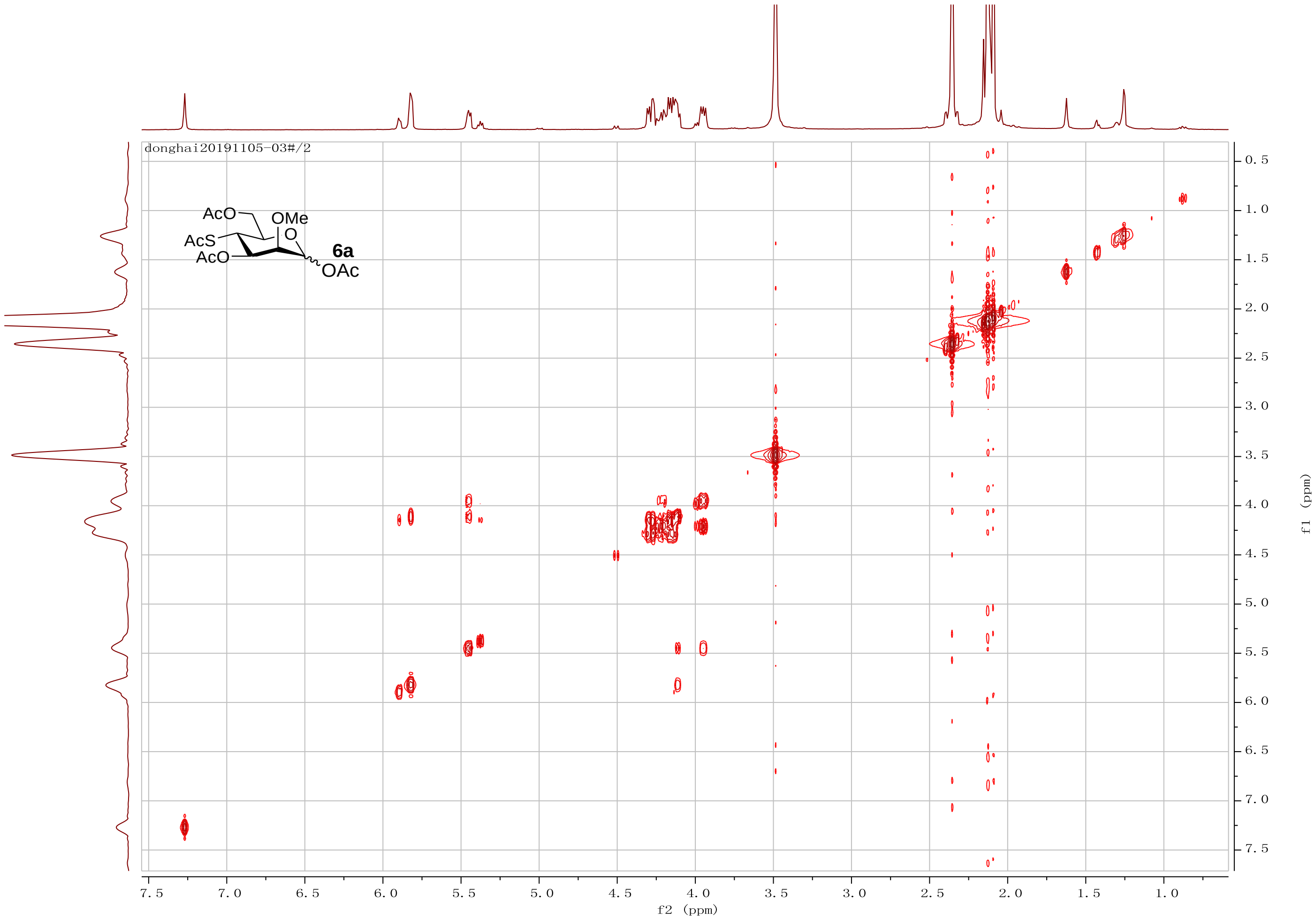

Figure S16. ^{1}H-^{1}H COSY spectrum of **6a** in $CDCl_3$

Methyl 4-*S*-acetyl-3,6-di-O-acetyl-2-*O*-triflyl-*α*-D-glucopyranoside 7

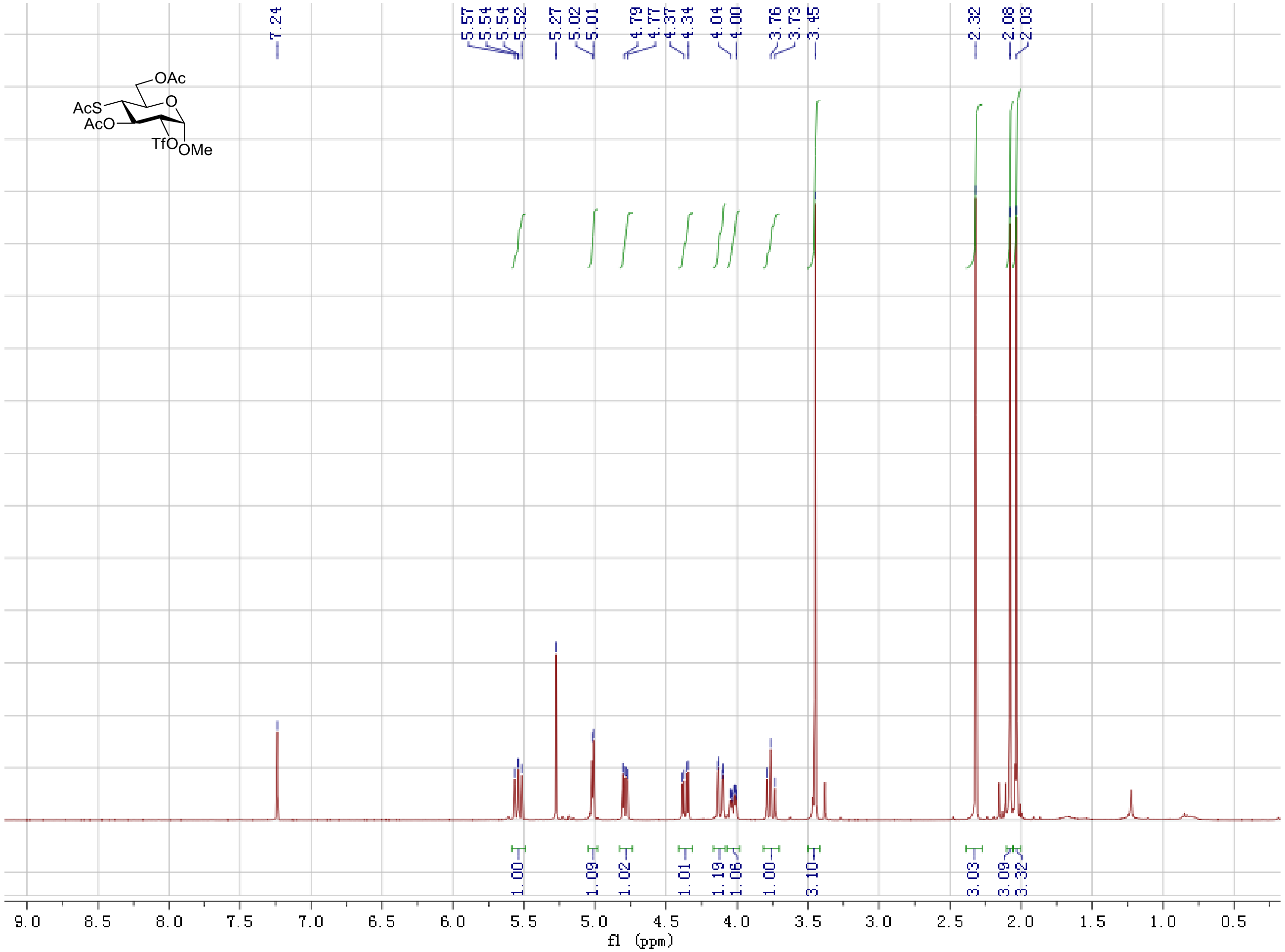

Figure S17. ^{1}H NMR spectrum (400 MHz) of **7** in $CDCl_3$

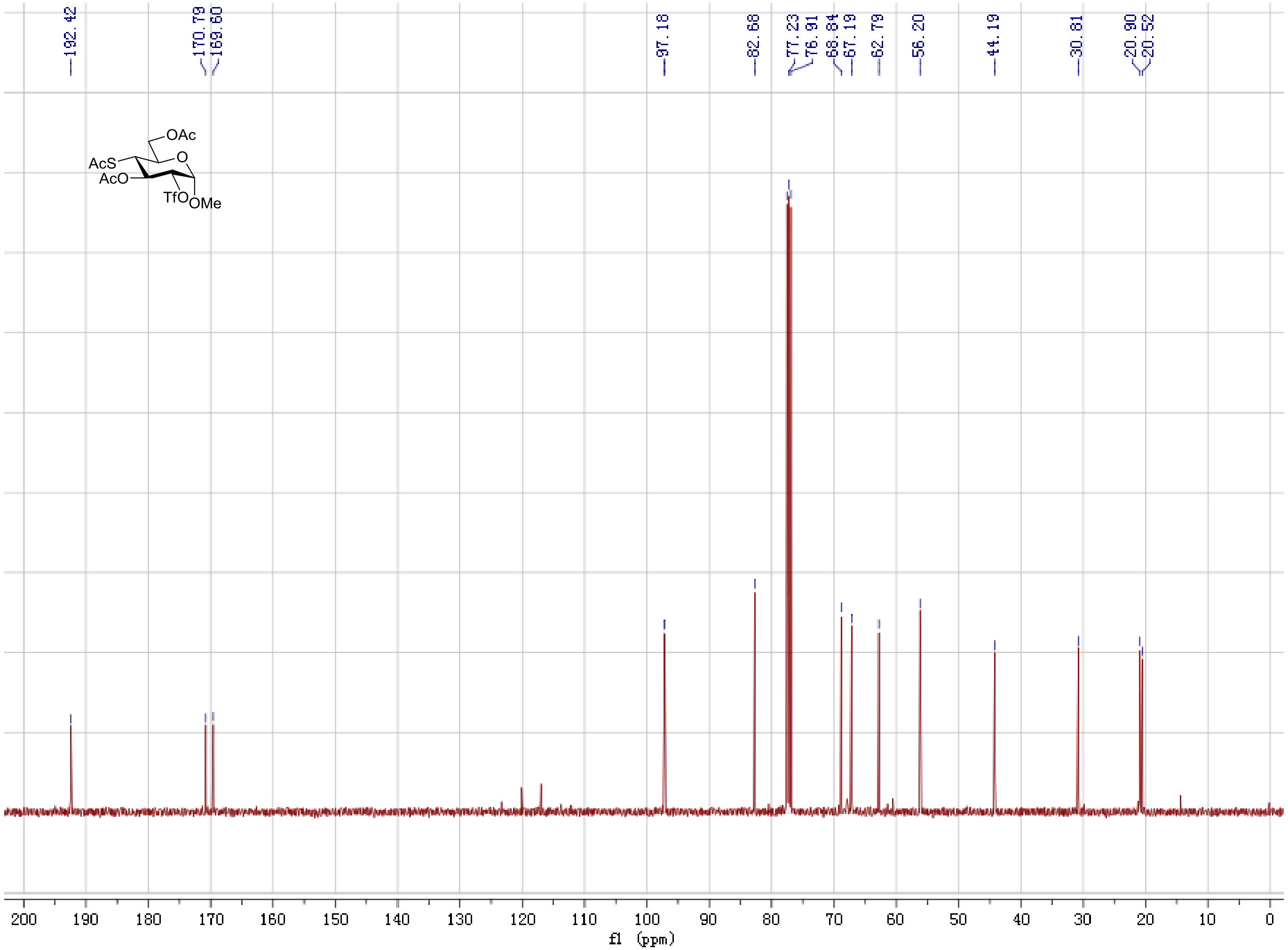

Figure S18. ^{13}C NMR spectrum (100 MHz) of **7** in $CDCl_3$

Methyl 4-*S*-acetyl-3,6-di-*O*-acetyl-2-*O*-triflyl-*β*-D-glucopyranoside 8

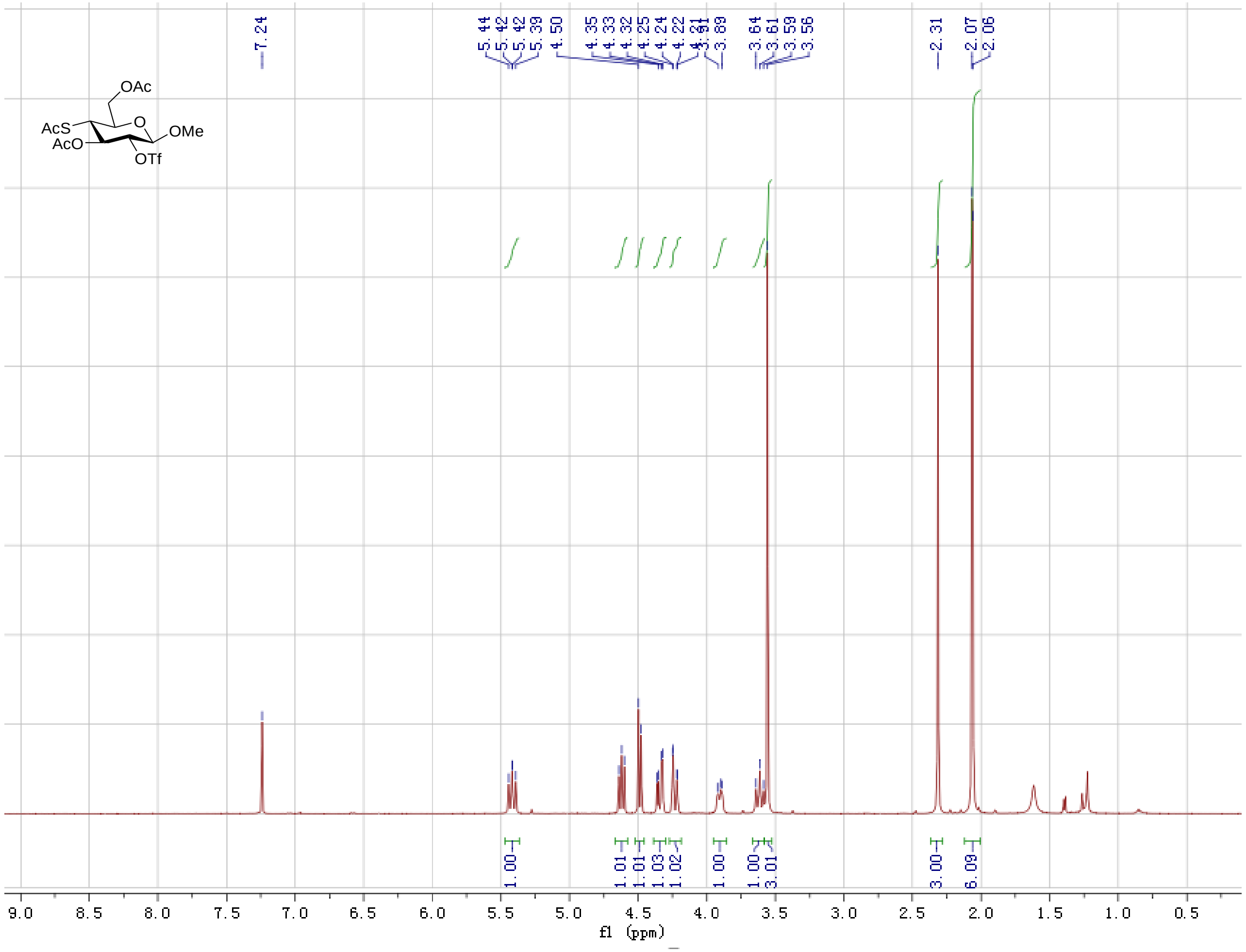

Figure S19. ^{1}H NMR spectrum (400 MHz) of **8** in $CDCl_3$

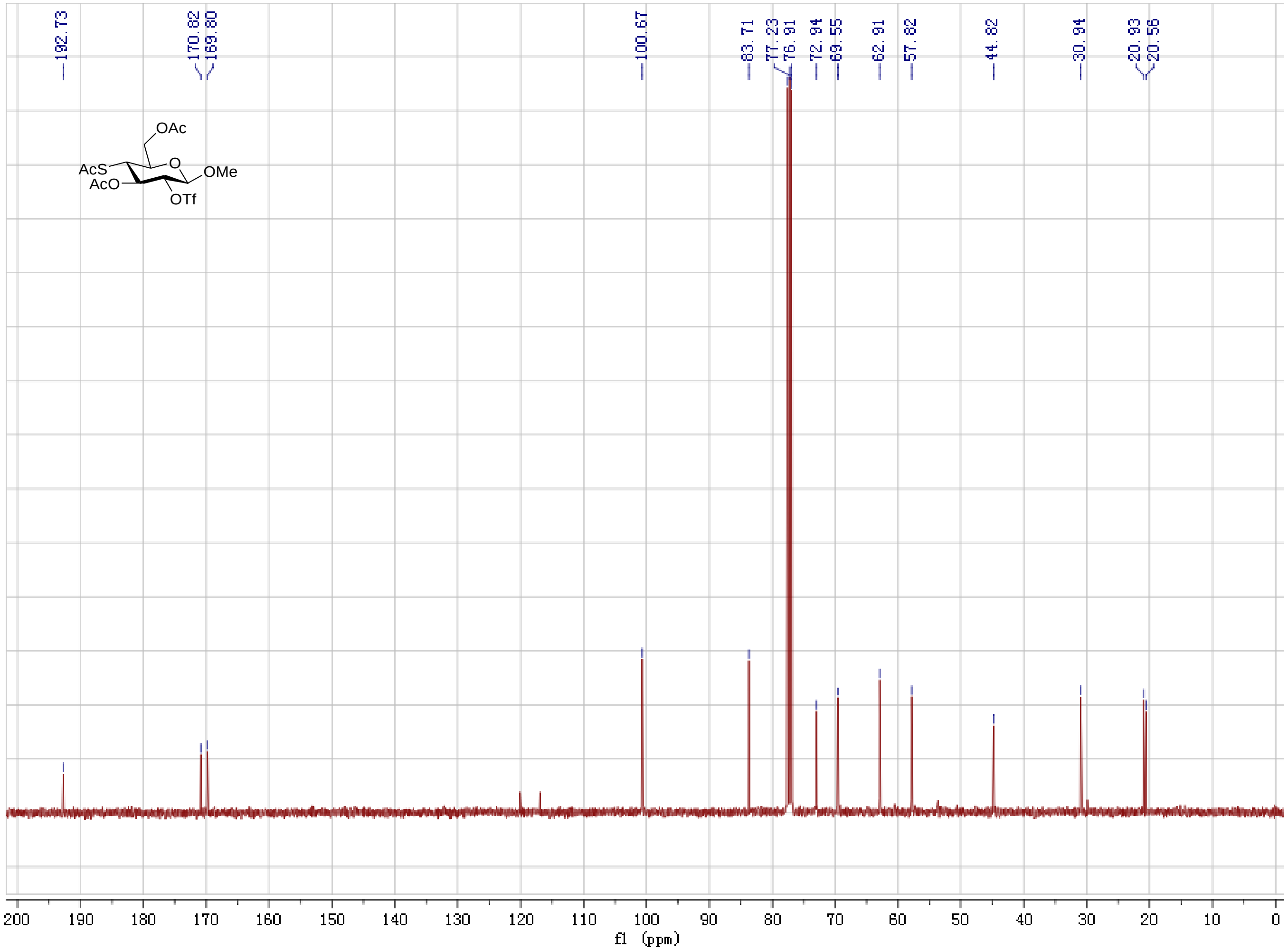

Figure S20. ^{13}C NMR spectrum (100 MHz) of **8** in $CDCl_3$

Methyl 2,4-di-*S*-acetyl-3,6-di-*O*-acetyl-*a*-D-mannopyranoside 9

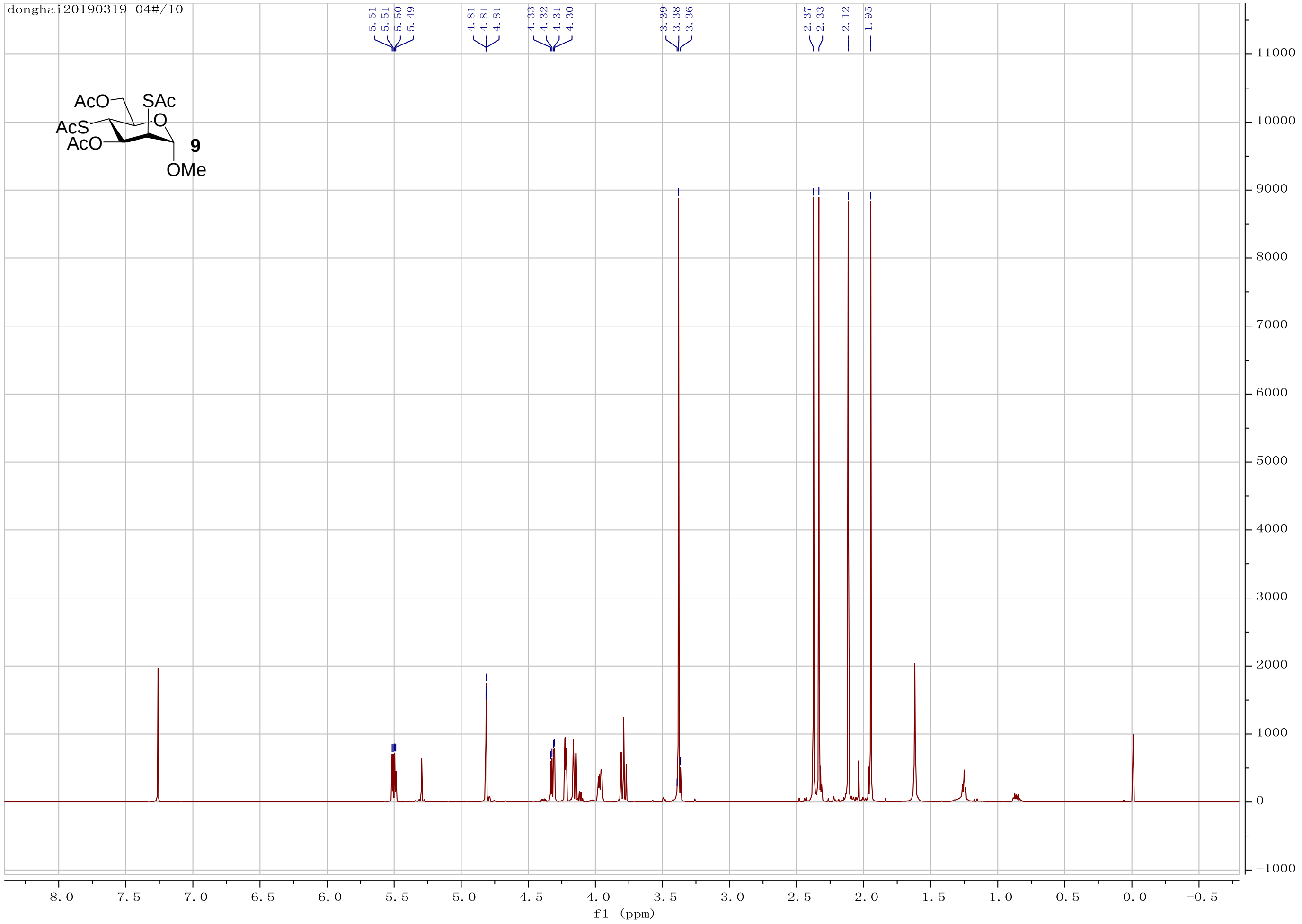

Figure S21. ^{1}H NMR spectrum (400 MHz) of **9** in $CDCl_3$

Methyl 2,4-di-*S*-acetyl-3,6-di-*O*-acetyl-*β*-D-mannopyranoside 10

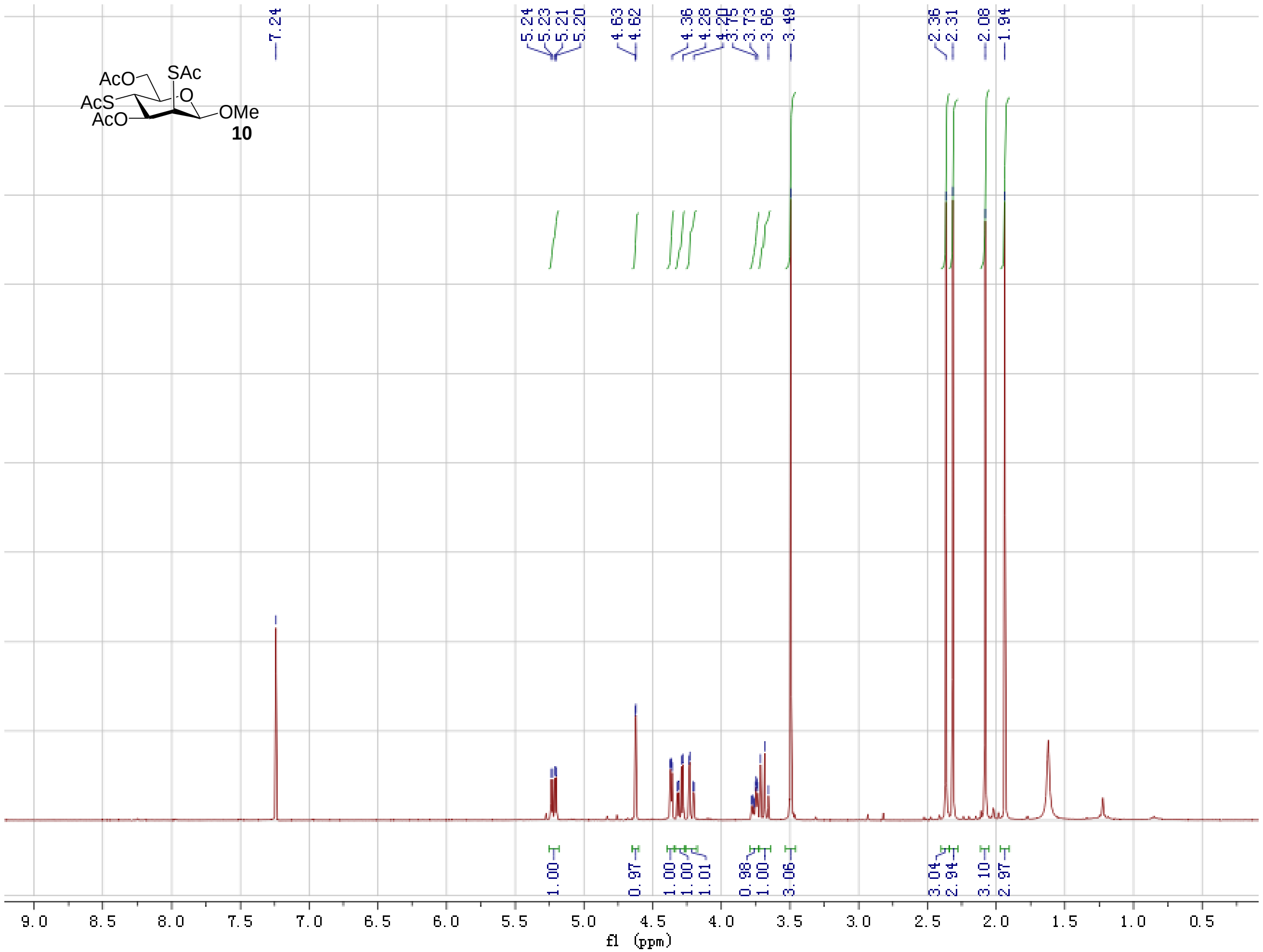

Figure S22. ^{1}H NMR spectrum (400 MHz) of **10** in $CDCl_3$

Methyl 2,4-di-*S*-acetyl-3,6-di-*O*-acetyl-*α*-D-talopyranoside 19

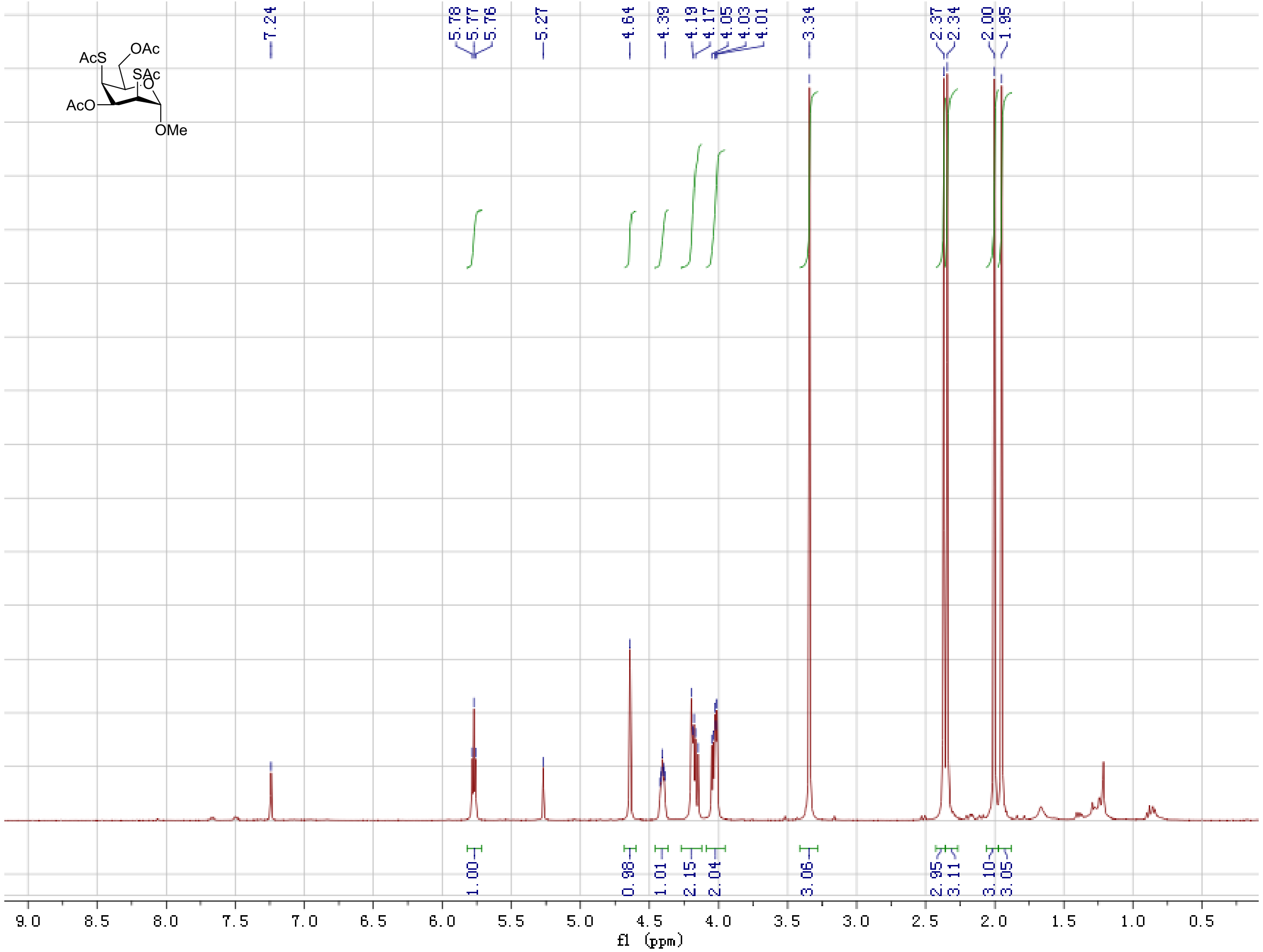

Figure S23. ^{1}H NMR spectrum (400 MHz) of **19** in $CDCl_3$

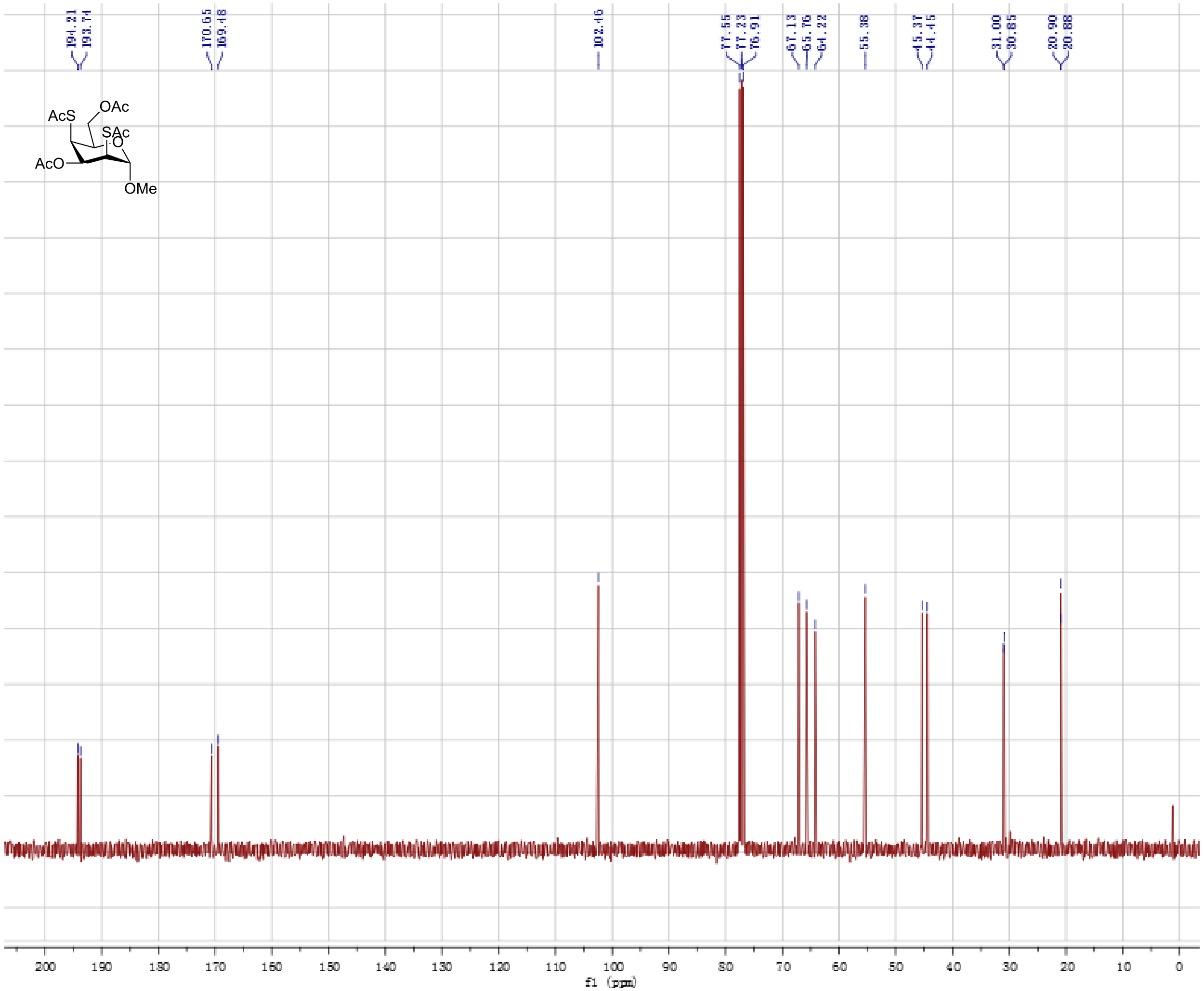

Figure S24. ^{13}C NMR spectrum (100 MHz) of **19** in $CDCl_3$

Compound 19a

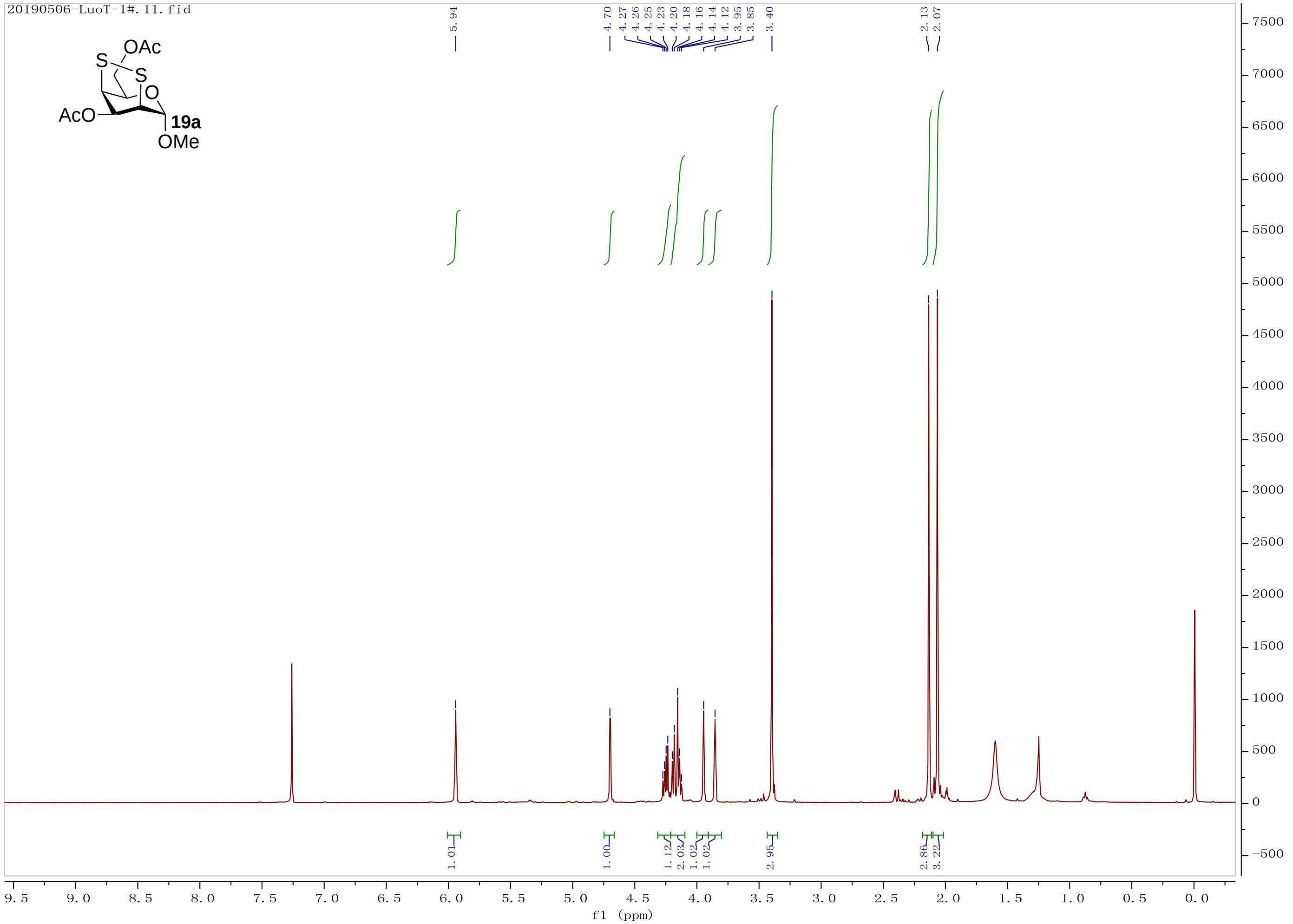

Figure S25. ^{1}H NMR spectrum (400 MHz) of **19a** in $CDCl_3$

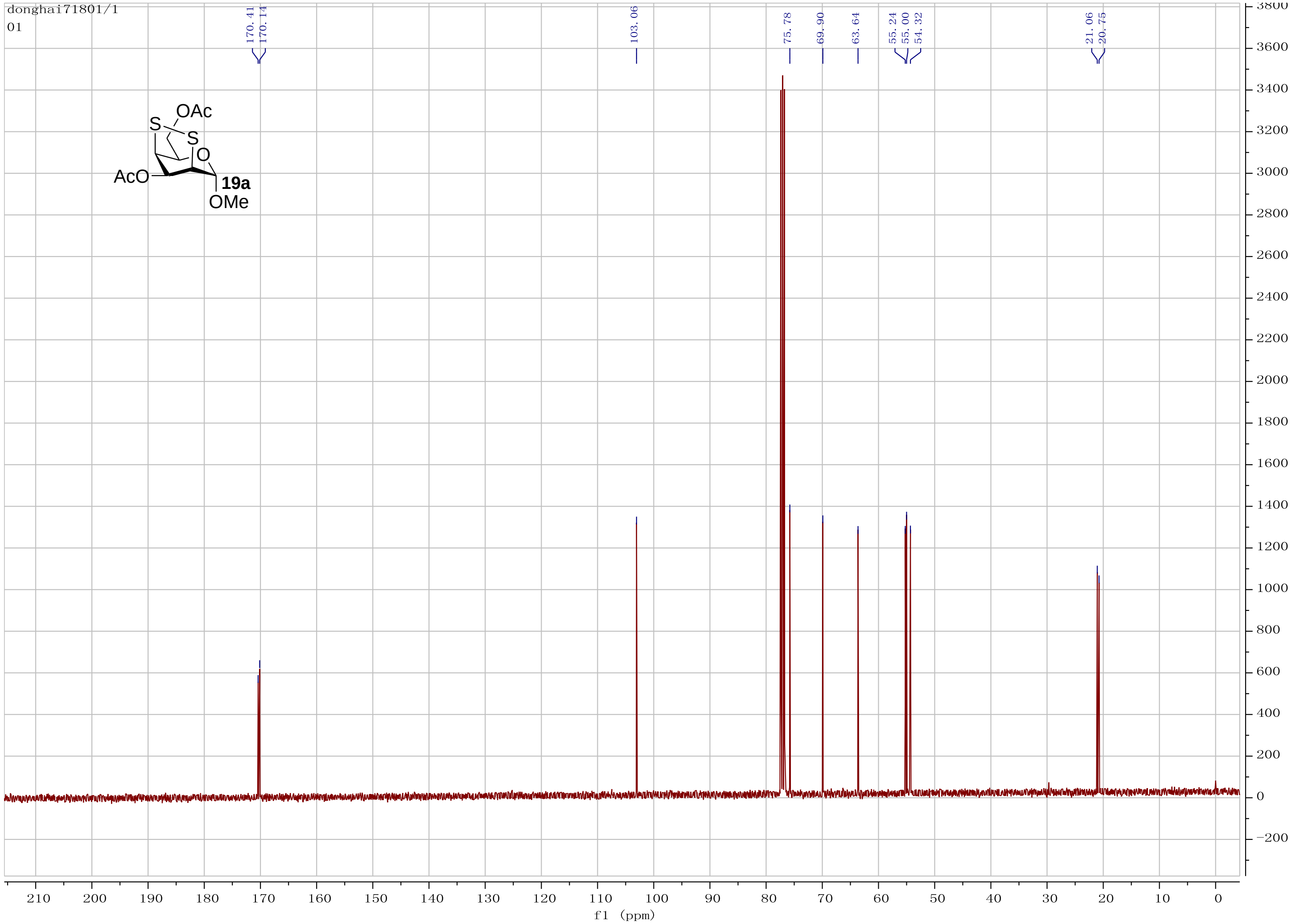

Figure S26. ^{13}C NMR spectrum (100 MHz) of **19a** in $CDCl_3$

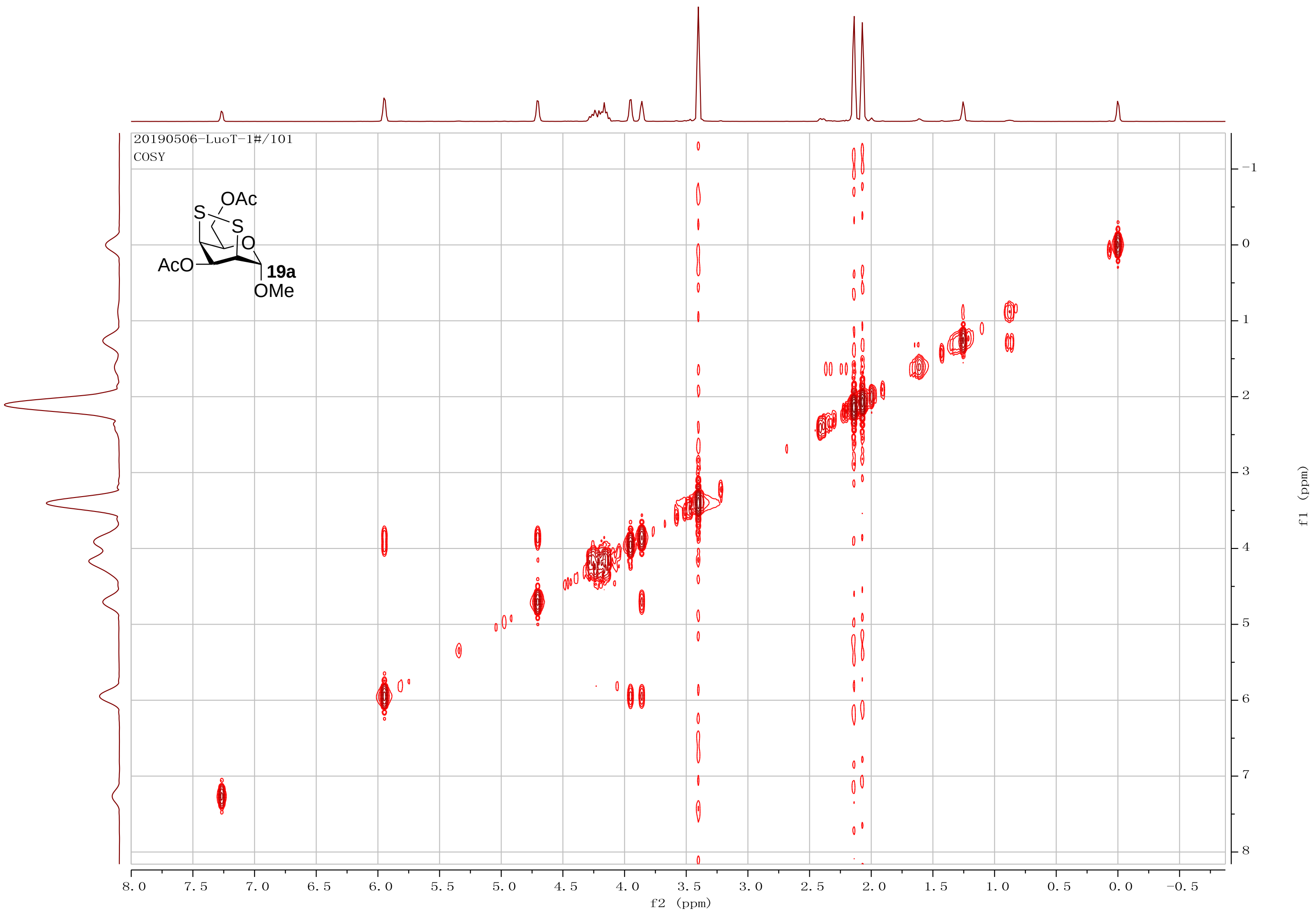

Figure S27. ^{1}H-^{1}H COSY spectrum of **19a** in $CDCl_3$

Methyl 2,4-di-*S*-acetyl-3,6-di-*O*-acetyl-*β*-D-talopyranoside 20

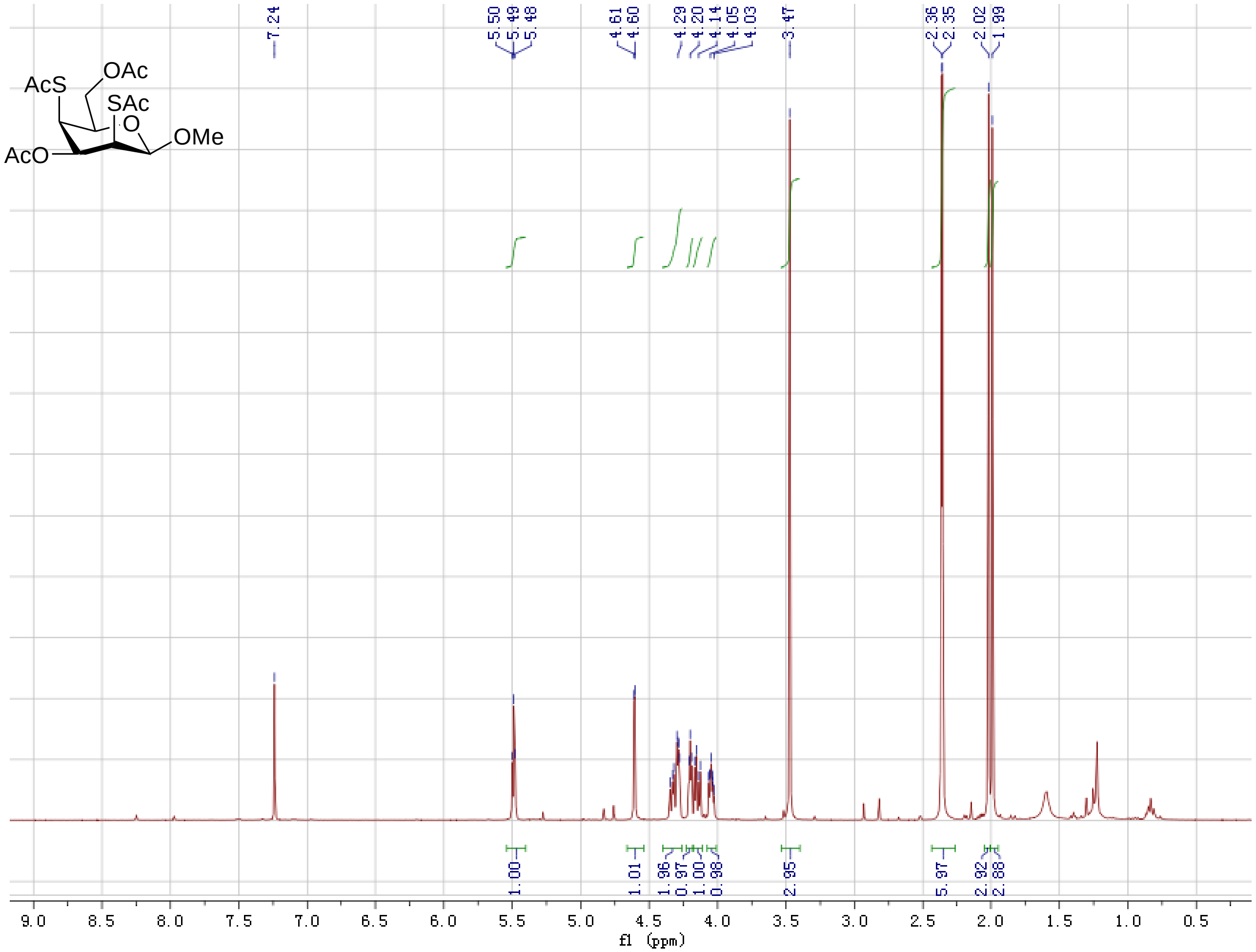

Figure S28. ^{1}H NMR spectrum (400 MHz) of **20** in

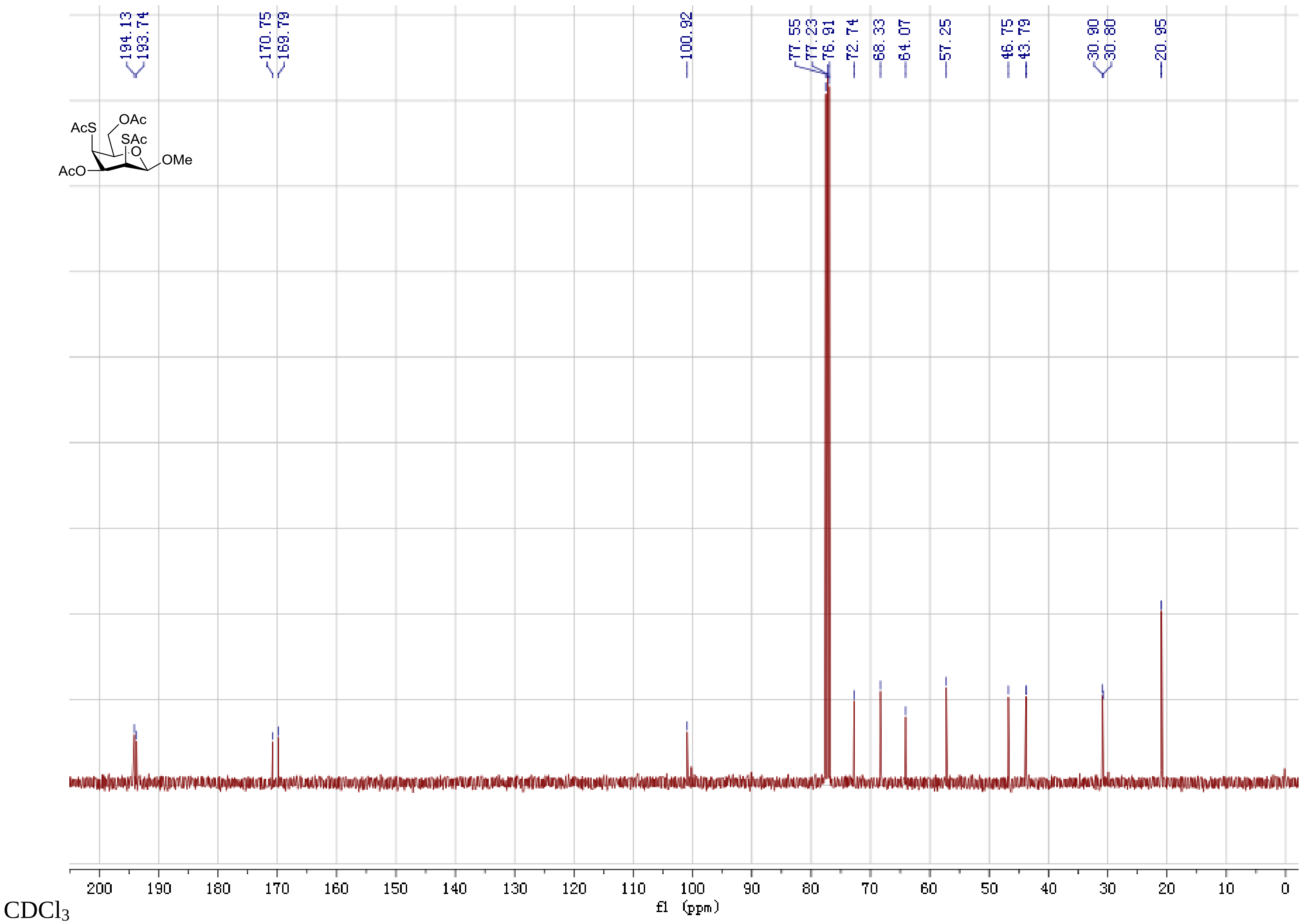

CDCl$_3$

Figure S29. ^{13}C NMR spectrum (100 MHz) of **20** in CDCl$_3$

Methyl 4-*S*-acetyl-2, 3, 6-tri-*O*-acetyl-*a*-D-talapyranoside 21

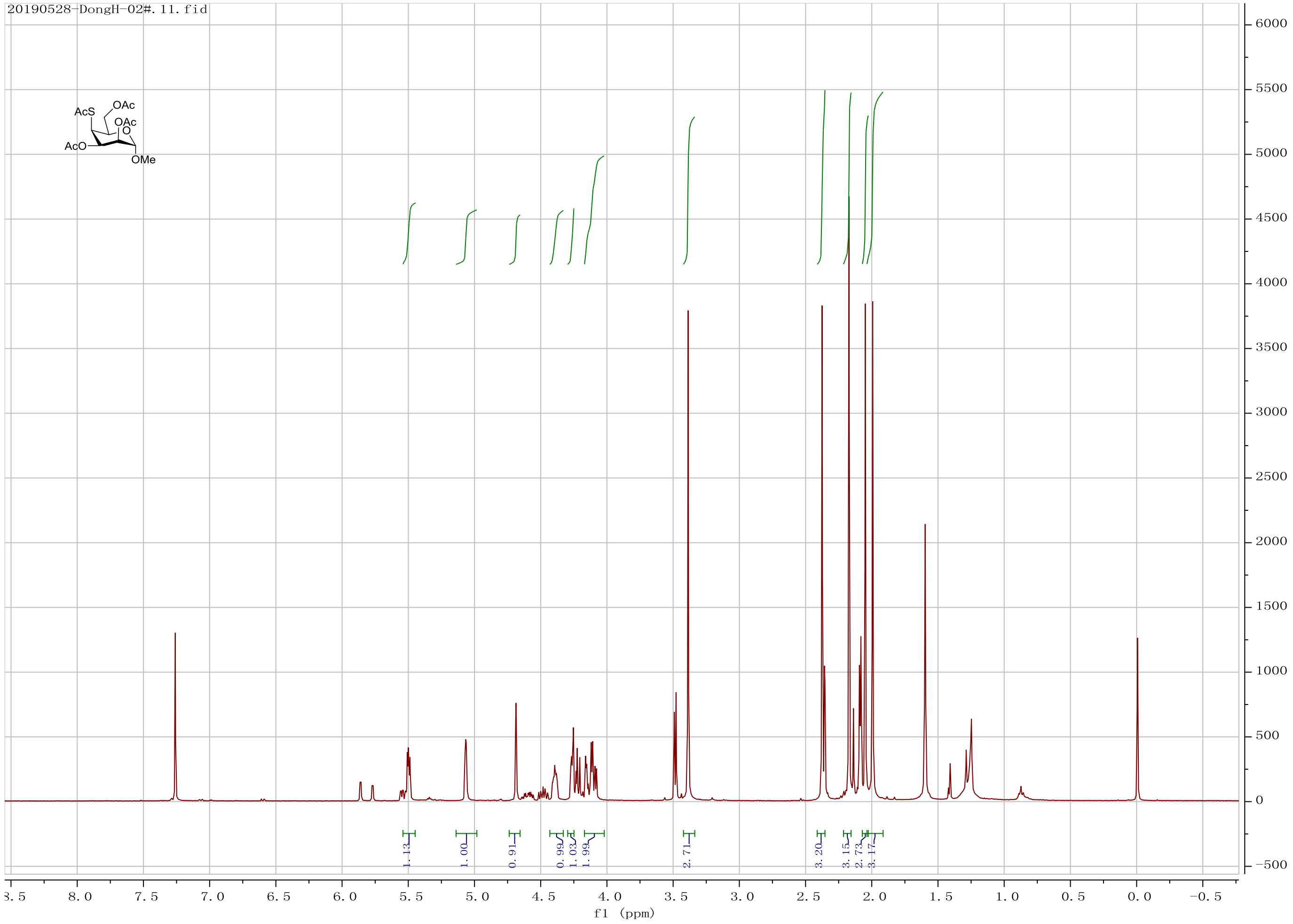

Figure S30. ^{1}H NMR spectrum (400 MHz) of **21** in $CDCl_3$

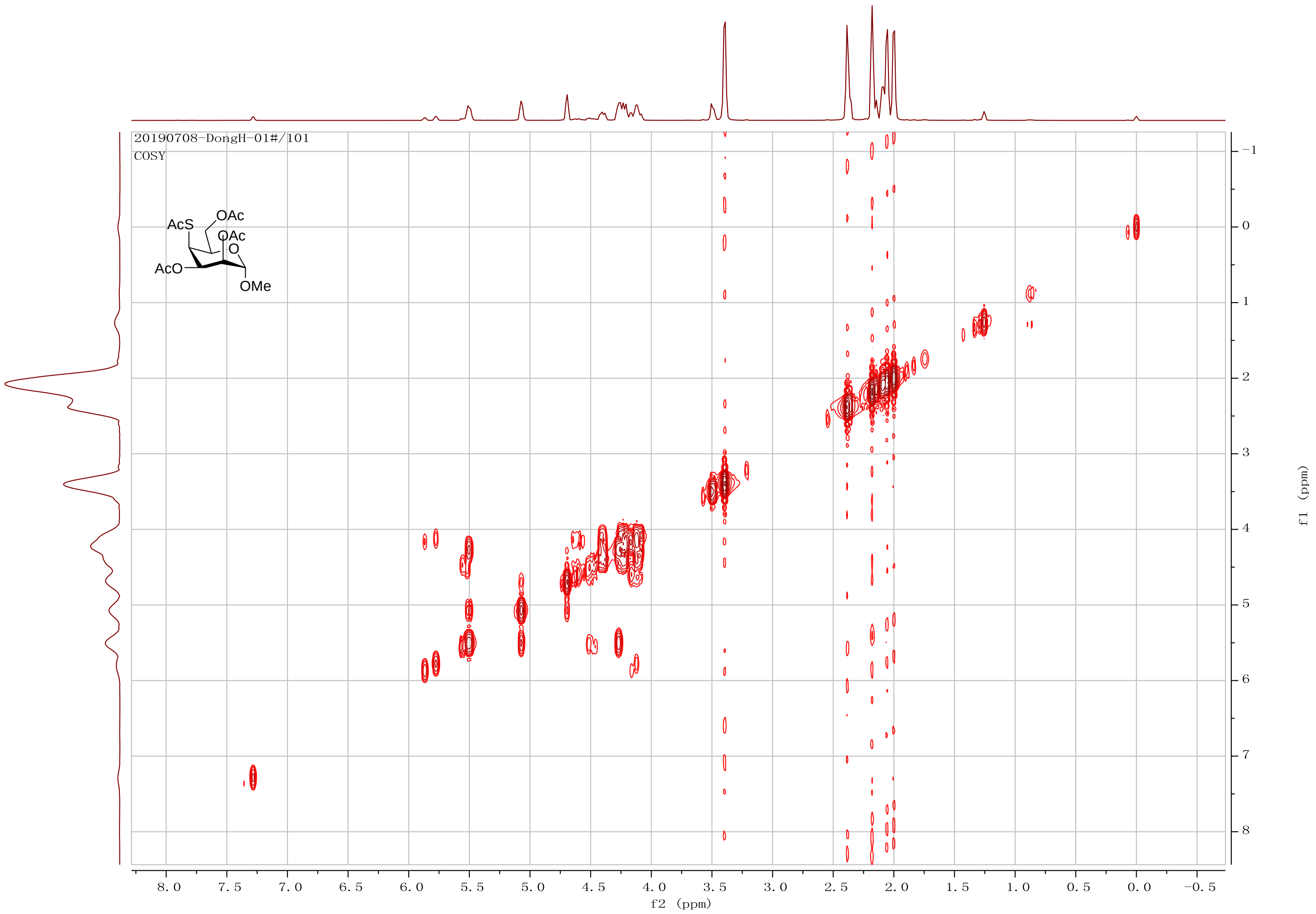

Figure S31. ^{1}H-^{1}H COSY spectrum of **21** in $CDCl_3$

Methyl 4-*S*-acetyl-2, 3, 6-tri-*O*-acetyl-*β*-D-talapyranoside 22

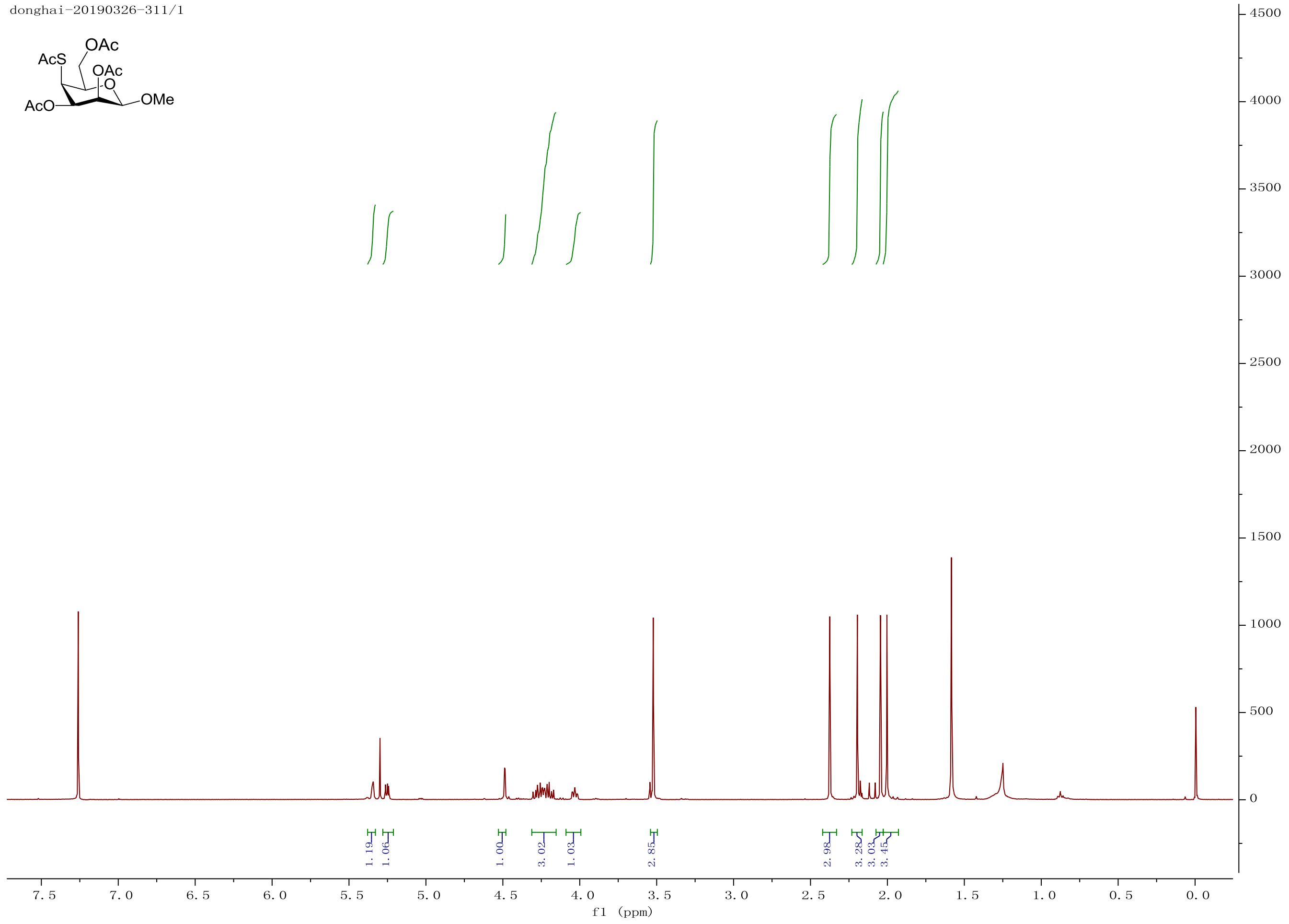

Figure S32. ^{1}H NMR spectrum (400 MHz) of **22** in $CDCl_3$

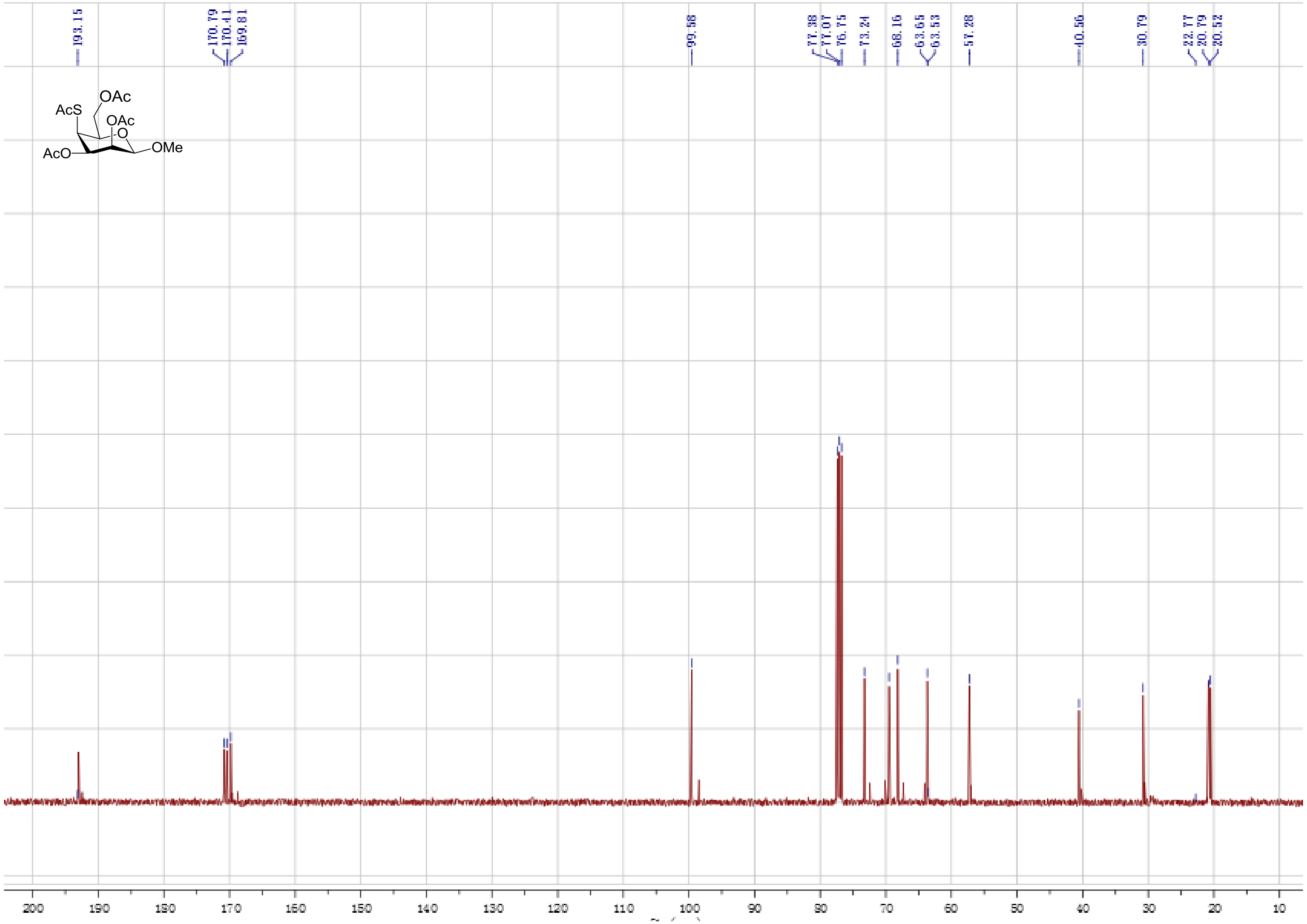

Figure S33. ^{13}C NMR spectrum (100 MHz) of **22** in $CDCl_3$

Methyl 4-*S*-acetyl-3,6-di-*O*-acetyl-2-*O*-triflyl-*α*-D-galactopyranoside 23

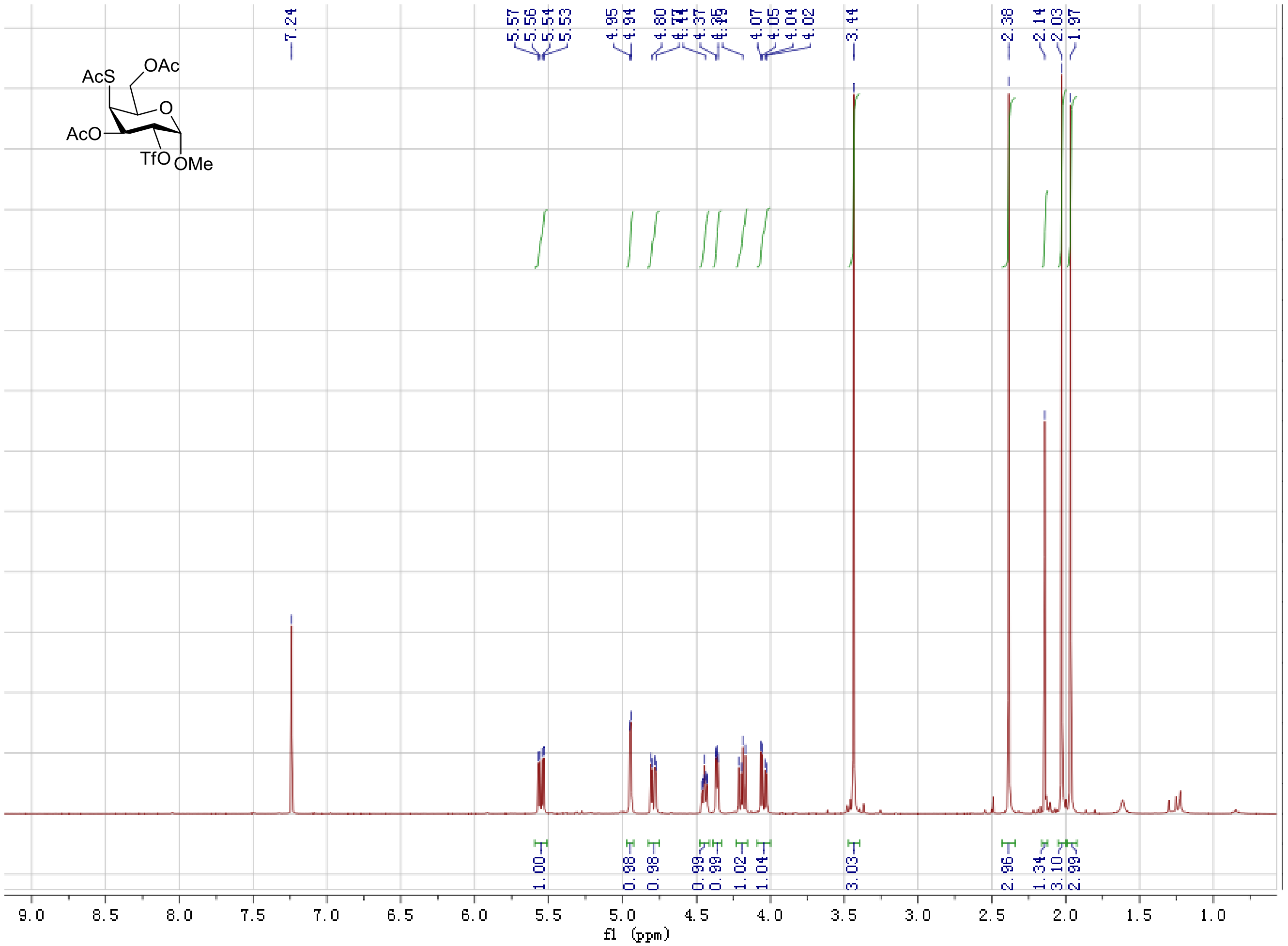

Figure S34. ^{1}H NMR spectrum (400 MHz) of **23** in $CDCl_3$

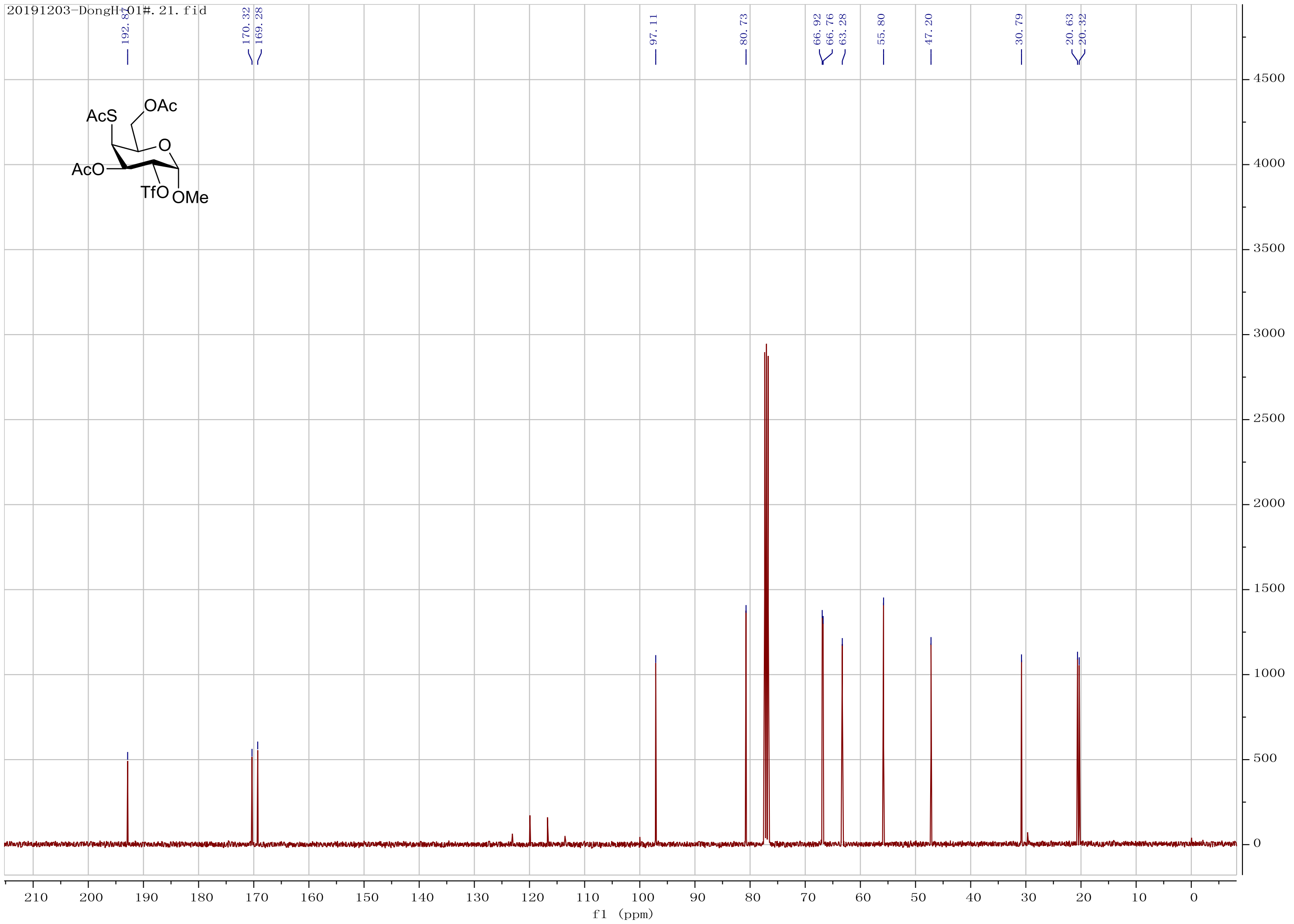

Figure S35. ^{1}H NMR spectrum (400 MHz) of **23** in $CDCl_3$

Methyl 4-*S*-acetyl-3,6-di-*O*-acetyl-2-*O*-triflyl-*β*-D-galactopyranoside 24

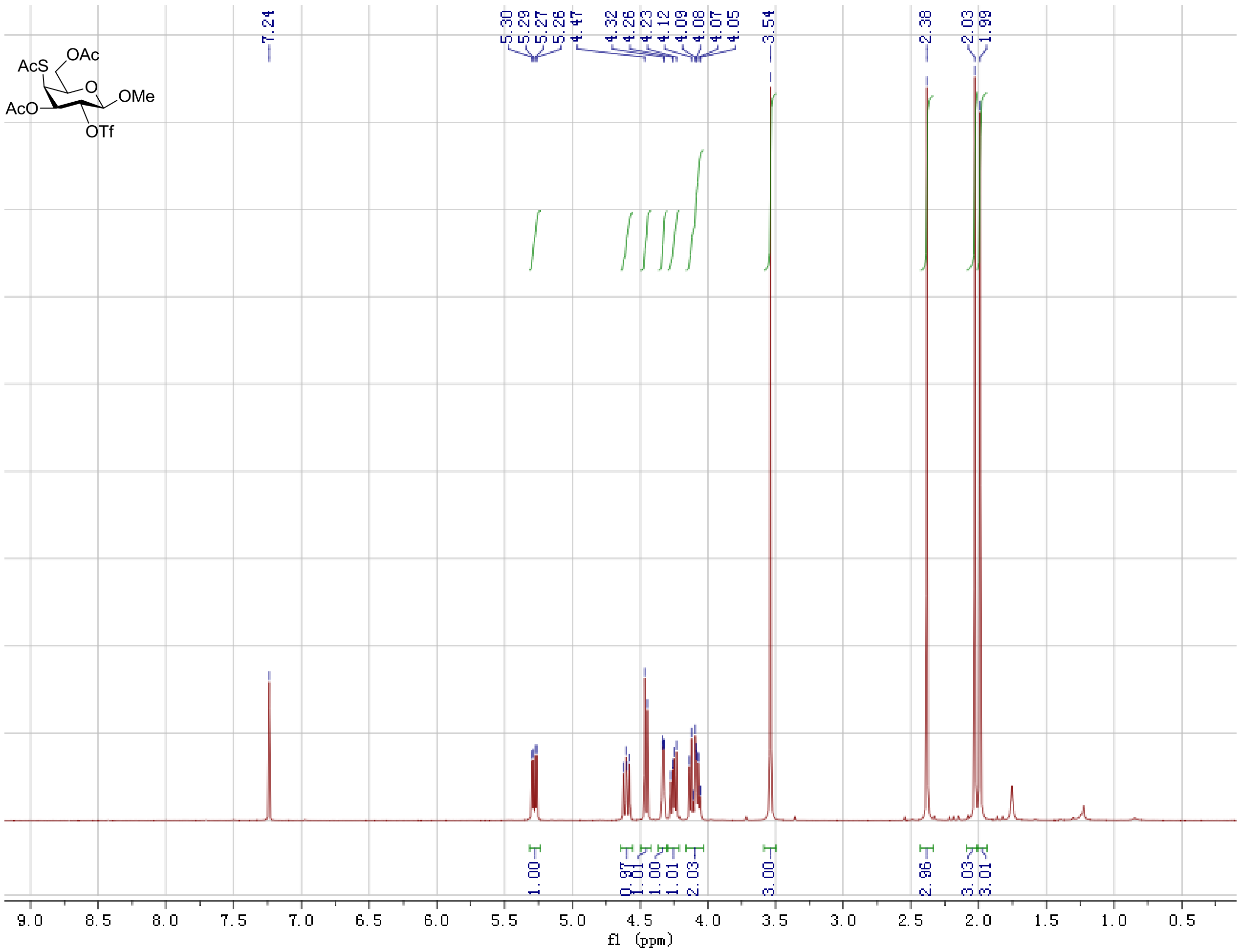

Figure S36. ^{1}H NMR spectrum (400 MHz) of **24** in $CDCl_3$

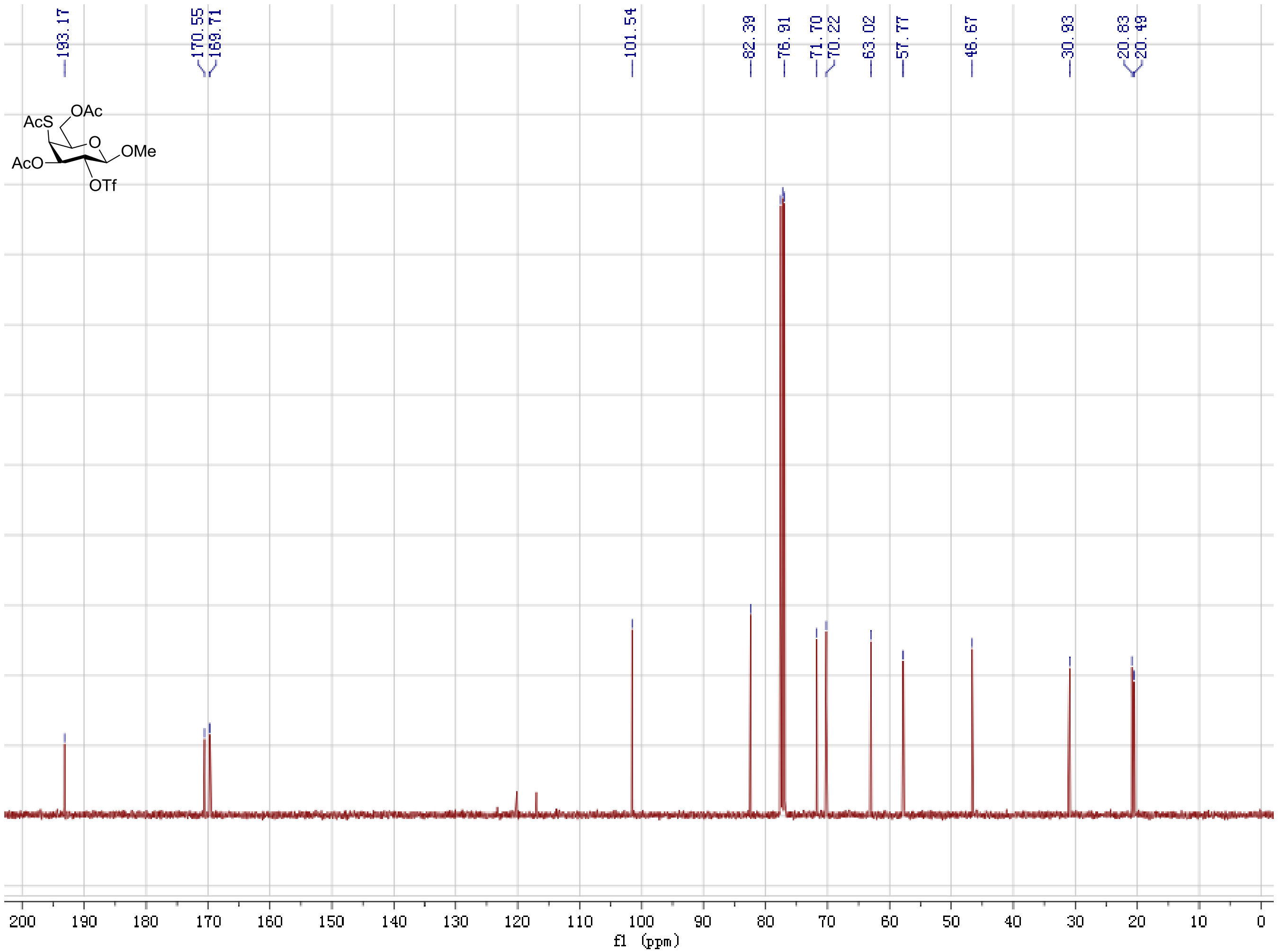

Figure S37. ^{13}C NMR spectrum (100 MHz) of **24** in $CDCl_3$

Methyl 4-*S*-acetyl-3, 6-di-*O*-acetyl-2-deoxy-*α*-D-erythro-hex-2-enopyranoside 29

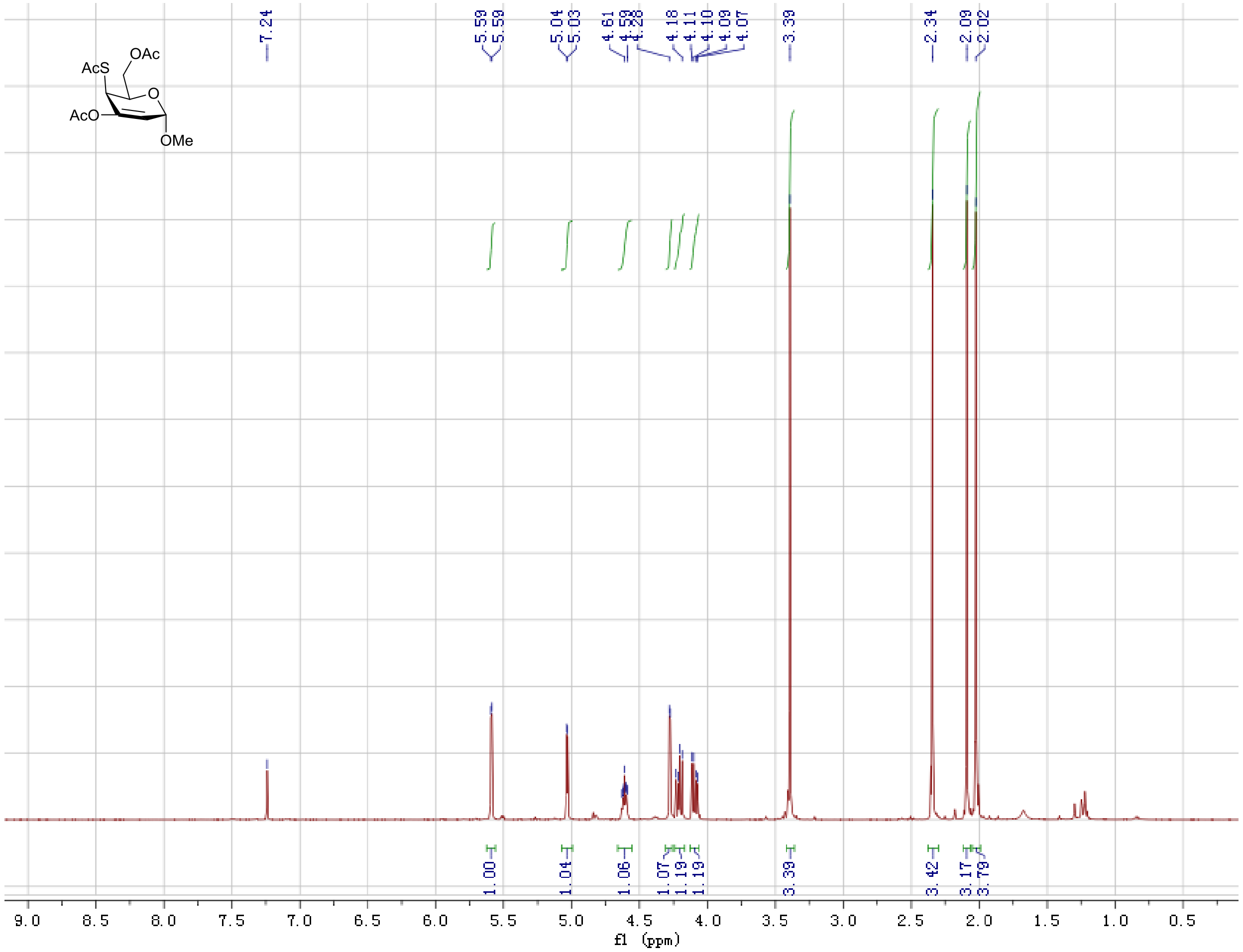

Figure S38. ^{1}H NMR spectrum (400 MHz) of **29** in $CDCl_3$

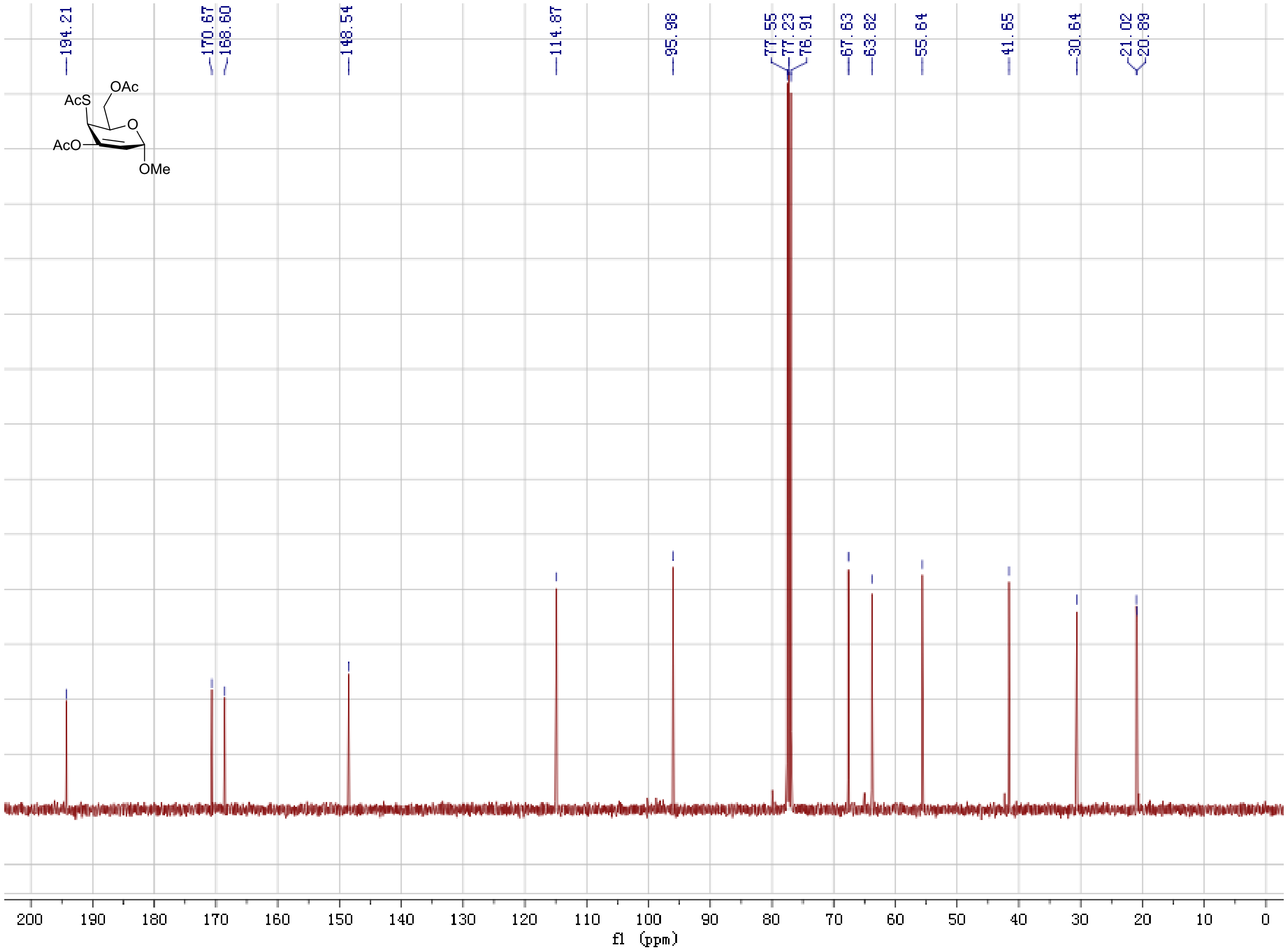

Figure S39. ^{13}C NMR spectrum (100 MHz) of **29** in $CDCl_3$

Methyl 4-*S*-acetyl- 6-*O*-acetyl-2-deoxy-3-keto-α-D-pyranoside 28

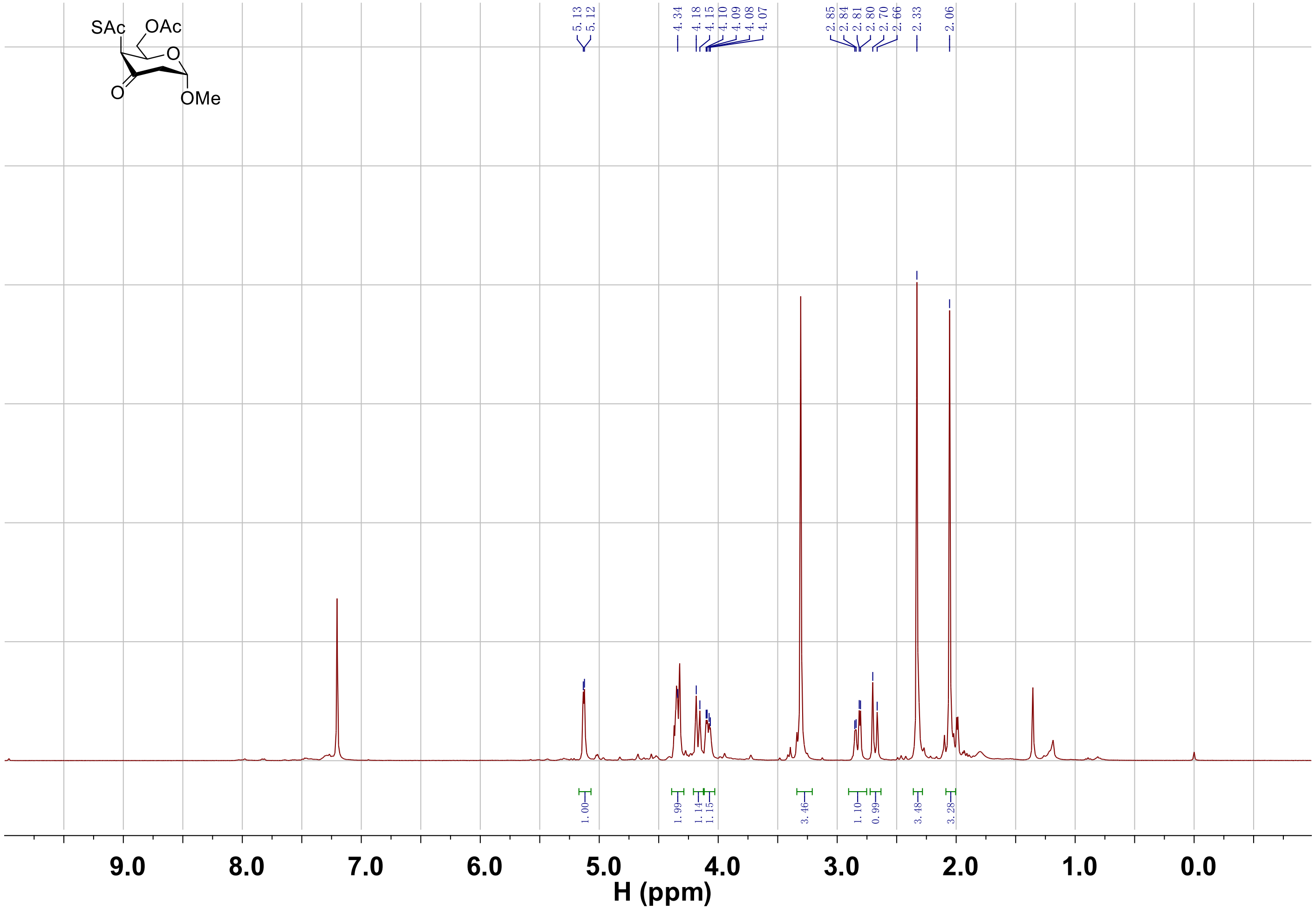

Figure S40. ^{1}H NMR spectrum (400 MHz) of **28** in $CDCl_3$

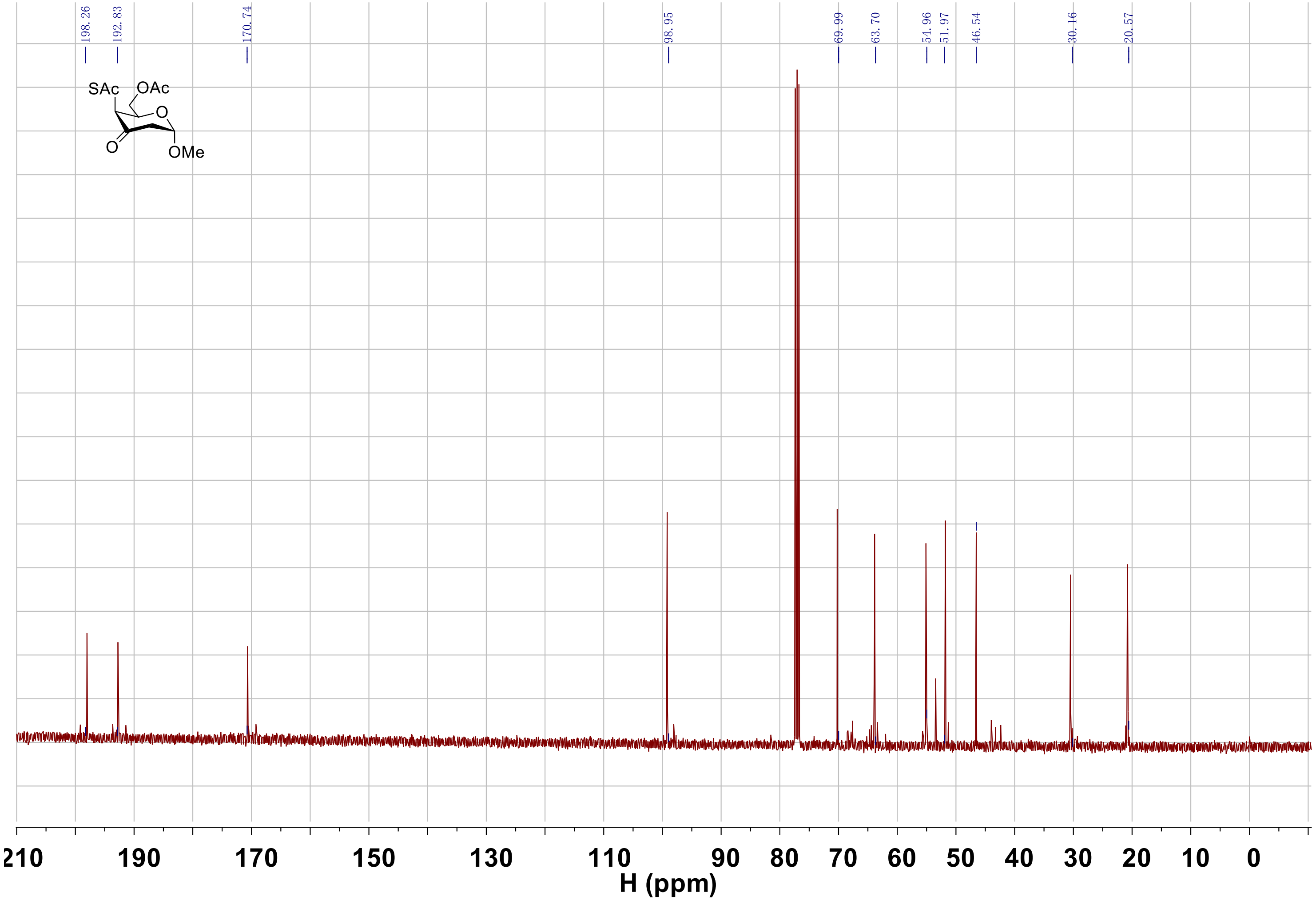

Figure S41. ^{13}C NMR spectrum (100 MHz) of **28** in $CDCl_3$

Methyl 4, 6-*O*-acetyl-2-deoxy-3-keto-α-D-pyranoside 30

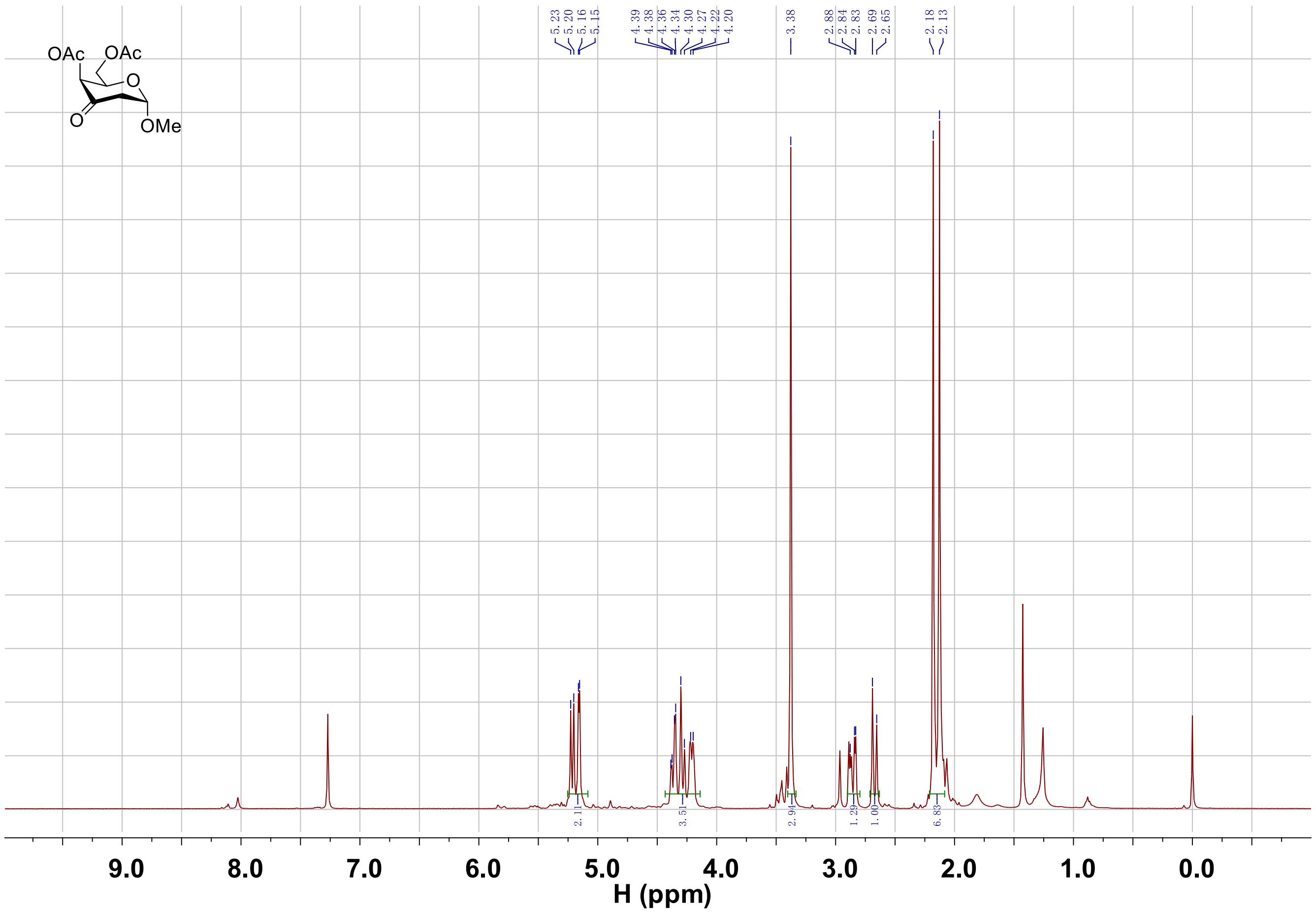

Figure S40. ^{1}H NMR spectrum (400 MHz) of **30** in $CDCl_3$

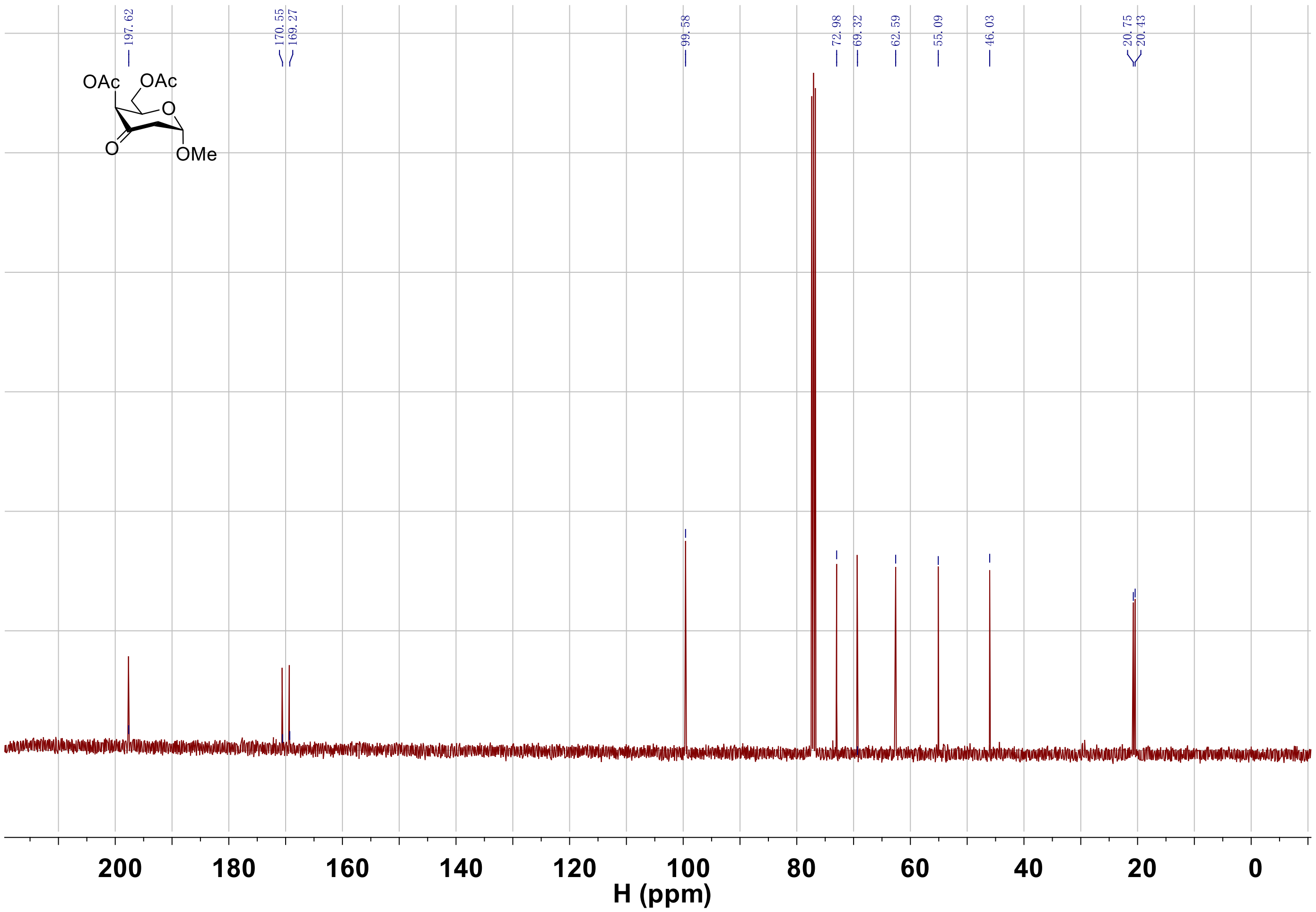

Figure S43. ^{13}C NMR spectrum (100 MHz) of **30** in $CDCl_3$

Methyl 2,4-di-*S*-acetyl-3,6-Di-*O*-acetyl-*β*-D-galactopyranoside 35

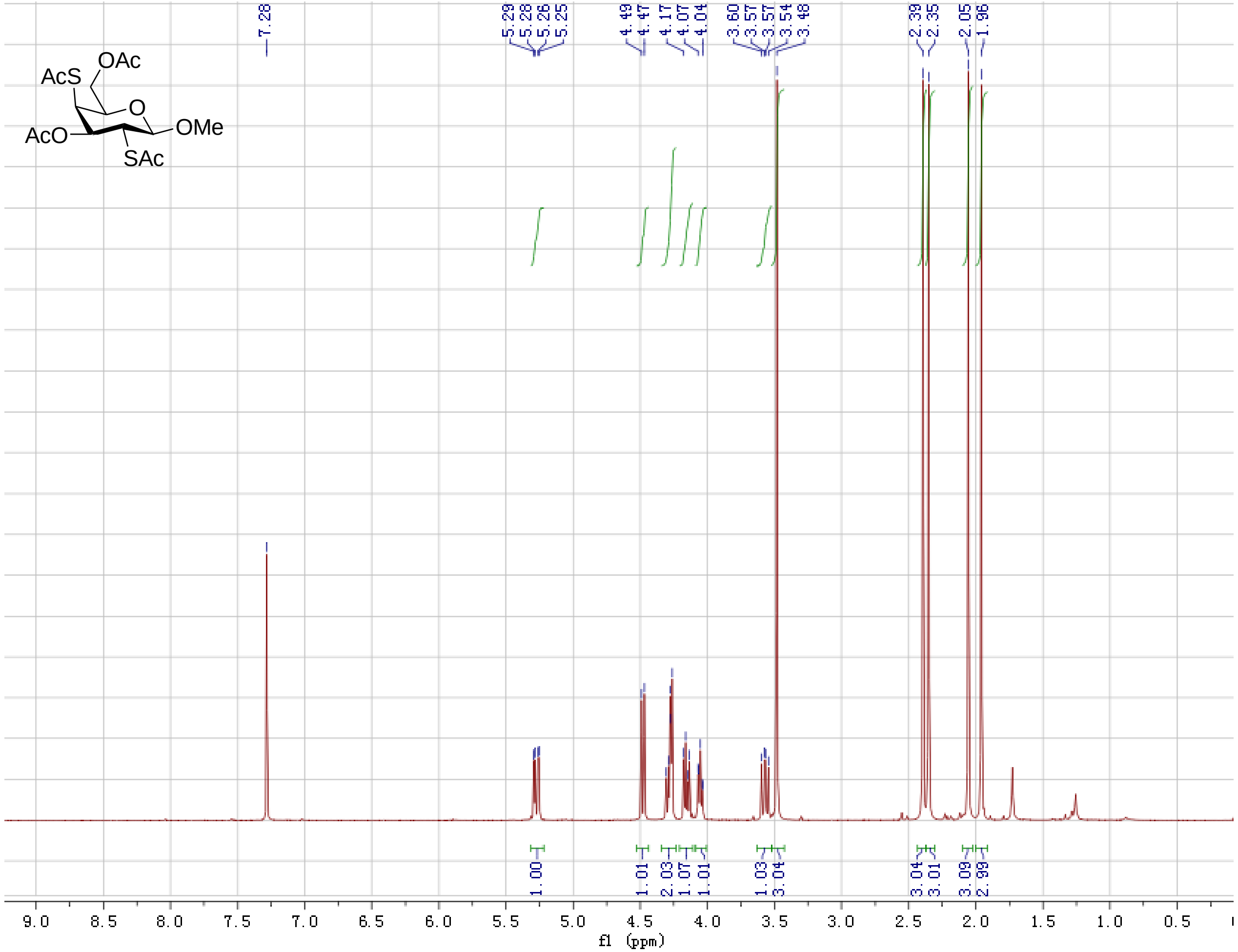

Figure S44. ^{1}H NMR spectrum (400 MHz) of **35** in $CDCl_3$

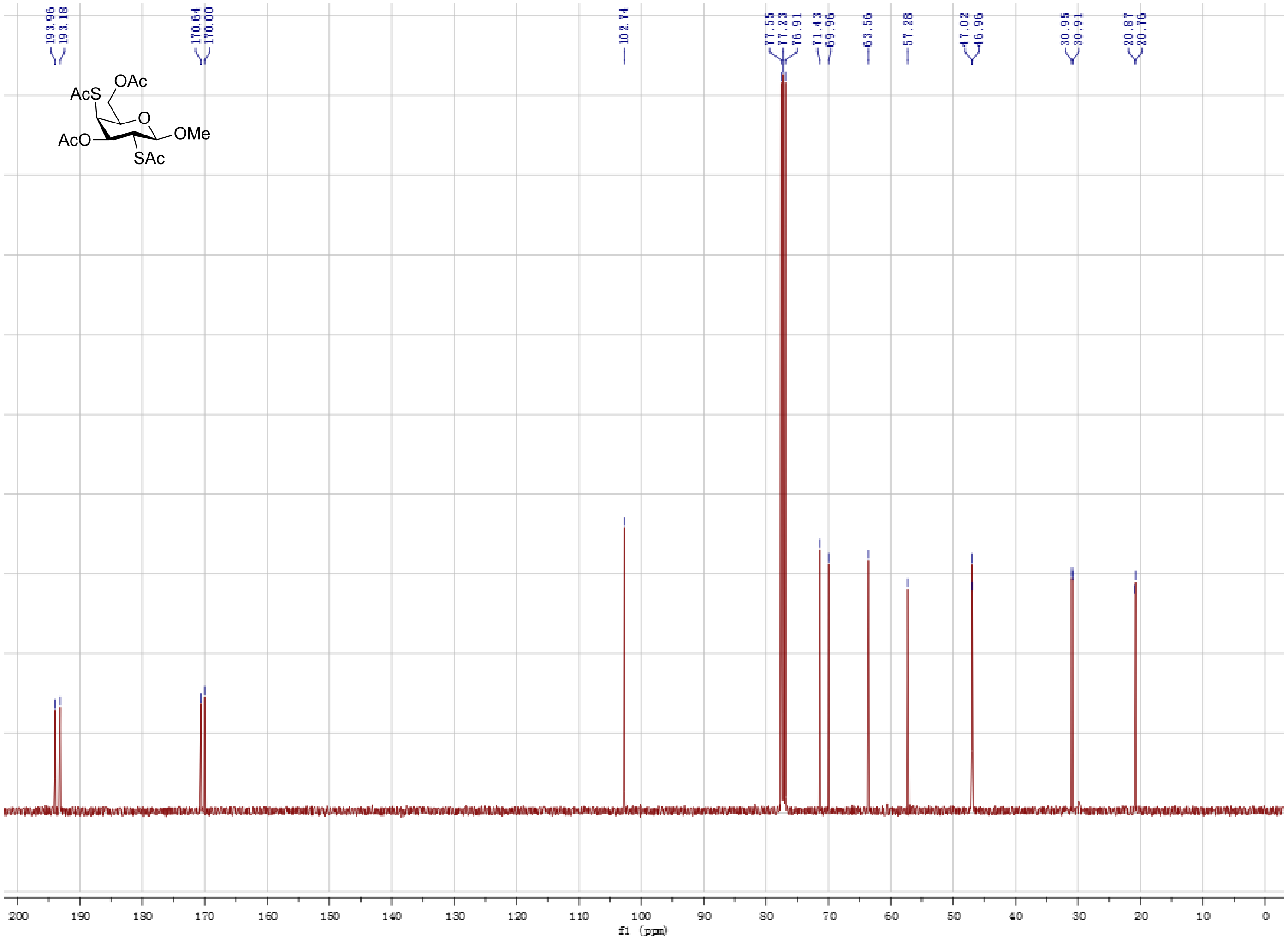

Figure S45. ^{13}C NMR spectrum (100 MHz) of **35** in $CDCl_3$

Methyl 4-*S*-acetyl-2,3,6-tri-*O*-acetyl-*β*-D-galactopyranoside 39

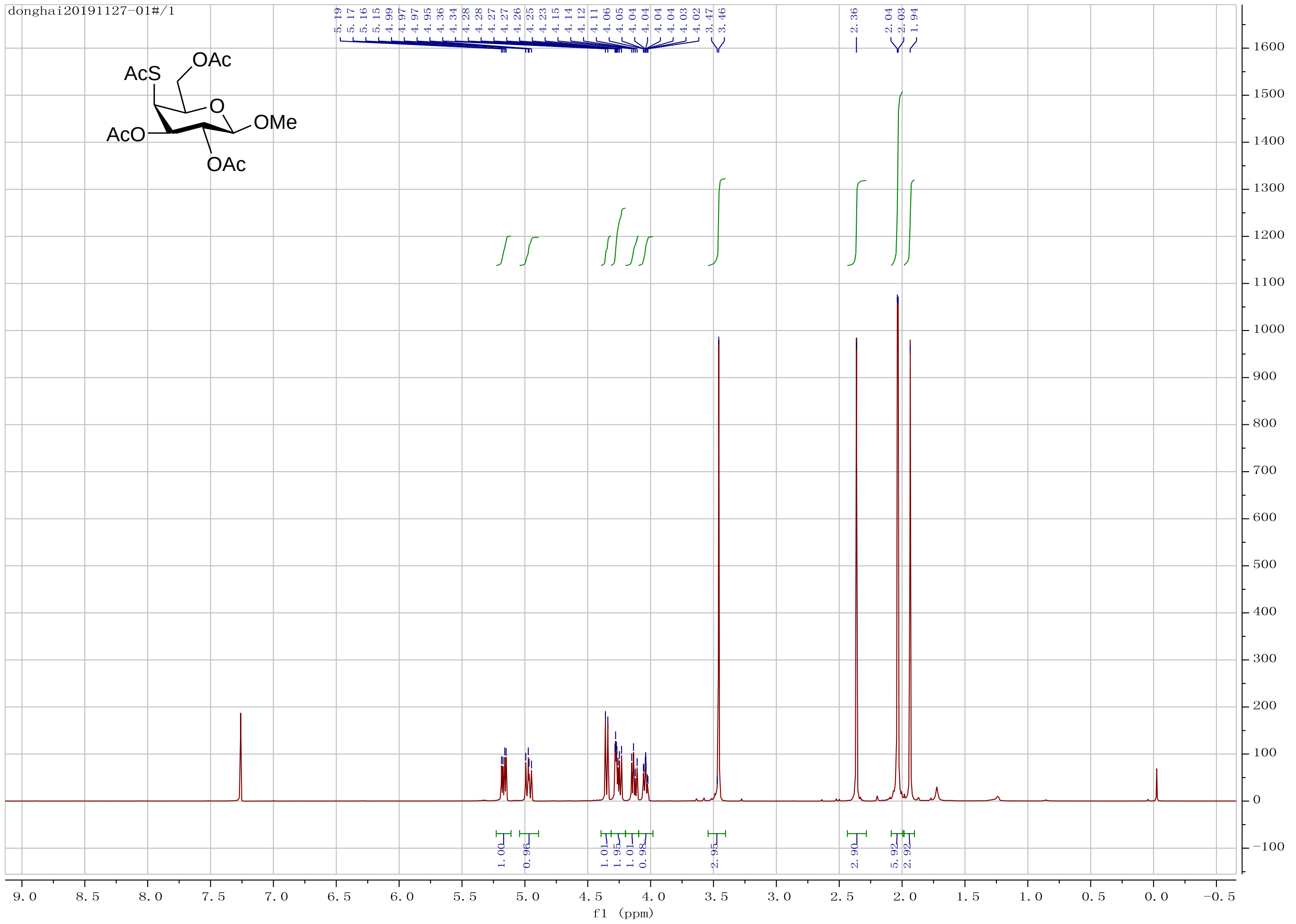

Figure S46. ^{1}H NMR spectrum (400 MHz) of **39** in $CDCl_3$

Methyl 6-*S*-acetyl-*α*-D-glucopyranoside 41

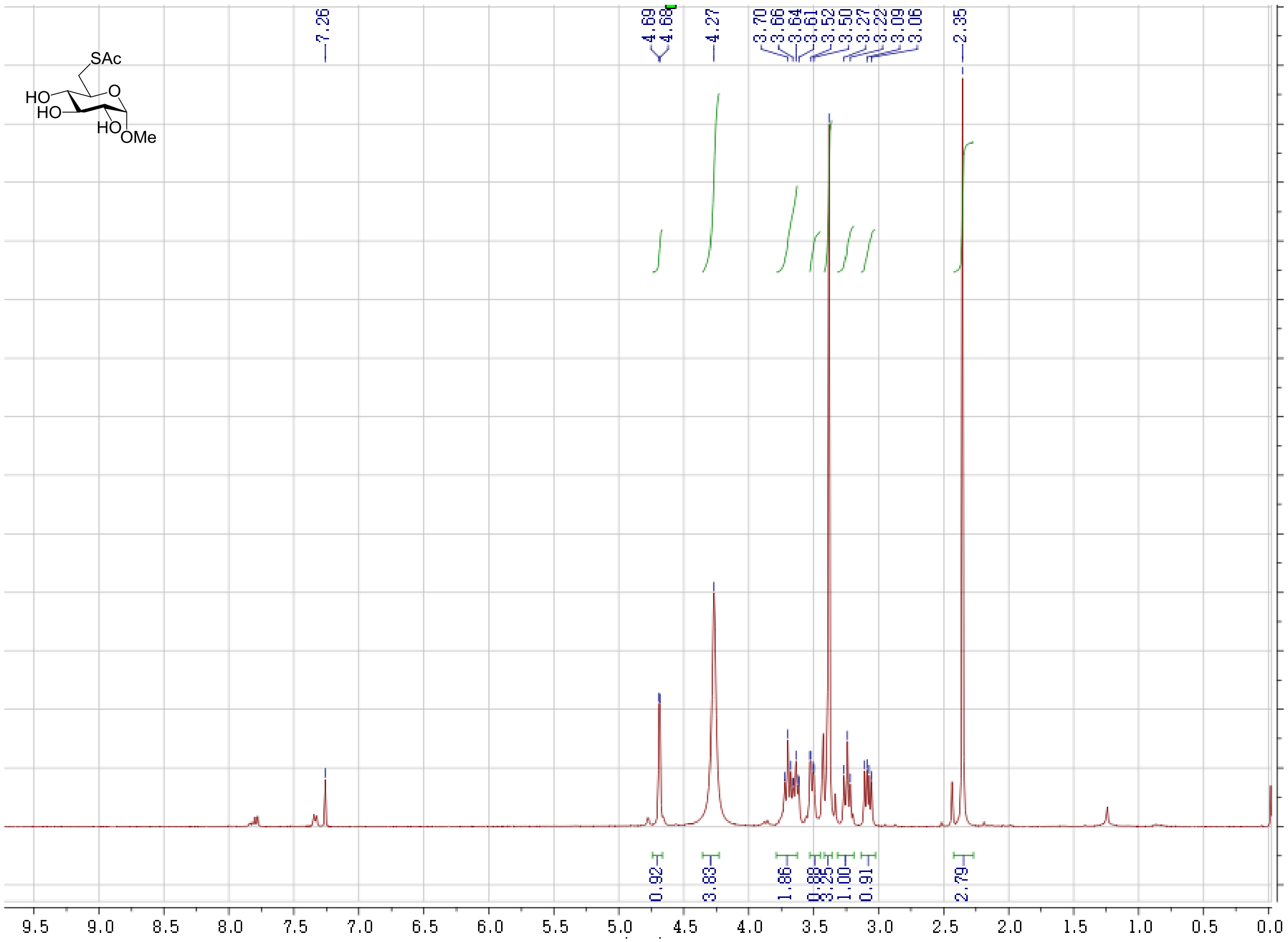

Figure S47. ^{1}H NMR spectrum (400 MHz) of **41** in $CDCl_3$

Methyl 6-*S*-acetyl-*β*--D-glucopyranosid 43

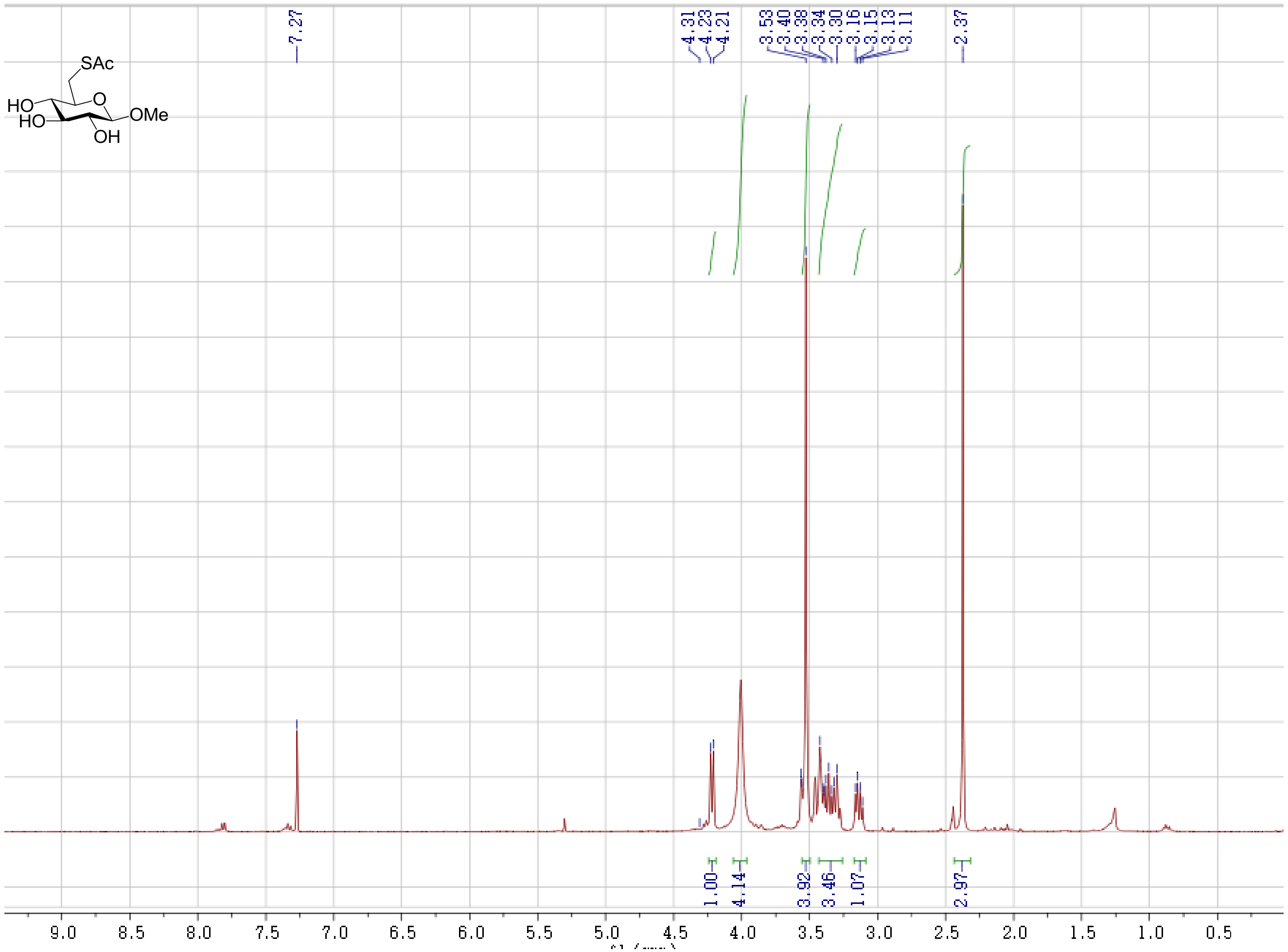

Figure S39. ^{1}H NMR spectrum (400 MHz) of **43** in $CDCl_3$

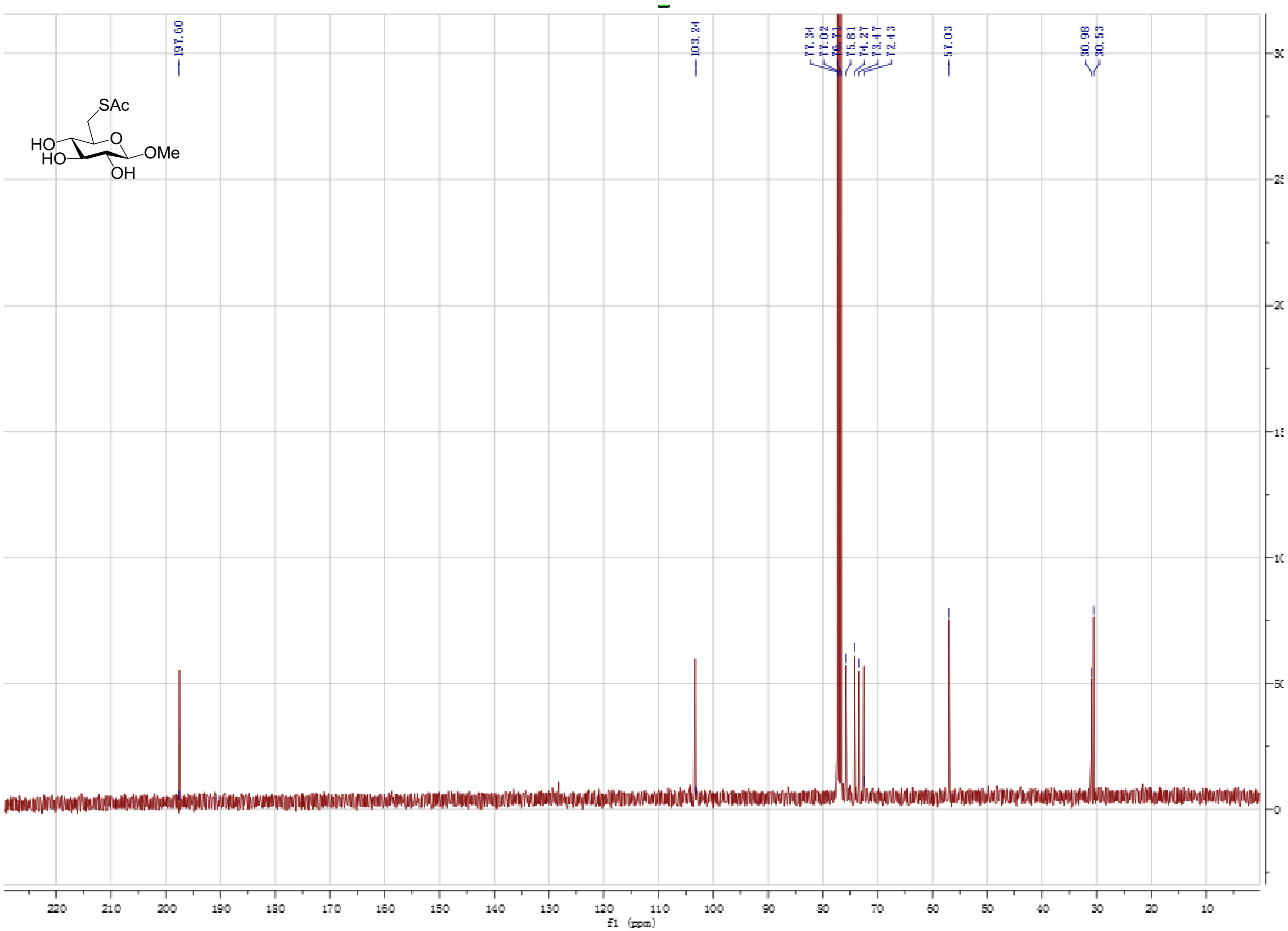

Figure S49. ^{13}C NMR spectrum (100 MHz) of **43** in $CDCl_3$

Methyl 6-S-acetyl-*a*-D-mannopyranside 45

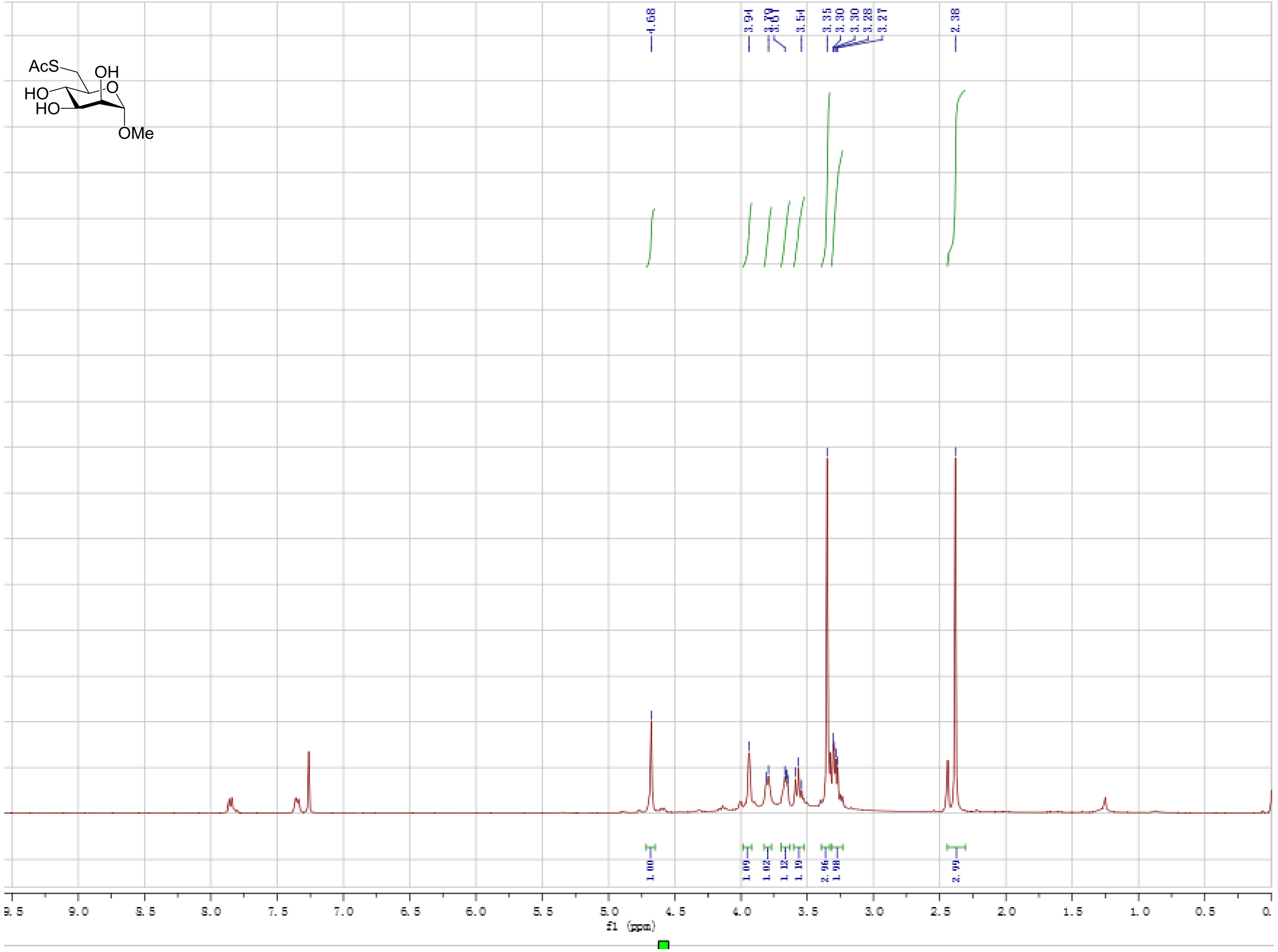

Figure S50. ^{1}H NMR spectrum (400 MHz) of **45** in $CDCl_3$

Methyl 2,3,6-tri-*O*-acetyl-4-deoxy-*a*-D-lyxo-hexopyranoside 46

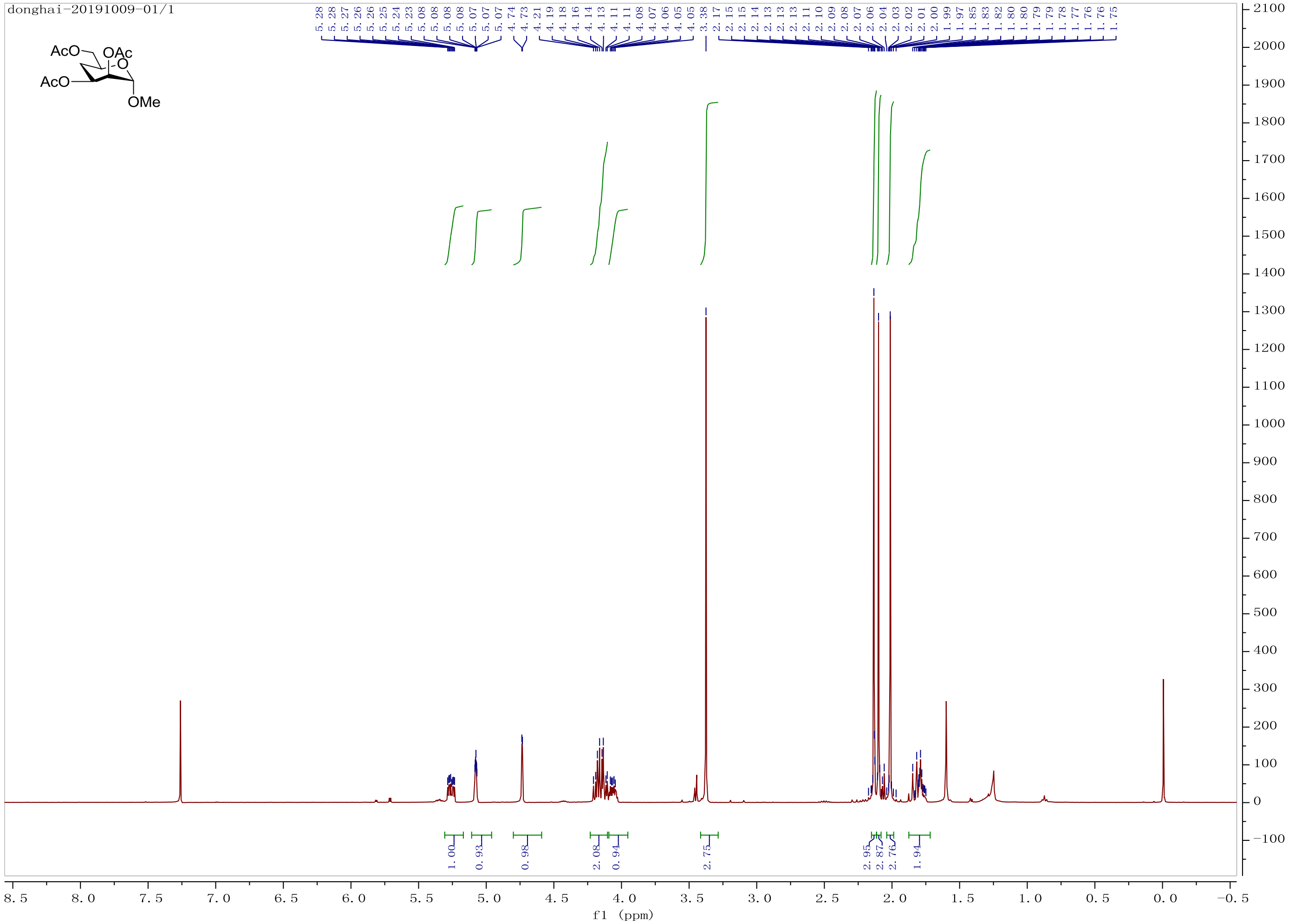

Figure S51. ^{1}H NMR spectrum (400 MHz) of **46** in $CDCl_3$

Methyl 2,3,6-tri-*O*-acetyl-4-deoxy-*β*-D-lyxo-hexopyranoside 47

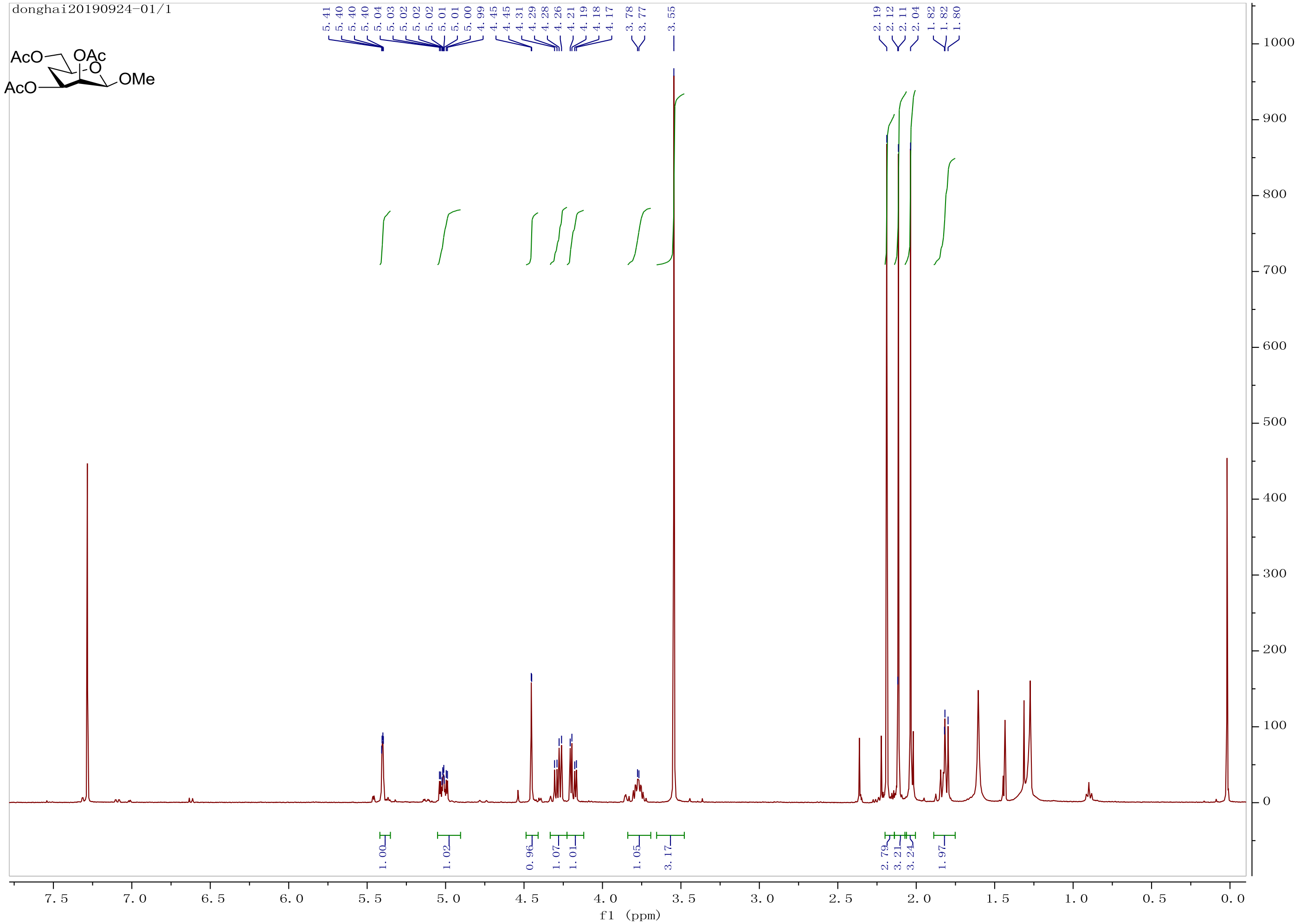

Figure S52. ^{1}H NMR spectrum (400 MHz) of **47** in $CDCl_3$

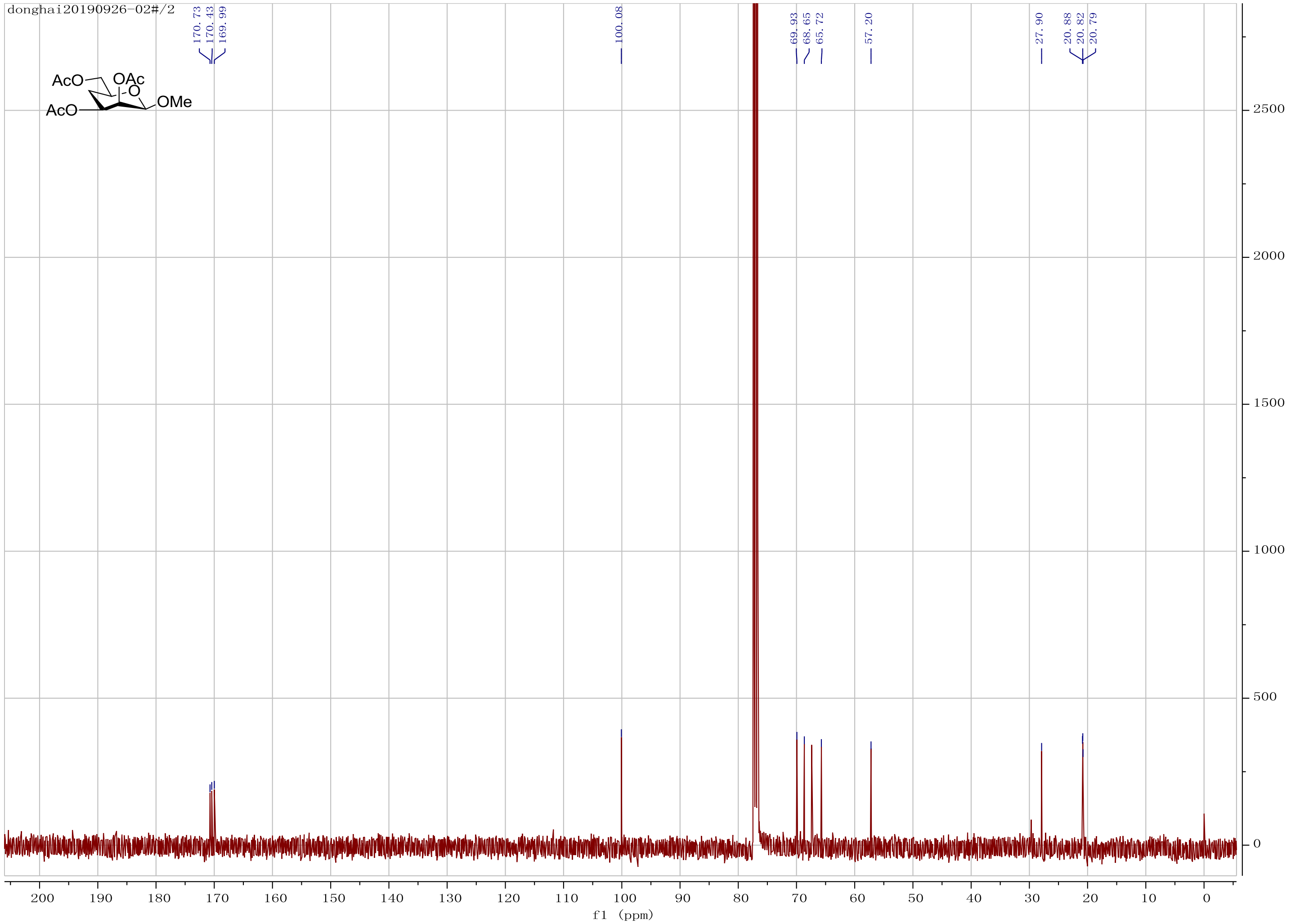

Figure S53. ^{13}C NMR spectrum (100 MHz) of **47** in $CDCl_3$

Methyl 3,6-Di-*O*-acetyl-2,4-dideoxy-*α*-D-threo-hexopyranoside 48

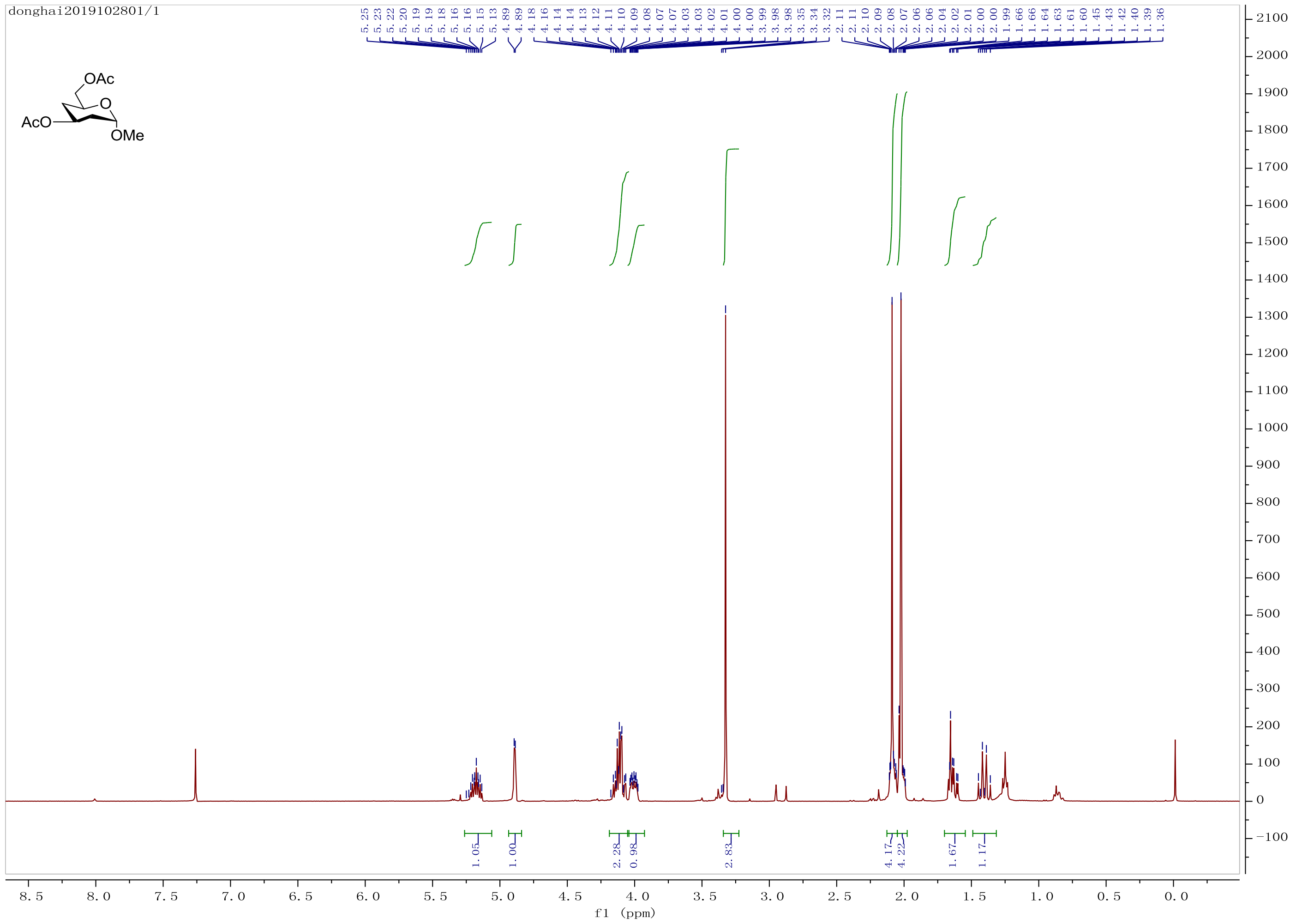

Figure S54. ^{1}H NMR spectrum (400 MHz) of **48** in $CDCl_3$

Methyl 3,6-Di-*O*-acetyl-2,4-dideoxy-*β*-D-threo-hexopyranoside 49

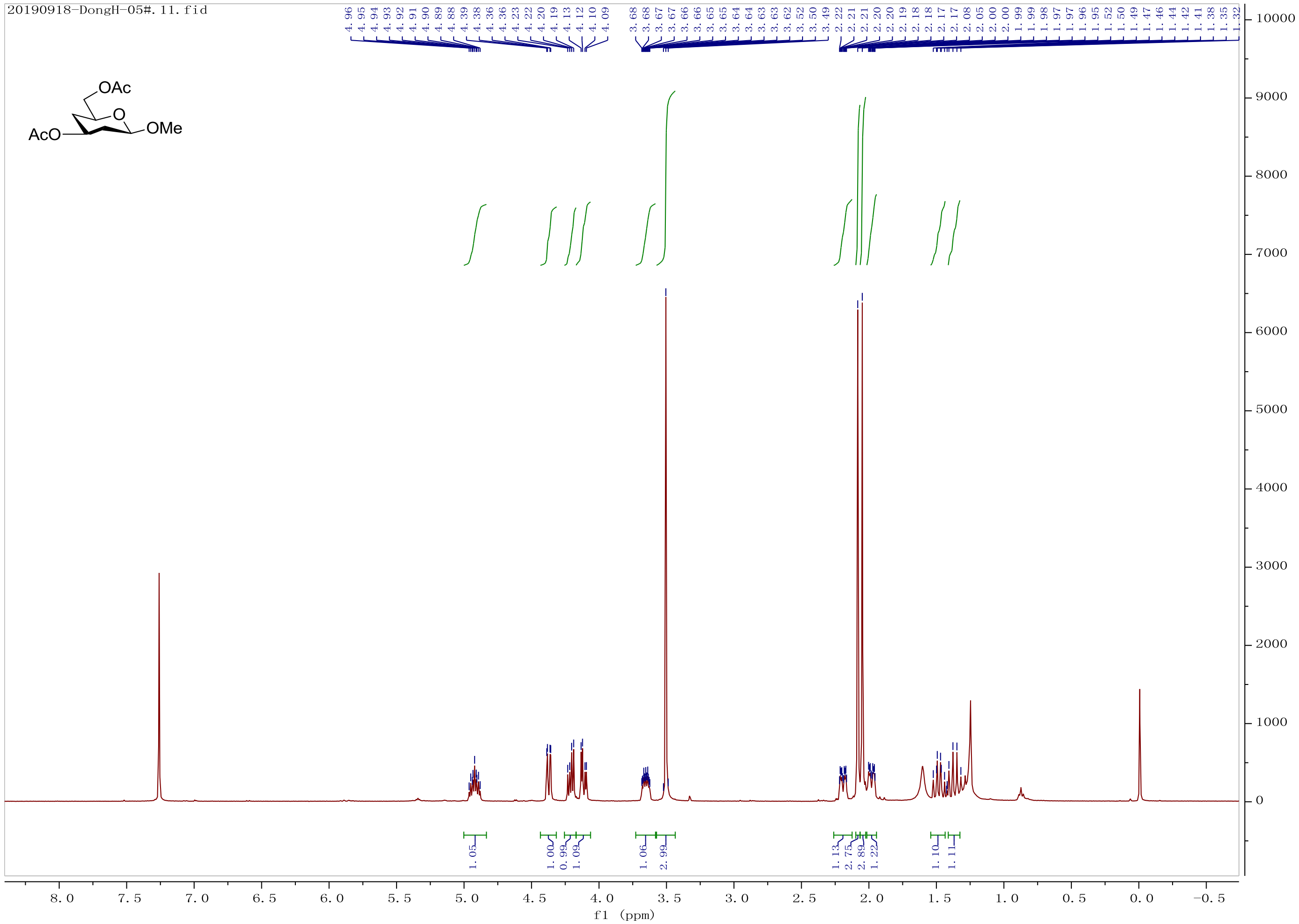

Figure S55. ^{1}H NMR spectrum (400 MHz) of **49** in $CDCl_3$
